# Comparison of the phenolic and antioxidant potential of five European herbal remedies by effect-directed analysis using offline two-dimensional liquid chromatography-high resolution mass spectrometry

**DOI:** 10.1007/s00216-026-06319-2

**Published:** 2026-01-28

**Authors:** M. Häßler, K. Wetzel, T. Tishakova, N. Dimitrova, T. Niedenthal, L. Montero, J. F. Ayala-Cabrera, O. J. Schmitz

**Affiliations:** 1 Applied Analytical Chemistry, University of Duisburg-Essen, Universitaetsstr. 5, 45141 Essen, Germany; 2 Forschergruppe Klostermedizin GmbH, Annastr. 26a, 97072 Würzburg, Germany; 3 Foodomics Laboratory, Institute of Food Science Research – CIAL (CSIC-UAM), Calle Nicolás Cabrera 9, 28049 Madrid, Spain; 4 Department of Analytical Chemistry, University of the Basque Country (UPV/EHU), Sarriena Auzoa, 48940 Leioa, Spain; 5 Research Centre for Experimental Marine Biology and Biotechnology, University of the Basque Country (PiE-UPV/EHU), Areatza Hiribidea 47, 48620 Plentzia, Spain

**Keywords:** Bioactive compounds, Preparative LC, European medicinal plants, 2D-LC, Non-targeted analysis

## Abstract

Herbal remedies contain various phenolic compounds. However, it remains difficult to identify the most important bioactive components and compare their effectiveness in different plant species. In this study, effect-directed analysis has been applied to five European medicinal plants. *Angelica archangelica*, *Angelica sylvestris*, *Agrimonia eupatoria*, *Sambucus ebulus*, and *Sambucus nigra* have been analyzed to unravel and compare their phenolic profiles and antioxidant potential. Plant extracts obtained by a sustainable microwave-assisted extraction method were fractionated using semi-preparative liquid chromatography to yield continuous fractions, and a miniaturized ABTS radical scavenging assay of the fractions was used to screen for antioxidant activity. Highly active fractions were selected for a second HPLC fractionation and analyzed with a quadrupole time-of-flight mass spectrometer using a non-targeted workflow that successfully linked antioxidant effects to specific compounds or compound classes. In the richest antioxidant fractions, flavan-3-ol oligomers such as procyanidin C1 were found in *A. eupatoria*, the flavonol glycoside rutin and other co-eluting phenolics in S. nigra. In contrast, *A. archangelica* showed a distinct metabolite profile rich in coumarins (e.g., bergapten, umbelliferone), but they contributed less to antioxidant activity compared to the flavonoid-dominated profiles of the other species. Overall, leaves and flowers contained the highest diversity and quantity of phenolic antioxidants among the plants studied. The effect-directed analysis of multiple European medicinal plants demonstrated its utility in exploring the major antioxidant compounds and highlighted significant differences in phenolic composition and antioxidant activity between species and plant parts.

**Graphical abstract:**

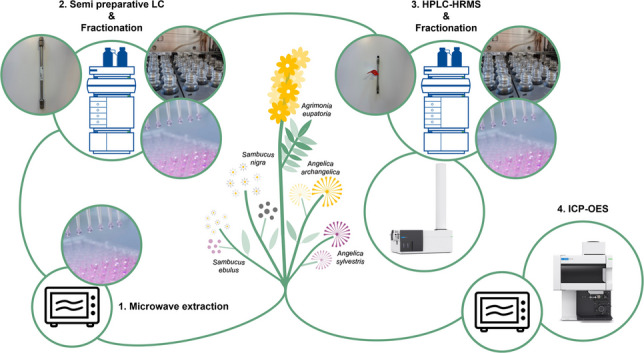

**Supplementary Information:**

The online version contains supplementary material available at 10.1007/s00216-026-06319-2.

## Introduction

Although herbal remedies have been used worldwide since ancient times to treat various diseases, nowadays there is a growing interest in the discovery of new medicines and active compounds [1, 2]. The growing demand for medicinal plant formulations is leading to increased interest in environmentally friendly manufacturing processes [3], harmonization within the European market [4], and regulations that facilitate access to traditional Chinese medicine in Europe, provided that the criteria for safety controls and pharmacological effects are met [3]. Health-promoting effects such as hepatoprotective or similar *in-vivo* activities have been reported for European medicinal plants such as *Angelica archangelica* and *sylvestris* [4, 5], *Agrimonia eupatoria* [6, 7], and *Sambucus nigra* [8, 9]. In *A. archangelica*, the essential oil extracted from the roots was primarily examined in order to identify the most common compounds [10, 11], while for *A. sylvestris*, the essential oils and methanolic extracts of aerial parts and roots were investigated [5]. *A. eupatoria* [12–16], *S. ebulus* [12–16], and *S. nigra* [9, 17–21] have been studied for over decades due to their hepatoprotective effects for the identification and determination of antioxidant activity of various plant parts. One of the challenges for the analysis of medicinal plants is the complexity of their metabolome that involves thousands of compounds. Besides, the questions about which compounds are the main drivers for the health-promoting effects and if they could be derived from specific plants or plant parts still remain unsolved. Furthermore, a comparison between different plants is advisable when their potential as herbal remedies is promoted, as extraction, identification, and quantification are often only carried out for individual species and may not be comparable. To correlate effects to active compounds, effect-directed analysis (EDA) is used as an analytical tool to screen and identify effect-driver compounds in a sample and, in regard to plants, for authenticity and identification of biomarkers [22–25]. For example, cultivated and wild-grown fresh and dried *S. nigra* berries were studied by high-performance thin-layer chromatography (HPTLC) coupled to mass spectrometry for confirmation and quantification of anthocyanins [26]. EDA involves several steps such as extraction, fractionation, and bioassays to screen for rich fractions which are later analyzed by targeted or non-targeted approaches with data processing aiming for the quantification and evaluation of the effect impact [25, 27]. In recent years, high-throughput EDA involved automatic feature prioritization among other data processing tools [28], automation of sample preparation [29], micro fractionation, and down-scaled bioassays [29], HPTLC [23], and online two-dimensional LC [25]. Even though this technique was widely applied and further developed in the environmental field, it was rarely applied to bioactive compounds found in food.

In our study, semi-preparative LC was used to enrich small abundant compounds, to reduce sample complexity for a chemical characterization, and to prioritize highly potent fractions before a second fractionation by high-performance liquid chromatography (HPLC) hyphenated to high resolution–tandem mass spectrometry (HRMS/MS). Various plant parts of five European herbal remedies (*Angelica archangelica*, *Angelica sylvestris*, *Agrimonia eupatoria*, *Sambucus ebulus*, and *Sambucus nigra*) were extracted by a sustainable microwave-assisted (MAE) method [30] and further analyzed using offline two-dimensional liquid chromatography. The antioxidant activity against 2,2'-azino-bis(3-ethylbenzothiazoline-6-sulfonic acid) (ABTS) in a miniaturized assay was determined for the individual plant parts flowers, buds, leaves, berries, stems, seeds, roots, and barks before and after continuous fractionation via semi-preparative LC. The fractions that exerted the highest antioxidant activity were further analyzed by HPLC-MS/MS for a chemical characterization via a non-target approach and were fractionated a second time using a different stationary phase to assign the activities to specific compounds or compound classes. This study aimed to unravel and compare the phenolic and antioxidant potential of European medicinal plants by EDA to identify the main health-promoting compounds. This is, to our knowledge, the first time that EDA has been applied to several parts of the plant and as a comparison between different European medicinal plants, as the only previous EDA approaches in the field have been reported for *S. nigra* berries exclusively. In this study, five herbal species were screened for TPC and antioxidant activity, a chemical characterization enabled a cross-species comparison for various anatomical parts, and the antioxidant activity was correlated to previously unknown or annotated compounds.

## Material and methods

### Materials

Ethanol (> 99.7% (*v/v*)) was purchased from VWR (Darmstadt, Germany). Methanol ($\ge$ 99.9% (*v/v*)) in HPLC-MS grade was supplied from VWR (Leuven, Belgium) and formic acid (≥ 99%) from Fisher Scientific (Schwerte, Germany). Cellulose filters type 15 A with a 110-nm diameter were obtained from Carl Roth (Karlsruhe, Germany) and PTFE filters with 0.20 µm pore size and a diameter of 13 mm from Macherey–Nagel (Düren, Germany). Ultrapure water (resistivity 18.2 M Ω cm^−1^) was daily obtained from an Ultrapure Water System (Sartorius, Goettingen, Germany). Sodium carbonate (Na_2_CO_3_, ≥ 99.5%) was purchased from AppliChem (Darmstadt, Germany). Potassium persulfate (≥ 99.0%), 6-hydroxy-2,5,7,8-tetramethylchroman-2-carboxylic acid (Trolox, 97%), and gallic acid (97.5%) were bought from Sigma Aldrich (Taufkirchen, Germany). Potassium dihydrogen phosphate (99.5%), di-sodium hydrogen phosphate, and Folin–Ciocalteau reagent were supplied from Merck (Darmstadt, Germany). 2,2’-Azino-bis(3-ethylbenzothiazoline-6-sulfonic acid) (ABTS) was bought from neoFroxx (Einhausen, Germany). Both assays were determined in triplicates.

For the elemental analysis, 65% nitric acid from Fisher Chemical (Schwerte, Germany) and 30% hydrogen peroxide from AppliChem GmbH (Darmstadt, Germany) were used. ICP elemental standards (Al, Ca, Fe, K, Mg, Na, P, S, Si, Y; 1000 mg/L in 2–3% HNO₃) from Merck (Darmstadt, Germany) served for calibration and quality control.

### Sample preparation

Dried flowers (Bosnia, 2021), berries (Poland, 2021), and barks (Serbia, 2020) from *Sambucus nigra L*. were obtained in a drug store (Herbathek, Berlin, Germany). *Sambucus nigra* leaves from Essen and flowers from three different cities, Marl, Haltern am See, and Essen, were collected in Germany in 2023 and air-dried at room temperature. Moreover, dried flowers (Poland, 2022) and berries (Poland, 2020) of *S. nigra*, leaves (Serbia, 2020) of *A. eupatoria*, leaves, seeds, and roots of *A. archangelica* (Poland, 2022), berries (Bulgaria, 2022), and roots (Poland, 2023) of *S. ebulus* were sponsored by Alfred Galke GmbH (Bad Grund, Germany). Dried leaves of *A. eupatoria* were obtained from Ttavu Ykrainy (Ukraine, 2023). Stems and flowers of *A. eupatoria*, flowers and stems from *A. archangelica*, and flowers and leaves of *S. ebulus* were collected in Bulgaria in 2024 and air-dried at room temperature. Whole plants of *A. sylvestris* were purchased in 2024 from Staudengaertnerei Gaissmayer (Illertissen, Germany) and taken apart into leaves, stems, and roots and air-dried at room temperature. For the extraction, 20%, 60%, or 90% (*v*/*v*) aqueous ethanol, depending on the plant material, was used as extraction solvent. Twenty percent was optimized for berries and seeds, 90% for *A. eupatoria* leaves, and 60% was optimal for other plant material based on previous results [30] and further experiments (data not shown). The extraction was carried out as described elsewhere [30]. Briefly, 125 mg of grounded plant material were extracted using 5 mL of solvent with an optimized microwave-assisted extraction (MAE) method in a microwave system (Mars NP-1185, Matthews, USA) for 5 min with a microwave power of 400 W at 55 °C. The extracts were stored after filtration at −80 °C. The Folin-Ciocalteu test was used for the determination of the total phenolic content (TPC) and for the antioxidant activity, a miniaturized assay using 2,2’-azino-bis(3-ethylbenzothiazoline-6-sulfonic acid) (ABTS).

For ICP-measurements, 0.5 g of ground material was weighed into Teflon vessels. Each vessel received 6 mL of 65% nitric acid and 2 mL of 30% hydrogen peroxide. Digestion was performed using a microwave system Mars NP-1185 (Matthews, USA) with a 15 min temperature ramp to 200 °C at 600 W, followed by 15 min at 200 °C. Blanks were processed identically, omitting the sample. After digestion, solutions were diluted to 50 mL with deionized water.

### Continuous fractionation by semi-preparative LC

The semi-preparative 1260 Infinity II LC consisted of a preparative autosampler (G157A), a preparative binary pump (G7161A), a fraction collector (G1364E), and a variable wavelength detector (VWD) with a 3 mm path length (G7114A-60024) and was controlled with OpenLab CDS Chemstation Edition Version 2.1.3 (Agilent Technologies). The first separation and fractionation were carried out using a Zorbax SB-C18 column (250 × 9.4 mm, 5-micron, Agilent, Santa-Clara, CA, USA). In cases where several extracts from different suppliers were available, the extract with the highest TPC and antioxidant activity was chosen for fractionation. The maximum possible volume of 900 µL was injected at a flow rate of 4 mL min^−1^ and the gradient was kept as follows: 0 min 5% B, 2 min 25% B, 12 min 95% B to 16 min with the mobile phases A: 5% (*v*/*v*) methanol in water and B: 100% methanol. A continuous fractionation was done from 2.5 to 14.5 min which covers the elution window over 1.5 min each, which corresponds to a volume of approx. 6 mL and results in eight fractions per extract with a total of 21 extracts being fractionated. The collected eluates were evaporated until dryness to determine the antioxidant activity against ABTS or were stored directly at −80°C until HPLC-HRMS analysis.

### Selective fractionation and chemical characterization by HPLC-HRMS/MS

The resulting 168 fractions were further separated using a HPLC 1290 Infinity system with a sampler (G4226A), a binary pump (G4220A), a TCC oven (G1316C), a fraction collector FC-AS (G1364C), and a DAD with a 10-mm path length and 1 µL volume (G4212A-60008). The maximum volume of 20 µL was injected onto a Kinetex® PFP column (100 × 2.1 mm, 1.7 µm, Phenomenex, Torrance, USA) at a flow rate of 0.3 mL min^−1^, with the mobile phase A being H_2_O and B being MeOH, both with 0.1% (*v/v*) formic acid. The gradient was set as follows: 0–2 min 5% B, 4 min 35% B, 8 min 35% B, 9.5 min 40% B, 14 min 45% B, 15 min 75% B, 23 min 95% B until 30 min. A 6546 Q-TOF mass spectrometer (Agilent, Santa Clara, USA) was configured to operate in positive mode with a mass range of 100–1700 m*/z*. The Agilent Jet Stream (AJS) parameters were set as follows: gas temperature of 320 °C, drying gas flow rate of 8 L min^−1^, nebulizer pressure of 35 psi, sheath gas temperature of 350 °C, and sheath gas flow rate at 11 L min^−1^. The capillary voltage was set to 3500 kV and fragmentor voltage to 175 V. Collision energy was applied using a formula accounting for *m/z* with a slope of 4.8 and an offset of 6. Instrument control was performed using Mass Hunter LC/MS Acquisition (version 10.1). The PFP column was additionally equipped with a 5-mm PFP guard column (Phenomenex, Torrance, CA, USA). For positive ESI ionization, the same solvents were used, while for negative ion mode experiments, water (A) and methanol (B) with 5 mM of ammonium formate and 0.1% (*v/v*) formic acid were used. The column was kept at a constant temperature of 50 °C and the flow was set at 0.4 mL min^−1^. The optimized gradient program was as follows: 5% B (held 1.6 min), up to 35% B at 2.6 min; up to 40% B at 6 min held for 1 min; up to 45% B at 10.5 min; up to 75% B at 11 min; up to 100% B at 17.5 min held for 2.5 min and goes back to initial conditions for a total run time of 22 min, while the injection volume was 5 µL. All extracts and fractions were measured in triplicates using a full scan data-dependent MS/MS acquisition mode for the chemical characterization. Data processing and analysis were conducted using MS-Dial 4.9 (10.1038/nmeth.3393) with parameters for data collection and peak detection as follows: MS^1^ tolerance was set to 0.01 Da, MS^2^ tolerance was set to 0.025 Da, minimum peak height for detection of a feature was set to 3000 in amplitude, while the peaks were smoothed with a linear moving average level 3. For compound identification and structural validation, acquired MS^1^ and MS^2^ data were compared against spectral databases such as the MassBank of North America (MoNA). Extracts and fractions measured in triplicates in positive and negative ionization modes were aligned to a pooled QC sample within the groups. Statistical analysis was done with MetaboAnalyst 6.0 [31].

Based on high ABTS results of LC fractions with values above 40 mg TE g^−1^ extract or overall high values among one plant part, ten fractions were chosen for a selective HPLC fractionation to further separate and characterize potential antioxidants. The threshold of 40 mg TE g^−1^ extract was selected as this was the highest value observed for the extracts before fractionation. The second fractionation was manually done in triplicates by HPLC with a PFP column as described above to enrich compound concentration before evaporation until dryness. One hundred twenty-five microliters of a 0.05 mM ABTS solution was added and visually compared to a reference vial with only reagent. The respective compound amounts were too low for quantitative results using the ABTS assay but were qualitatively tested for their antioxidant effect in order to enable a more precise correlation of antioxidant activity to the respective compounds analyzed by HRMS/MS.

### Elemental distribution with ICP-OES

Element quantification was performed using a 5900 ICP-OES instrument (Agilent, Santa Clara, CA, USA) operated in Synchronous Vertical Dual View (SVDV) mode with a viewing height of 8 mm. Instrumental parameters were as follows: read time, 5 s; RF power, 1.3 kW; stabilization time, 10 s; nebulizer flow rate, 0.8 L min^−1^; plasma flow, 13 L min^−1^; auxiliary flow, 2.0 L min^−1^; and injection pump rate, 6.4 mL min^−1^. Calibration curves were established using yttrium as an internal standard at 0.6 µg mL^−1^. Samples were diluted 1:10 (*v/v*) with 2% HNO_3_, and the internal standard was added after dilution. Each sample was measured in quintuplets at a pump speed of 12 rpm.

## Results and discussion

**Fig. 1:**
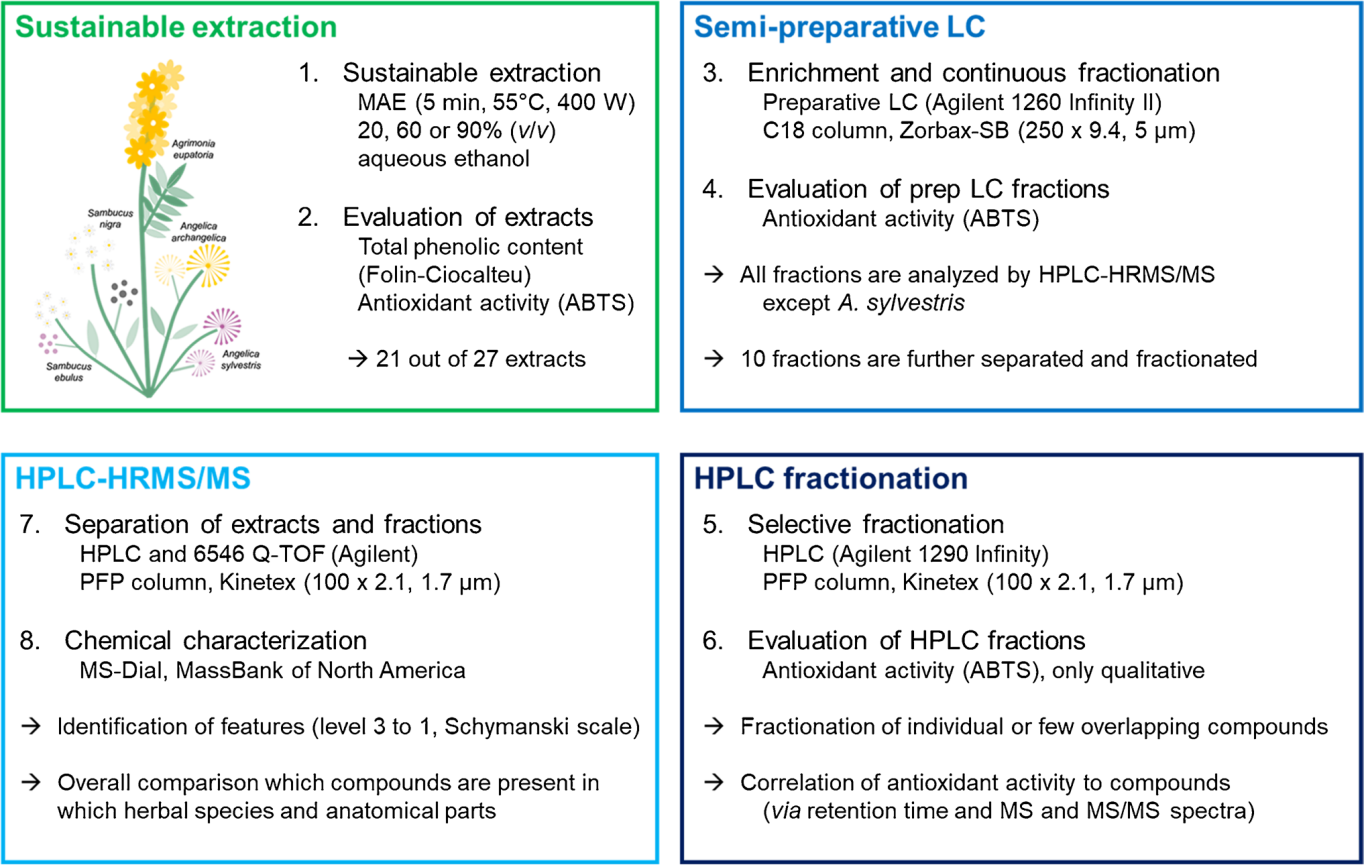
Overview of workflow for the evaluation of extracts and fractions from *A. angelica*, *A. sylvestris*, *A. eupatoria*, *S. ebulus*, and *S. nigra*, extracted by an optimized MAE method, enriched, and continuously fractionated by semi-preparative LC before further selective fractionation by HPLC and the chemical characterization by HPLC-HRMS/MS

The workflow consisted of four parts: the extraction by MAE, the continuous fractionation by semi-preparative LC, the selective fractionation by HPLC, and the chemical characterization by HPLC-HRMS/MS, as shown in Fig. 1. In addition to the chemical characterization, the elemental distribution was measured by ICP-OES.

### First continuous fractionation by preparative LC and prioritization

**Fig. 2:**
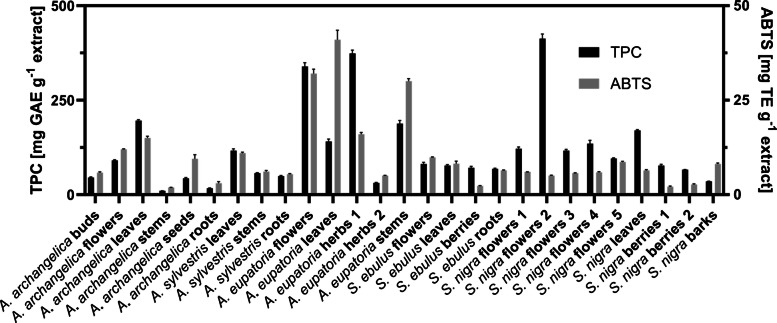
TPC and antioxidative activity against ABTS of 27 extracts from *A. angelica*, *A. sylvestris*, *A. eupatoria*, *S. ebulus*, and *S. nigra*, extracted by an optimized MAE method and determined in triplicates. Exact values of TPC and ABTS can be found in the Supplementary material Table S1

In total, 27 samples from five different European medicinal plants covering various plant parts such as flowers, leaves, buds, stems, berries, seeds, roots, and barks were extracted with a MAE treatment [30]. The TPC and antioxidant activity (ABTS) were determined for all plants and plant parts in triplicates (Fig. 2, Supplementary Table S1). For *A. angelica*, the highest TPC and ABTS results were achieved for the flowers, followed by leaves, buds, seeds, roots, and the stems, ranging from 10 to 196 mg GAE g^−1^ extract and from 3.0 to 15 mg TE g^−1^ extract, respectively. Compared to previously reported results for the roots of *A. archangelica*, 20 mg GAE g^−1^ were obtained after 10 h at 45 °C [32] while in this study, 17 mg GAE g^−1^ were reached after extraction by MAE for 5 min at 400 W and 55 °C. The TPC values of *A. eupatoria* samples ranged from 31 to 374, with the highest determined for one of the herbs, followed by flowers, stems, leaves, and the other herb sample. The highest ABTS values of all extracts were reached for the leaves, with 41. For the extraction using natural deep eutectic solvents, TPC values of the aerial parts of *A. eupatoria* showed values between 8 and 42 mg GAE g^−1^ after purification by SPE [33], while maceration over 72 h with various solvents yielded between 20 and 220 mg GAE g^−1^ [34]. *S. ebulus* had similar TPC values between 68 and 81 for the different plant parts, but the ABTS values ranged from 2.3 obtained by berries to 9.9 from flowers. *S. nigra* flowers had the highest TPC value of 413, while flowers from different suppliers reached 96–135. Interestingly, the ABTS values did not correlate, as the highest, with 8.9, was obtained from the flowers with the lowest TPC and vice versa. As observed before, leaves had also high TPC and ABTS values (170 and 6.4). For both *Sambucus* species, the roots or barks exerted higher antioxidant activities than the berries. As a comparison between *S. ebulus* and *S. nigra* plant parts extracted with 60% aqueous ethanol for 1 h at 60˚C, TPCs of 98 mg GAE g^−1^ extract and 179 were reported for the flowers and 116 and 178 for leaves [35], respectively. *S. ebulus* was found to have lower values compared to *S. nigra* [35], which was confirmed by our study except for the berries that had similar values of 71 and 77 mg GAE g^−1^ extract in TPC and 2.3 and 2.1 mg TE g^−1^ extract in ABTS.

**Fig. 3:**
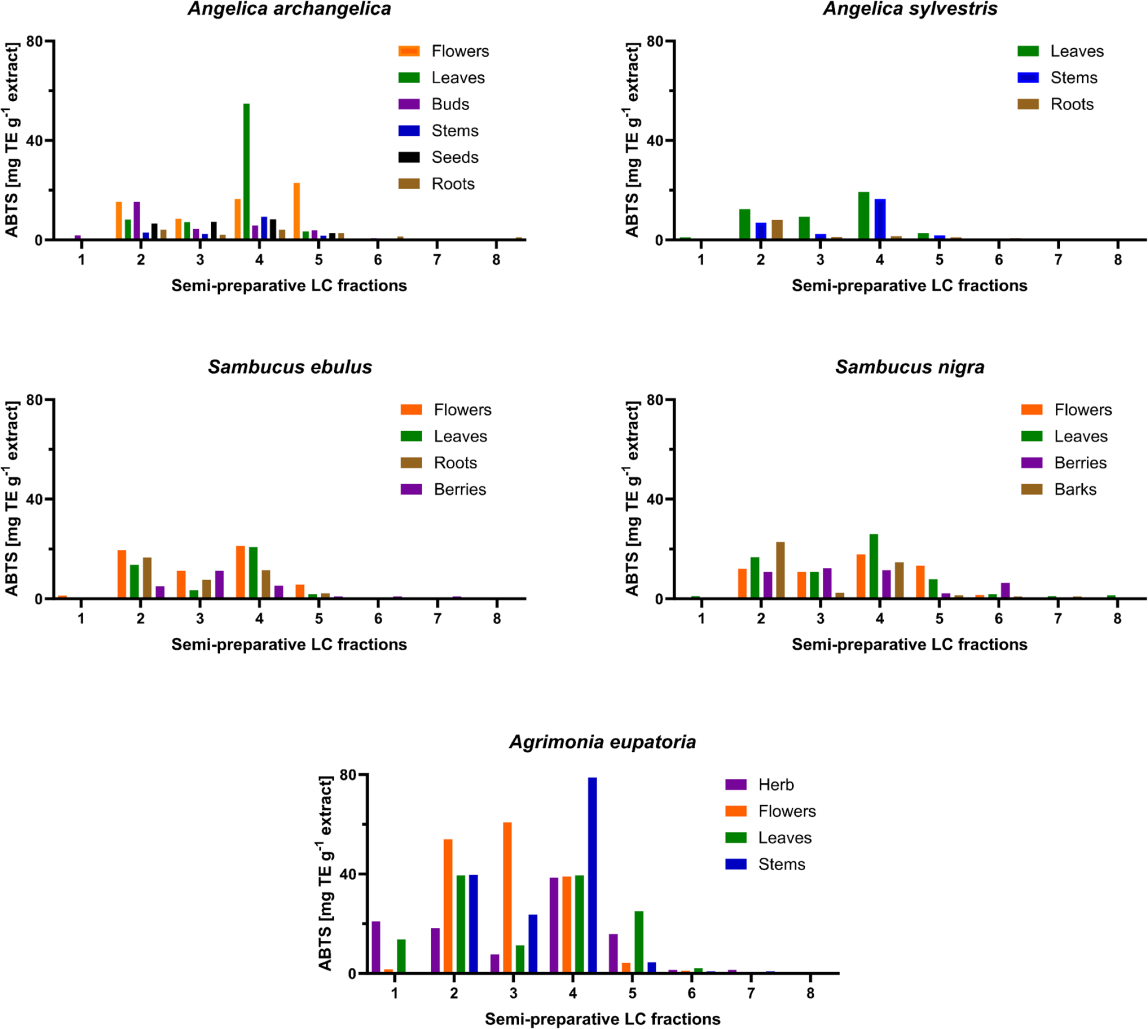
Quantitative ABTS results of 168 fractions collected by semi-preparative LC from *A. angelica*, *A. sylvestris*, *A. eupatoria*, *S. ebulus*, and *S. nigra*. Exact values can be found in the Supplementary material Table S1

As there were several extracts for some herbal species, the one with the highest values overall was chosen for fractionation, reducing the total number of extracts from 27 to 21. The first fractionation aimed to enrich compounds, collect continuous fractions, and screen them for their antioxidant activity to prioritize further analysis steps. The prep LC chromatograms mostly showed a unique chemical composition, with the flower and leaf extracts of each plant differing from each other in terms of their composition and/or intensity (see Supplementary material Figs. S1 and S2). Therefore, the fractions collected at the same time points for all extracts exhibited different antioxidant activities (Fig. 3). The results of the fractions revealed the following trends: first, the highest values were measured for *A. eupatoria* extracts. Second, leaves and flower extracts were more likely to reach high antioxidant effects. Third, the fractions 2 and 4 were overall higher in values compared to other fractions. In some cases, the third fraction showed antioxidative activity as well as for *A. eupatoria*. The leaves of *A. angelica* had the highest value in fraction 4, but the flowers in fraction 5. In opposite to that, *A. eupatoria* reached the highest value for the stems in fraction 4, the flowers in fractions 2 and 3, while the leaves are relatively high in fractions 2, 4, and 5. *A. sylvestris* showed the lowest values overall, with a similar trend as for *S. ebulus* and *S. nigra*. For *Sambucus* species, all values obtained for the different plant parts are similar, where also the roots and barks showed antioxidative activity in fraction 2, often even higher than for the leaves and/or flowers.

### Second selective fractionation and antioxidant activity

**Fig. 4:**
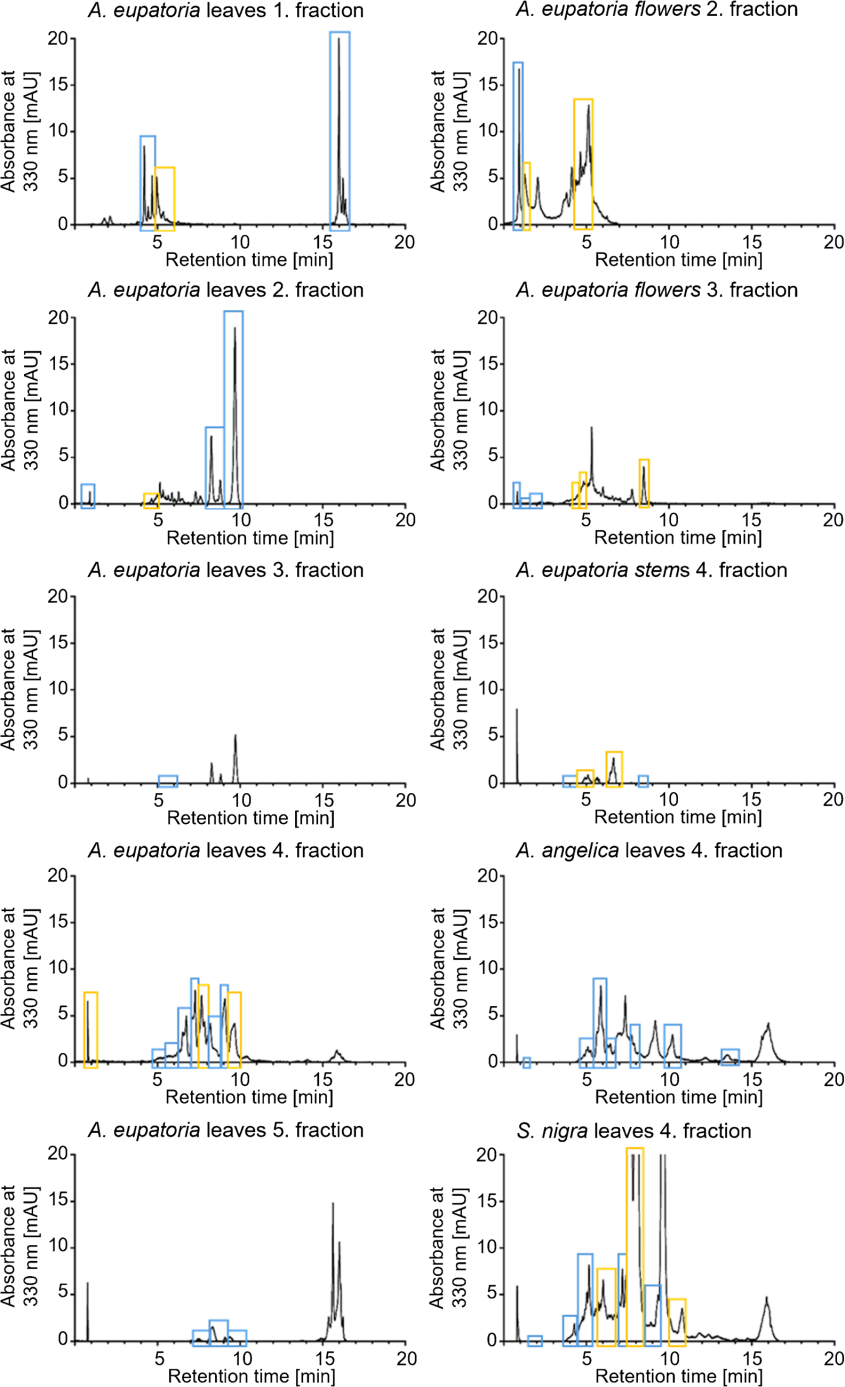
HPLC chromatograms of fractionated plant extracts measured at 330 nm. Second selective fractionation, as displayed with colored boxes, was based on HRMS data; blue states no reaction with ABTS, while yellow boxes indicate antioxidant activity. The qualitative color reaction with ABTS is pictured in Fig. S4 in the Supplementary material

Ten fractions were chosen for a second selective fractionation to compare chemical similarities of antioxidant compounds within one herbal species, *A. eupatoria*, and between the leaves of four species (Fig. 4). The DAD signal at an absorbance of 330 nm was recorded to monitor possible retention time shifts during fractionation as well as to visualize samples, as the differences were more prominent than in the TICs and BPCs (for TICs and BPCs, see Supplementary material Fig. S3). The time frames for the selective fractionation and the qualitative determination against ABTS can be found in the Supplementary Table S2 and Figure S4, with additional information about retention times and base peaks. The chromatograms with active compounds and no reaction with ABTS are summarized in with exact time points in the Supplementary Table S2. Despite the high ABTS values of *A. archangelica* leaves, the fractions of the HPLC analysis were no longer active due to the dilution, even though rutin was identified here as well.

Compounds that exerted an antioxidant effect were chemically characterized by their MS/MS spectra and summarized in Table 1 (for MS/MS spectra see Supplementary Material Figure S5). Generally, free phenolic -OH groups are essential for antioxidant activity, while derivatives that were attached to these free -OH groups tend to reduce antioxidant efficacy. The tentatively identified compounds include proanthocyanidins, whose ABTS scavenging activity strongly depends on their degree of polymerization. For example, procyanidin C1 (trimer, *m/z* 867.21) provides a higher activity than the dimer B1 (*m/z* 579.15, fragment *m/z* 291.08) or monomeric catechin (*m/z* 291.08), as demonstrated by their increasing radical-quenching capacity up to approximately 9 to 10 units [36]. A key structural factor is the free catechol B-ring of catechin and epicatechin subunits, which stabilizes radicals after hydrogen donation. Blocking one of these hydroxyl groups, for example, by O-methylation, significantly reduces the ABTS scavenging ability [37]. This structure-related activity is consistent across other flavonoid classes. In general, quercetin glycosides exhibit stronger radical scavenging activity than kaempferol glycosides, as quercetin possesses a catechol B-ring, whereas kaempferol has only one hydroxyl group at this position. Flavonol aglycones such as quercetin exhibit the greatest activity due to the combination of the catechol B-ring and the conjugated C-ring system, which enhances electron delocalization [37]. Simpler phenolic compounds such as caffeic acid can also exhibit high ABTS activity because they retain a catechol ring and an unsaturated side chain capable of stabilizing radicals. This also applies to chlorogenic acid, a caffeoylquinic acid derivative, where the caffeic acid monomers provide the same catechol-based radical stabilization, contributing significantly to its antioxidant capacity [36–38].

**Table 1:** Chemical characterization of antioxidative fractions collected by HPLC with their respective retention time, *m*/*z* ratio of base peak and annotation by in silico database comparison as level 3 based on the Schymanski scale [39]

Plant part	Prep LC fraction	Retention time HPLC [min]	*m*/*z* ratio	Compound/compound class	Additional notes
A. eupatoria leaves	1	5.059	323.0755	Flavonol	Similar to isorhamnetin
	2	4.47	579.1477 867.2123	Procyanidin C1	Found in flowers and stems of *A. eupatoria*
	4	1.102	121.0640	Phenylacetaldehyde	104.1067 in mass spectra (no reaction with ABTS)
		7.80	303.0489 465.1014 487.0833	Quercetin with deoxyhexose or hexose and glucose	Similar to isoquercetin
		9.70	447.0908 498.2587	Isomers of baicalin glycosides with sugar residues	Overlapping peaks
		9.86	447.0909		
A. eupatoria flowers	2	1.41	286.1034 136.0749 577.1323	Adenosine Unknown Unknown	ABTS reaction probably due to procyanidin C1
		4.48 4.87	579.1493 867.2128	Procyanidin C1	Same as in leaves and stems of *A. eupatoria*
	3	4.51	579.1489 867.2123	Procyanidin C1	Same as in leaves of *A. eupatoria*
		4.85	291.0858 579.1489	Procyanidin B1	With epi-, catechin monomer units visible
		8.40	463.0866	Kaempferol glucuronide	
A. eupatoria stems	4	4.85 5.35	291.0868 579.1510	Procyanidin B1	Found in flowers
		6.74	303.0502 597.1460 619.1279	Quercetin sugar residues	
S. nigra leaves	4	5.97	411.1989	Unknown	
		6.30	461.1918	Unknown	
		6.62	593.2208	Unknown	
		7.80	303.0500 611.1611 633.1434	Rutin	Found in *A. angelica* leaves with less intensity (no reaction)
		8.71	443.1889 603.2051	Unknown	
		9.16	595.1664 617.1485	Kaempferol-Hexosyl-Deoxyhexoside	Overlapping with chlorogenic acid glycoside
		9.28	355.1262 499.1242	Chlorogenic acid Dicaffeoylquinic acid	

For compounds that exerted an antioxidant effect and were tentatively annotated, the respective MS/MS spectra and a comparison between the spectra and their library matches are shown in the Supplementary Material Figures S5 and S6. The measured precursor ions show high agreement with the corresponding library spectra. All identifications fulfilled a mass accuracy threshold of 0.01 Da, and the spectral similarity (reverse dot product) exceeded 85%, supporting the reliability of the assignments across both positive- and negative-ion modes. The MS^1^ spectra of the examined components and tentatively identified metabolites are shown in Supplementary Material Figure S7. Distinct in-source fragments yield characteristic patterns that facilitate structural assignment. If components can be ionized in both polarities, the corresponding positive- and negative-ion MS^1^ spectra are shown side by side. Rutin exemplifies this, displaying the diagnostic *m*/*z* 303 fragment of quercetin, the two glycosidic fragments, and the sodium adduct at *m*/*z* 633 in positive-ion mode.

### Chemical characterization and comparison of five European herbal remedies by effected directed analysis

**Fig. 5:**
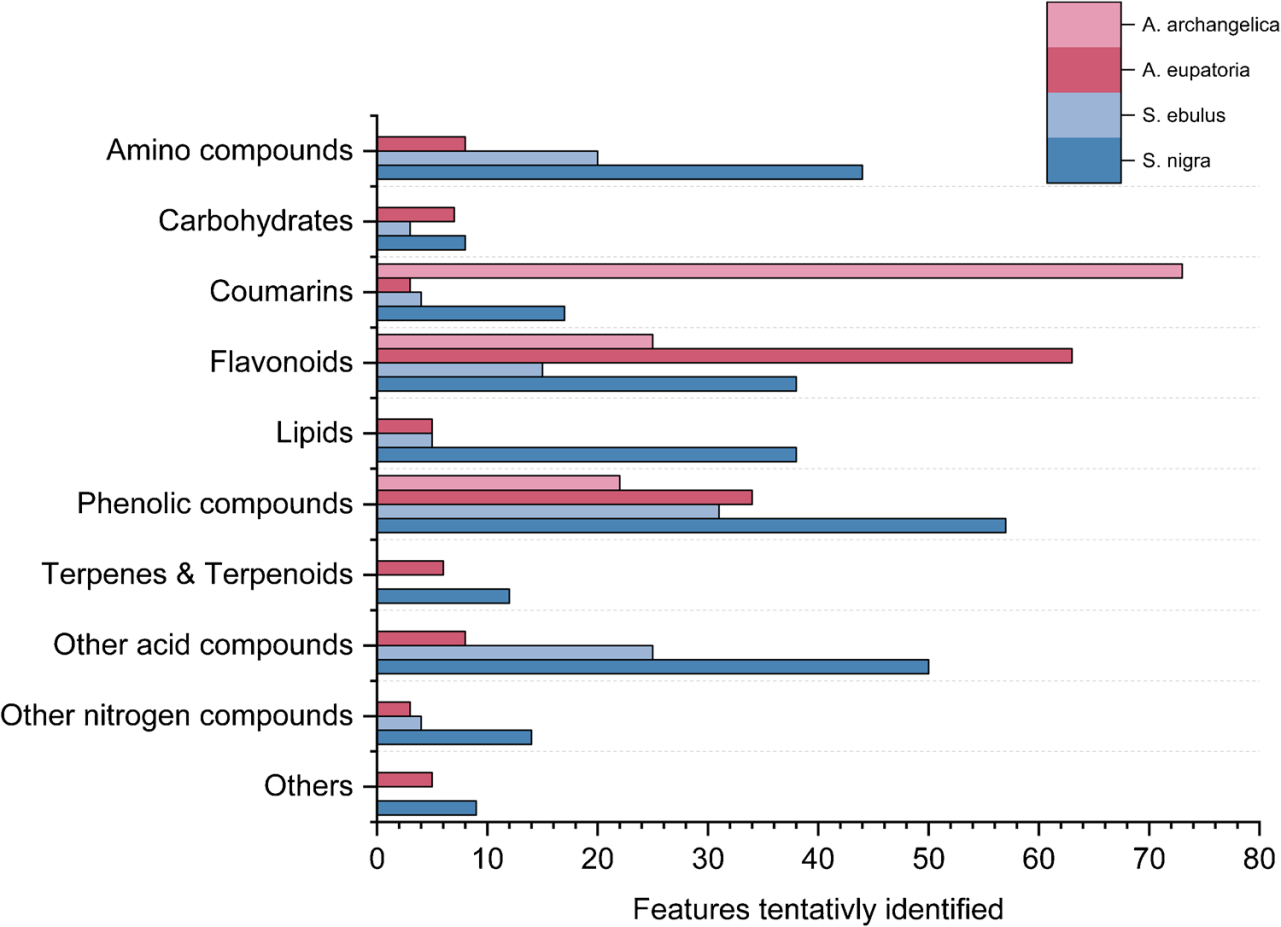
Identified metabolites across the analyzed plant extracts. Identification is based on annotated features in positive and negative ionization modes, multiple adducts, and matching in-source fragments. All compounds were grouped into seven major compound classes

Eleven extracts that had shown significant activity in previous bioassays were analyzed for plant metabolites using non-targeted chemical characterization. These included berries, bark, flowers, leaves, and roots from *S. nigra*, berries and roots from *S. ebulus*, leaves from *A. eupatoria*, and leaves, roots, and seeds from *A. archangelica*. After filtering, 1123 MS library matches were detected at a significance threshold of > 85%. After removing features that were tentatively identified as synthetic or anthropogenic compounds (~ 5.2%) and subtracting features that were measured in the blanks (~ 7.7%), 978 features were obtained. Positive and negative features were merged within a retention time range of < 0.05 min, in-source fragmented features and adducts within the same retention time range, and a calculated mass error of < 0.01 Da (approx. ~ 30 ppm). The final dataset comprised 663 features which were classified as Schymanski level 3 [39] or higher as listed in the Supplementary material Tables S5–S8. These compounds can be divided into seven main groups to simplify the overview and focus on the most important compounds with antioxidant effects (see Supplementary Table S4). For *A. archangelica*, *A. eupatoria*, *S. ebulus*, and *S. nigra*, 121, 144, 106, and 290 compounds were identified (level 3-1), respectively. *A. archangelica* led to the biggest differences among the plants due to the high content in coumarin compounds found*.* Flavonoids are mainly found in *A. eupatoria*, *S. ebulus*, and *S. nigra* as the major phenolic group. Both flavonoids and coumarins are classes of phenolic substances that contribute to plant defense mechanisms, and they have been associated with health benefits, particularly their anti-inflammatory activities [40]. The main compound classes tentatively identified for the plants are summarized in Fig. 5 and a distribution of tentatively identified compounds based on plant parts is shown in Fig. S8. This clearly demonstrates the increased number of features found in leaves and flowers.

Seventy-eight features were identified as level 1 (Table 2) by a retention time match with the respective standard. 81 features were identified as level 2, determined by an annotated feature in positive and negative polarity as well as multiple adducts and the occurrence of in-source fragment features aligned with the compound retention time. All other features were classified at identification level 3. Further information on level 3 to 1 identified features is available (Supplementary material Tables S5-S8). Several trends can be observed, for example, *A. archangelica* contained several coumarins, including coumarin, umbelliferone, bergapten, psoralen, and angelicin, also known as isopsoralen, as previously reported [10, 11, 41].

**Table 2:** Identified compounds, level 1 on the Schymanski scale, due to retention time and HRMS/MS match of the respective compounds found in the extracts with standards

*A. archangelica*	*A. eupatoria*	*S. ebulus*	*S. nigra*
Bergapten	4-Coumaric acid	2,5-Dihydroxybenzoic acid	4-Hydroxybenzoic acid
Caffeic acid	Adenosine	4-Coumaric acid	Adenosine
Coumaric acid	Apigenin	Adenosine	Asparagine
Coumarin	Caffeic acid	Asparagine	Aspartic acid
Ferulic acid	Catechin	Bergapten	Caffeic acid
Hesperetin	Chlorogenic acid	Caffeic acid	Catechin
Isoquercetin	Isoquercetin	Catechin	Chlorogenic acid
Naringenin	Kaempferol	Chlorogenic acid	Citric acid
Rutin	Kynurenic acid	Citric acid	Ferulic acid
	Luteolin	Ferulic acid	Fumaric Acid
	Naringenin	Fumaric acid	Gallic acid
	Phosphoric acid	Leucine	Isoquercetin
	Rutin	Naringenin	Kaempferol
	Tryptophan	Nicotinic acid	Kynurenic acid
	y-Linolenic acid	Phenylalanine	Leucine
		Phosphoric acid	Naringenin
		Proline	Nicotinic acid
		Pyruvic acid	Palmitic acid
		Rutin	p-Coumaric acid
		Stearic acid	Phenylalanine
		Succinic acid	Proline
		Tryptophan	Pyruvic acid
		Tyrosine	Rutin
			Stearic acid
			Succinic acid
			Tryptophan
			Tyrosine
			Vaccenic acid
			Valine
			Vanillin
			y-Linolenic acid

Coumarin originates from phenylalanine and via the phenylpropanoid metabolic pathway, progressing via cinnamic acid and p-coumaric acid to p-coumaroyl-CoA [42]. From there, umbelliferone, a simple coumarin, is produced as the central precursor. By further hydroxylation and methylation reactions, umbelliferone forms esculetin, scopoletin, isofraxidin, and fraxidin [43]. These compounds undergo glycosylation to form derivatives such as esculin or fraxin. Umbelliferone can also undergo prenylation, resulting in either linear furanocoumarins (e.g., psoralen, bergapten, xanthotoxin, isopimpinellin, and imperatorin) or angular furanocoumarins (e.g., angelicin, columbianetin, archangelicin, xanthyletin, osthol, auraptenol, tomasin, and rutarin) [42, 44]. These are characteristic of most *Angelica* species. All of these compounds were at least tentatively identified with a level higher than 3. This clearly demonstrates the furocoumarin pathway of *A. archangelica*, as confirmed by previous studies [41]. However, it is not yet possible to distinguish, for example, angelicin from psoralen due to the mass and fragments. Additionally, flavonoids such as naringenin and its glycosidic derivatives, as well as diosmetin derivatives like the diglucoside diosmin, have been tentatively identified. In *A. archangelica*, furanocoumarins and flavonoids such as bergapten, naringenin, and rutin were mostly detected in fraction 4. Most of them are found in the leaves, which also shows the highest radical-scavenging activity, and especially bergapten is known for its strong antioxidant and anti-inflammatory effects [45]. Naringenin derivatives have also been shown to have anti-inflammatory effects in several studies [46]. For example, it has been demonstrated that naringenin significantly reduced lipid peroxidation and restored antioxidant defense levels [47] or showed radical scavenging activity for biomolecule protection [48]. *A. eupatoria* primarily contains flavonoids, such as naringenin, as well as flavones, such as luteolin, and flavonols, such as quercetin and kaempferol. Flavonoids found in the analysis could be briefly explained while looking at their biosynthesis pathway, which also begins with p-coumaroyl-CoA. It is then metabolized to the central flavanone naringenin via chalcone, which serves as the precursor for subsequent flavonoids. Naringenin can be oxidized to dihydroflavonols, including taxifolin, dihydrokaempferol, and dihydroquercetin. These dihydroflavonols can then be converted into flavonols, such as kaempferol and quercetin. Various processes, such as glycosylation, glucuronidation, malonylation, or acetylation, modify these and form kaempferol-3-O-rutinoside, rutin, isoquercetin, tiliroside, and quercetin-malonylglucoside, among others. Naringenin also produces flavones, such as apigenin, luteolin, baicalin, diosmin, diosmetin, and acacetin. These flavones are glycosylated by various sugar residues, resulting in metabolites like vitexin, isovitexin, and plantaginin [49]. Additionally, flavan-3-ols, such as catechin and procyanidin B, are formed by reducing flavanones and polymerizing them into procyanidins, which were also found in *A. eupatoria* as previously reported [50, 51]. As already explained for the coumarins, these compounds can only be tentatively identified as it is impossible to distinguish between flavones, such as luteolin, and kaempferol using HRMS. However, several different flavonoids and their glycosides are present. This can be inferred based on the TPC content shown in Fig. 2. The significant elevated ABTS activity observed in *A. eupatoria* fractions is consistent with the presence of oligomeric procyanidins. These are primarily found in fraction 4 of the *A. eupatoria* leaves and showed the highest intensity of the leaves sample for the ABTS results. As explained in the section, procyanidins exhibit an enhanced electron transfer capacity, characterized by an increasing degree of polymerization [36, 37]. In the case of *S. ebulus*, a limited range of flavonoids, primarily containing naringenin, quercetin derivatives, catechin, isorhamnetin, and kaempferol/luteolin derivatives, were found. Assuming flavonoids comprise most of the TPC results, the identification results would align with Fig. 2. *S. nigra*, particularly its flowers, showed the most tentatively identified features. Many different quercetin, luteolin, kaempferol, isorhamnetin, cyanidin, and naringenin derivatives, as well as coumarins such as umbelliferone, were present and allow for direct insights into the metabolomic pathways of S. nigra. Interestingly, sambunigrin, which is a specific compound found in elder species, has been tentatively identified. Overall, more molecular features are observed in negative ionization mode compared to positive mode, suggesting that the main families that are observed have a high acidity, e.g., acids, hydroxyls, and phenolics. Phenolics also have a low proton affinity and radical scavenging properties and therefore contribute to the ABTS results [52]. For *A. eupatoria*, *S. ebulus*, and *S. nigra*, most features were found in fractions 2 and 4, while only a few were found in fractions 5 to 8 (see Supplementary material Fig. S7). For *A. archangelica*, most features were found in fraction 5, correlating with the ABTS assay results (Table 1). The strong ABTS observed in *Sambucus* extracts is consistent with the presence of caffeoyl and chlorogenic acids, which contain catechol groups, quercetin derivatives, and kaempferol derivatives. Chlorogenic acid derivatives are most frequently observed in fraction 3 and primarily in the berries. Quercetin and kaempferol-type flavonoids are found in fractions 4 and 5. Especially, quercetin is known for their radical-scavenging efficacy, which is due to their 3’,4’-dihydroxylation pattern [37]. Quercetin derivatives, including rutin, are present in high concentrations in the flowers and leaves and show, as already often described, high antioxidant capacity reducing inflammation [53–55]. Kaempferol derivatives are also known for their anti-inflammatory effects [46] and show a similar localization within the plants as quercetin.

**Fig. 6:**
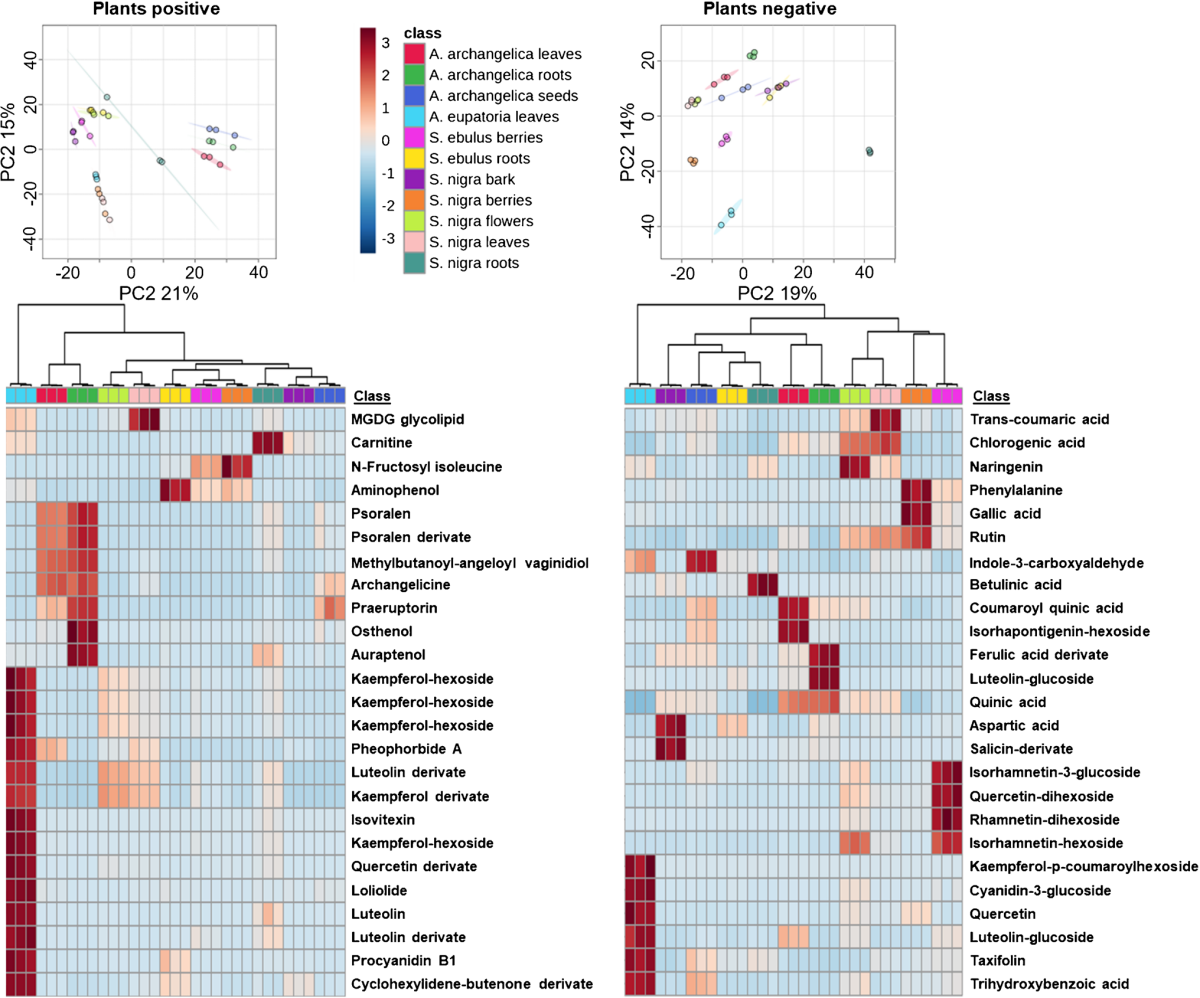
Principal component analysis and hierarchical clustering of positive- and negative-ion LC–MS/MS datasets from the investigated plant extracts. The PCA score plots (top) show clear group separation based on metabolite profiles in positive (left) and negative (right) ionization mode. Corresponding heatmaps (bottom) show normalized feature intensities, highlighting class-specific marker distributions and clustering patterns across all samples

Furthermore, a multivariate analysis of the LC–MS/MS extracts was conducted, highlighting distinct differences among the investigated plant samples. As shown in Fig. 6, PCA separates the samples in both ionization modes, reflecting clear differences in their metabolic fingerprints. The corresponding heat maps reveal consistent clustering patterns within each extract, indicating that the intensity distributions of individual compound classes vary in a systematic manner.

Compounds such as procyanidin B1, archangelicine, psoralen, naringenin, chlorogenic acid, coumaric acid, gallic acid, and various quercetin derivatives (*e*.*g*., rutin) were detected and show distinct differences between the plant parts. Additionally, several kaempferol hexosides at the retention times 3.7, 4.0, 5.6, and 7.6 min are detected. The early eluting hexosides are probably because of in-source fragmentation of di- or trihexosides with kaempferol, fitting well with the Tables S5, S6, S7, and S8. Previously identified metabolites reappeared in these profiles, confirming their suitability as specific markers for distinguishing the plant species and their respective parts. Another example would be pheophorbide A, as a byproduct of chlorophyll breakdown, which was found in high concentrations exclusively in leaf material.

### Elemental distribution of selected European herbal remedies

**Fig. 7:**
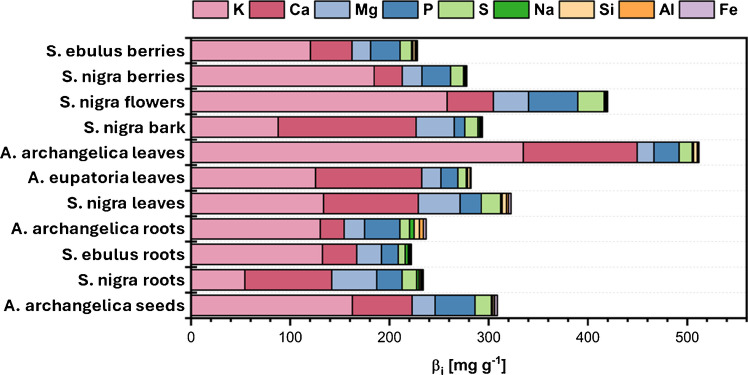
Elemental composition of elements of the dissolved sample for each plant part measured in ICP-OES, sorted by plant parts

For extracts studied in detail, ICP-OES was applied for elemental analysis. As demonstrated in Fig. 7, the mean distribution of elements differs considerably between various plants and plant parts. In general, Ca, K, Mg, P, and S were most abundant in the plants. The presence of calcium was particularly notable in the roots, bark, and leaves, especially in the parts that define the shape of the plant, suggesting that it plays a role in providing structural support for cell walls and membranes [56]. Potassium showed the highest concentrations in flowers and berries, likely supporting fruit formation and water transport, as has been observed in various other plants. The distribution of magnesium within the plants was uniform. Magnesium is the central atom of chlorophyll and therefore is essential for photosynthesis and more than 300 other metabolomic processes within plants [57, 58]. Phosphorus is required for the synthesis of ATP, DNA, RNA, and several other processes. A deficiency can lead to reduced plant growth and fruit formation [59]. This is probably the reason why flowers, seeds, fruits, roots, and leaves contain the highest concentrations of phosphorus. Sulfur was found to be highly concentrated in leaves and flowers. Sulfur is a component of amino acids such as cysteine and methionine, as well as vitamins and coenzymes, supporting protein synthesis and enzyme activation [60].

## Conclusion

Effect-directed analysis using offline two-dimensional liquid chromatography–high resolution mass spectrometry of five European medicinal plants enabled a direct comparison of their phenolic profiles and antioxidant potentials. Overall, leaves and flowers emerged as the richest in phenolic constituents and exhibited the strongest ABTS radical scavenging activities, whereas stems, roots, and other parts generally showed lower values. Among the species, *A. eupatoria* stood out with the most antioxidant fractions, notably in its flowers and stems, correlating with a high abundance of flavan-3-ol oligomers (epicatechin, procyanidin). In particular, the trimeric procyanidin C1 was found and tentatively identified by in silico fragmentation, and the monomeric catechins in the non-targeted approach, in active fractions of *A. eupatoria*. It is attributed as a major contributor to its higher antioxidant effect. By contrast, *A. archangelica* was characterized by high levels of coumarins, which distinguished the metabolome but contributed only modestly to antioxidant activity compared to the flavonoid-rich species. The two elder species, *S. ebulus* and *S. nigra*, showed intermediate antioxidant profiles: their berries and flowers contained substantial phenolics like chlorogenic acid, while *S. nigra* leaves showed detectable antioxidant effects linked to flavonol glycosides such as rutin and a kaempferol hexosyl-deoxyhexoside. Through targeted secondary fractionation of the most active fractions, the EDA approach successfully showed numerous bioactive compounds that could be responsible for the observed antioxidant effects. Examples include procyanidin B1 and kaempferol glucuronides in *A. eupatoria*, and chlorogenic acid derivatives as well as flavonol rutinosides in *S. nigra* leaves, each co-localized with significant ABTS activity. This links antioxidant efficacy to specific phytochemicals or substance classes, validating the EDA strategy for complex herbal extracts. Importantly, the offline coupling of semi-preparative LC to HPLC-HRMS enabled the enrichment, prioritization, and identification of potent compounds difficult to discover in un-fractionated extracts. In conclusion, this study demonstrates EDA as a powerful tool to break down complex plant matrices and associate biological effects with their respective chemical constituents. The comprehensive analysis across multiple species and plant parts highlights notable differences in antioxidant profiles (flavonoid- vs. coumarin-dominant) and identifies key compounds responsible for advantageous effects. Overall, the annotated compounds in the active fractions as well as the chemical characterization among the plant extracts and their fractions revealed differences among plant species and anatomical parts. Additionally, the fractions in which flavonoids were present correlate with the ABTS results. This not only advances our understanding of which compounds drive health-promoting antioxidant activity in herbal remedies but also showcases the value of EDA for guiding the discovery of bioactive constituents and supporting evidence-based use of traditional medicinal plants.

## Supplementary Information

Below is the link to the electronic supplementary material.

Supplementary file1 (DOCX 24.6 MB)

## Data Availability

All data are available to share upon request (Email: oliver.schmitz@uni-due.de).
