# Supplementary material for: Comparison of the phenolic and antioxidant potential of five European herbal remedies by effect-directed analysis using offline two-dimensional liquid chromatography-high resolution mass spectrometry
Source: Anal Bioanal Chem. 2026 Jan 28;418(5):1569–84. doi: 10.1007/s00216-026-06319-2 (PMC12909492; doi:10.1007/s00216-026-06319-2)
Supplement: Supplementary file 1 — Supplementary file1 (DOCX 24.6 MB) [file 216_2026_6319_MOESM1_ESM.docx]

**Comparison of the phenolic and antioxidant potential of five European herbal remedies by effect-directed analysis using offline two-dimensional liquid chromatography-high resolution mass spectrometry**

M. Häßler^a†^, K. Wetzel^a†^, T. Tishakova^a^, N. Dimitrova^a^, T. Niedenthal^b^, L. Montero^c^, J. F. Ayala-Cabrera^a,d,e^, O. J. Schmitz^a^*

^a^Applied Analytical Chemistry, University of Duisburg-Essen, Universitaetsstr. 5, 45141 Essen/Germany

^b^Forschergruppe Klostermedizin GmbH, Annastr. 26a, 97072 Würzburg, Germany

^c^Foodomics Laboratory, Institute of Food Science Research – CIAL (CSIC-UAM), Calle Nicolás

Cabrera 9, 28049 Madrid, Spain

^d^Department of Analytical Chemistry, University of the Basque Country (UPV/EHU), Sarriena Auzoa, 48940 Leioa, Spain

^e^Research Centre for Experimental Marine Biology and Biotechnology, University of the Basque Country (PiE-UPV/EHU), Areatza Hiribidea 47, 48620 Plentzia, Spain

*Corresponding author mail: [oliver.schmitz@uni-due.de](mailto:oliver.schmitz@uni-due.de); [lidia.montero@csic.es](mailto:lidia.montero@csic.es)

† These authors contributed equally to this work

**Tables**

**Tab. S1.** Overview of total phenolic content (TPC) and antioxidant activity against ABTS of various plant parts of *A. archangelica*, *A. sylvestris*, *A. eupatoria*, *S. ebulus*, and *S. nigra* and the ABTS values after fractionation using semi-preparative LC.

| **Species** | **Part** | **TPC** | **ABTS** | **ABTS of prep LC fractions** [mg TE g^-1^] | | | | | | | |
| --- | --- | --- | --- | --- | --- | --- | --- | --- | --- | --- | --- |
|  |  | [mg GAE g^-1^] | [mg TE g^-1^] | **1** | **2** | **3** | **4** | **5** | **6** | **7** | **8** |
| *A. angelica* | Flowers | 91 | 12 | 0.3 | 15 | 8.5 | 17 | 23 | 0.4 | 0.4 | 0.1 |
|  | Leaves | 196 | 15 | 0.1 | 8.2 | 7.1 | 55 | 3.4 | 0.5 | 0.5 | 0.5 |
|  | Buds | 45 | 5.8 | 1.8 | 15 | 4.4 | 5.8 | 3.8 | 0.7 | 0.4 | 0.2 |
|  | Stems | 10 | 1.9 | 0.1 | 3.0 | 2.4 | 9.3 | 1.7 | 0.2 | 0.2 | 0.2 |
|  | Seeds | 43 | 9.5 | 0.4 | 6.6 | 7.3 | 8.3 | 2.7 | 0.1 | 0.2 | 0.6 |
|  | Roots | 17 | 3.0 | 0.1 | 4.1 | 2.0 | 4.1 | 2.7 | 1.3 | 0.1 | 1.0 |
| *A. sylvestris* | Leaves | 117 | 11 | 1.0 | 12 | 9.3 | 19 | 2.7 | 0.4 | 0.4 | 0.1 |
|  | Stems | 57 | 6.1 | 0.2 | 6.9 | 2.4 | 17 | 1.8 | 0.6 | 0.6 | 0.2 |
|  | Roots | 49 | 5.4 | 0.6 | 8.0 | 1.1 | 1.5 | 1.0 | 0.7 | 0.6 | 0.3 |
| *A. eupatoria* | Flowers | 339 | 32 | 1.7 | 54 | 61 | 39 | 4.2 | 1.0 | 0.6 | 0.4 |
|  | Leaves | 141 | 41 | 14 | 39 | 11 | 39 | 25 | 2.1 | 0.5 | 0.2 |
|  | Stems | 188 | 30 | 0.3 | 40 | 24 | 79 | 4.4 | 0.9 | 0.1 | 0.3 |
|  | Herb | 374 | 16 | 21 | 18 | 7.7 | 39 | 16 | 1.5 | 1.0 | 0.4 |
| S. ebulus | Flowers | 81 | 9.9 | 1.3 | 20 | 11 | 21 | 5.7 | 0.4 | 0.1 | -0.1 |
|  | Leaves | 77 | 8.2 | 0.5 | 14 | 3.5 | 21 | 1.8 | 0.3 | 0.8 | 0.0 |
|  | Berries | 71 | 2.3 | 0.3 | 5.1 | 11 | 5.3 | 0.9 | 0.1 | 0.2 | 0.1 |
|  | Roots | 68 | 6.4 | 0.2 | 17 | 7.6 | 12 | 2.2 | 0.2 | 0.4 | 0.4 |
| S. nigra | Flowers | 413 | 5.0 | 0.4 | 12 | 11 | 18 | 13 | 1.5 | 0.8 | 0.9 |
|  | Leaves | 170 | 6.4 | 1.0 | 17 | 11 | 26 | 7.9 | 1.9 | 1.1 | 1.4 |
|  | Berries | 77 | 2.1 | 0.2 | 11 | 12 | 12 | 2.1 | 6.4 | 0.2 | 0.0 |
|  | Barks | 35 | 8.1 | 0.2 | 23 | 2.4 | 15 | 1.4 | 0.4 | 0.1 | 0.1 |

**Tab. S2.** Selected time points for second fraction using HPLC-HRMS with respective retention times and base peak *m*/*z* ratios of further analyzed compounds. ABTS reaction indicates according to color if the compound(s) had an antioxidant effect resulting in blue for no reaction at all, light blue for some antioxidant capacity or low concentration, colorless for antioxidant capacity and yellow as exception.

| **Sample** | **Prep LC fraction** | **Retention time [min]** | **Base peak *m*/*z* ratio of mass spectrum** | **Fractionation time points [min]** | **ABTS reaction** |
| --- | --- | --- | --- | --- | --- |
| ***A. angelica* leaves** | 4 | 1.224 | 268.1036 | 1.1-1.6 | blue |
|  |  | 5.011 | 222.1489 | 4.7-5.5 | blue |
|  |  | 5.951 | 447.1262 | 5.6-6.2 | blue |
|  |  | 6.544 | 215.0316  215.0316  245.0418 | 6.2-6.8 | blue |
|  |  | 7.831 | 611.1613  611.1613  633.1432 | 7.7-8.1 | blue |
|  |  | 10.351 | 609.1815 | 9.8-10.8 | blue |
|  |  | 13.769 | 525.1600 | 13.2-14.1 | blue |
| ***A. eupatoria* leaves** | 1 | 4.779 | 323.0755 | 4.0-4.9 | light blue |
|  |  | 5.059 | 323.0755 | 5.0-6.0 | colorless |
|  |  | 16.291 | 333.1038 | 15.5-16.5 | blue |
|  | 2 | 0.892 | 104.1067 | 0.7-1.4 | blue |
|  |  | 0.992 | 118.1012 |  |  |
|  |  | 4.471 | 579.1477 | 4.2-5.0 | colorless |
|  |  | 8.383 | 463.0858 | 8.0-9 | blue |
|  |  | 9.836 | 477.0913 | 9-10 | blue |
|  | 3 | 5.404 | 104.1062 | 5.0-6.0 | blue |
|  | 4 | 0.954 | 104.1067 | 0.7-1.5 | colorless |
|  |  | 1.107 | 121.0640 |  |  |
|  |  | 5.218 | 409.1822 | 4.7-5.5 | blue |
|  |  | 5.751 | 247.1300 | 5.5-6.2 | blue  blue |
|  |  | 5.898 | 219.0988 |  |  |
|  |  | 6.858 | 433.1121 | 6.2-7.1 | blue |
|  |  | 7.397 | 433.1118 | 7.1-7.5 | blue |
|  |  | 7.804 | 487.0833  465.1014  487.0833 | 7.5-8.1 | light blue |
|  |  | 8.344 | 449.1067  383.1460 | 8.1-8.9 | blue |
|  |  | 8.597 | 498.2588 |  | blue |
|  |  | 9.250 | 471.0891  303.0491  449.1067  471.0891  919.1920 | 8.9-9.4 | blue |
|  |  | 9.703 | 498.2587  447.0908  498.2587 | 9.5-10.0 | colorless |
|  |  | 9.857 | 447.0909 |  |  |
|  | 5 | 7.563 | 498.2581 | 7.0-8.0 | blue |
|  |  | 8.490 | 498.2583 | 8.0-9.0 | blue |
|  |  | 9.576 | 498.2587 | 9.0-10.0 | blue |
| ***A. eupatoria* flowers** | 2 | 0.852 | 104.1065  203.0522 | 0.7-1.2 | light blue |
|  |  | 0.932 | 116.0701 |  |  |
|  |  | 1.1012 | 130.0859 |  |  |
|  |  | 1.411 | 286.1034 | 1.2-1.6 | colorless |
|  |  | 4.477  4.870 | 579.1493  867.2128 | 4.2-5.2 | colorless |
|  | 3 | 0.879 | 104.1065 | 0.7-1.0 | blue |
|  |  | 1.025 | 136.0615 | 1.0-1.6 | blue |
|  |  | 1.926 | 120.0804 | 1.6-2.3 | blue |
|  |  | 4.512 | 579.1489  867.2123 | 4.3-4.7 | light blue |
|  |  | 4.851 | 291.0858  579.1489 | 4.7-5.1 | colorless |
|  |  | 8.399 | 463.0866 | 8.1-8.8 | colorless |
| ***A. eupatoria* stems** | 4 | 4.181 | 188.0710  291.0867 | 3.5-4.5 | blue |
|  |  | 4.847  5.354 | 291.0868 | 4.5-5.5 | colorless |
|  |  | 6.740 | 619.1279  597.1460  619.1279 | 6.2-7.1 | colorless |
|  |  | 8.513 | 287.1257 | 8.2-8.8 | blue |
| ***S. nigra* leaves** | 4 | 1.692 | 120.0806 | 1.4-2.2 | blue |
|  |  | 1.965 |  |  |  |
|  |  | 3.905  4.179 | 188.0708 | 3.6-4.4 | blue |
|  |  | 4.592  4.845 | 318.0952 | 4.4-5.3 | blue |
|  |  | 5.152 | 351.1051  409.1836 |  |  |
|  |  | 5.966 | 411.1989 | 5.7-6.8 | colorless |
|  |  | 6.299 | 461.1918 |  |  |
|  |  | 6.619 | 429.1730 |  |  |
|  |  | 7.319  7.459 | 499.1239  395.2040  499.1239  539.1164 | 7.0-7.5 | blue |
|  |  | 7.799 | 611.1611  611.1611  633.1434 | 7.5-8.4 | yellow |
|  |  | 8.713 | 443.1889 | 8.5-9.5 | colorless |
|  |  | 9.160 | 617.1485  617.1485 |  |  |
|  |  | 9.279 | 539.1167  499.1242  539.1167 |  |  |
|  |  | 10.206  10.380 | 609.1820 | 10.0-10.8 | blue |

**Tab. S3.** Overview of standard compounds that were screened for approval of level 1 identification by retention time and MS spectra comparison.

| **Acids** | | |
| --- | --- | --- |
| 2,5-Dihydroxybenzoic acid | Ferulic acid | p-Coumaric acid |
| 2-Oxoglutaric acid | Fumaric acid | Propionic acid |
| 2-Picolinic acid | Gallic acid | Pyruvic acid |
| 4-Acetoxybenzoic acid | Kynurenic acid | Salicylic acid |
| 4-Hydroxybenzoic acid | Linolenic acid | Sinapic acid |
| Angelic acid | Maleic acid | Stearic acid |
| Ascorbic acid | Malonic acid | Succinic acid |
| Caffeic acid | Myristic acid | Tannic acid |
| Chlorogenic acid | Nicotinic acid | trans-Cinnamic acid |
| Cholic acid | Oxalic acid | Vanillic acid |
| cis-Vaccenic acid | Oxaloacetic acid | γ-Linolenic acid |
| Citric acid | Palmitic acid | Phosphoric acid |
| DL-Isocitric acid |  |  |
| **Amino acids** | | |
| D-Leucine | L-Aspartic acid | L-Proline |
| DL-Phenylalanine | L-Cystine | L-Serine |
| DL-Tyrosine | L-Glutamine | L-Tryptophan |
| Glycine | L-isoleucine | L-Tyrosine |
| L-Alanine | L-Leucine | L-Valine |
| L-Asparagine | L-Methionine |  |
| **Others** | | |
| 2,4,6-Trihydroxyacetophenone | Curcumin | Quercetin hydrate |
| 3-Aminoquinoline | Delphinidin | Isoquercetin |
| 4-Hydroxybenzoicacidmethylester | Epicatechin gallate | Reserpine |
| 4-Nitrophenol | Guaiacol | Resveratrol |
| 6-(dimethylamino)purine | Hesperetin | Riboflavin |
| α-Pinene | Hesperidin | Rutin |
| Adenosine | Kaempferol | Syringaldehyde |
| Apigenin | Limonene | Taurine |
| α-Tocopherol | Linalool | Thymine |
| β-Pinene | Luteolin | Vanillin |
| Bergapten | Mannitol | Vitamin B12 |
| Caffeine | Myricetin | γ-Terpinene |
| Capsaicin | Naringenin | Cyanidin chloride |
| Catechin | Naringin | Morin |
| Cinnamaldehyde | Oleuropein | Silymarin mixture ***** |
| Colchicine | Polydatin |  |
| Coumarin | Pseudocapsaicin |  |

********Mix of Flavonolignans*

**Tab. S4.** Overview of main classes and subclasses of bioactive compounds for the identification workflow.

| **Main Class** | **Subclasses** |
| --- | --- |
| **Carbohydrates** | Sugars (monosaccharides, disaccharides), sugar alcohols, threose, polysaccharides |
| **Lipids** | Fatty acids (saturated/unsaturated), fatty acid derivatives, glycolipids, sphingolipids, phospholipids, steroids, lipid derivatives |
| **Amino Compounds** | Amino acids, amino acid derivatives, amines, amides, ammonium alcohols, quaternary ammonium compounds, peptides |
| **Phenolic Compounds** | Phenols, phenolic acids, flavonoids, isoflavonoids, anthocyanins, stilbenes, tannins, lignans, polyphenols, phenolic glycosides, phenolic aldehydes, phenylpropanoids, phenylethanoids |
| **Alkaloids** | Alkaloids (incl. tetrahydroisoquinolines, quinoline) |
| **Terpenes & Terpenoids** | Terpenes, terpenoids, terpenoid glycosides, carotenoids |
| **Coumarins** | Coumarins and derivatives, furanocoumarins, isocoumarins, coumarin glycosides |
| **Other Acid Compounds** | Carboxylic acids, dicarboxylic acids, hydroxycarboxylic acids, aromatic carboxylic acids, pyridinecarboxylic acids, organosulfonic acids, tricarboxylic acids, benzenedicarboxylic acids, aromatic carboxylic acids, butanoic acid derivatives |
| **Other Nitrogen Compounds** | Nucleosides, nucleotides, nucleobases, purines, pyrimidines, indoles, phenylethanolamines |
| **Others** | Aromatics (unspecified), aldehydes, ketones, esters, alcohols, surfactants, parabens, chelators, pesticides, vitamins, porphyrin derivatives, statins, xanthones, quinoline/indole derivatives, peptides |

**Tab. S5.** Overview of all compounds found in *A. archangelica*.

|  |  |  |  |  |  |  | Plant Part | | | | | Fraction | | | | | | | |
| --- | --- | --- | --- | --- | --- | --- | --- | --- | --- | --- | --- | --- | --- | --- | --- | --- | --- | --- | --- |
| Ontology | Compound Name | RT | Precursor mass | Adduct | Level | Polarity | Flowers | Berries | Leaves | Roots | Seeds | 1 | 2 | 3 | 4 | 5 | 6 | 7 | 8 |
| Coumarins | Bergapten | 11.564 | 217.0497 | [M+H]^+^ | 1 | [±] |  |  | x | x | x |  |  |  |  | x |  |  |  |
| Phenolic compounds | Caffeic acid | 3.618 | 179.0366 | [M-H]^-^ | 1 | [±] |  |  | x | x | x |  | x | x |  |  |  |  |  |
| Phenolic compounds | Coumaric acid | 4.093 | 163.0412 | [M-H]^-^ | 1 | [±] |  |  | x | x | x |  |  | x | x |  |  |  |  |
| Coumarins | Coumarin | 4.777 | 147.0437 | [M+H]^+^ | 1 | [±] |  |  | x |  |  |  |  | x |  |  |  |  |  |
| Phenolic compounds | Ferulic acid | 4.444 | 193.0517 | [M-H]^-^ | 1 | [±] |  |  | x | x | x |  |  | x | x |  |  |  |  |
| Flavonoids | Hesperetin | 10.811 | 303.0851 | [M+H]^+^ | 1 | [±] |  |  |  |  | x |  |  |  |  | x |  |  |  |
| Flavonoids | Isoquercetin | 5.174 | 465.1014 | [M+H]^+^ | 1 | [±] |  |  | x |  |  |  |  |  | x |  |  |  |  |
| Flavonoids | Naringenin | 10.05 | 271.0627 | [M-H]^-^ | 1 | [±] |  |  | x |  | x |  |  |  |  | x |  |  |  |
| Flavonoids | Rutin | 5.069 | 611.16 | [M+H]^+^ | 1 | [±] |  |  | x |  |  |  |  |  | x |  |  |  |  |
| Flavonoids | Apigenin-Hexoside | 6.736 | 431.0998 | [M-H]^-^ | 2 | [±] |  |  | x |  |  |  |  |  |  | x |  |  |  |
| Coumarins | Archangelicine | 12.615 | 449.1578 | [M+Na]^+^ | 2 | [±] |  |  | x | x | x |  |  |  |  |  | x |  |  |
| Flavonoids | Azaleatin-Hexoside | 6.563 | 477.1077 | [M-H]^-^ | 2 | [±] |  |  | x |  |  |  |  |  | x |  |  |  |  |
| Flavonoids | Diosmetin-O-hexosyl-deoxyhexoside | 6.967 | 631.162 | [M+Na]^+^ | 2 | [±] |  |  | x |  |  |  |  |  | x |  |  |  |  |
| Coumarins | Fraxidin | 4.516 | 223.0596 | [M+H]^+^ | 2 | [±] |  |  |  | x |  |  |  |  | x |  |  |  |  |
| Coumarins | Heralenol | 6.505 | 305.1014 | [M+H]^+^ | 2 | [±] |  |  | x |  |  |  |  |  |  | x |  |  |  |
| Coumarins | Imperatorin | 12.613 | 271.0958 | [M+H]^+^ | 2 | [±] |  |  |  |  | x |  |  |  |  |  | x |  |  |
| Flavonoids | Kaempferol-O-hexosyl-deoxyhexoside | 5.603 | 595.1647 | [M+H]^+^ | 2 | [±] |  |  | x |  |  |  |  |  | x |  |  |  |  |
| Coumarins | Methylbutanoyl-angeloyl vaginidiol | 12.741 | 451.1736 | [M+Na]^+^ | 2 | [±] |  |  |  | x |  |  |  |  |  |  | x |  |  |
| Coumarins | Oxypeucedanin hydrate | 9.319 | 305.1013 | [M+H]^+^ | 2 | [±] |  |  | x | x | x |  |  |  |  | x |  |  |  |
| Coumarins | Praeruptorin A | 12.342 | 409.1262 | [M+Na]^+^ | 2 | [±] |  |  | x | x |  |  |  |  |  | x | x |  |  |
| Coumarins | Scopoletin | 3.487 | 193.0492 | [M+H]^+^ | 2 | [±] |  |  | x |  |  |  |  |  | x |  |  |  |  |
| Coumarins | Scopoletin | 4.582 | 193.0493 | [M+H]^+^ | 2 | [±] |  |  | x | x | x |  |  |  | x |  |  |  |  |
| Flavonoids | Tectorigenin | 11.906 | 301.0694 | [M+H]^+^ | 2 | [±] |  |  | x |  |  |  |  |  |  | x |  |  |  |
| Coumarins | Tomasin | 11.954 | 387.1433 | [M+H]^+^ | 2 | [±] |  |  |  | x | x |  |  |  |  | x |  |  |  |
| Flavonoids | Apigenin-Hexoside | 11.639 | 431.0998 | [M-H]^-^ | 3 | [-] |  |  | x |  |  |  |  |  | x |  |  |  |  |
| Coumarins | Auraptenol | 6.488 | 261.1116 | [M+H]^+^ | 3 | [+] |  |  |  | x |  |  |  |  |  | x |  |  |  |
| Coumarins | Auraptenol | 10.014 | 261.1119 | [M+H]^+^ | 3 | [+] |  |  |  | x |  |  |  |  |  | x |  |  |  |
| Coumarins | Auraptenol | 11.024 | 261.1118 | [M+H]^+^ | 3 | [+] |  |  |  | x |  |  |  |  |  | x |  |  |  |
| Coumarins | Auraptenol | 8.524 | 243.1011 | [M-H_2_O+H]^+^ | 3 | [+] |  |  |  | x |  |  |  |  |  | x |  |  |  |
| Coumarins | Auraptenol | 9.58 | 243.1011 | [M-H_2_O+H]^+^ | 3 | [+] |  |  |  | x |  |  |  |  |  | x |  |  |  |
| Phenolic compounds | Caffeic acid derivate | 3.091 | 179.0363 | [M-H]^-^ | 3 | [-] |  |  |  | x |  |  | x |  |  |  |  |  |  |
| Phenolic compounds | Caffeic acid derivate | 5.186 | 179.0349 | [M-H]^-^ | 3 | [-] |  |  |  |  | x |  |  |  | x |  |  |  |  |
| Phenolic compounds | Caffeic acid derivate | 5.796 | 179.0363 | [M-H]^-^ | 3 | [-] |  |  |  | x |  |  | x |  |  |  |  |  |  |
| Phenolic compounds | Caffeic acid derivate | 6.556 | 179.0364 | [M-H]^-^ | 3 | [-] |  |  |  |  | x |  | x |  |  |  |  |  |  |
| Phenolic compounds | Caffeic Acid-Hexoside | 3.281 | 341.09 | [M-H]^-^ | 3 | [-] |  |  | x | x | x |  | x |  |  |  |  |  |  |
| Phenolic compounds | Caffeic Acid-Hexoside | 3.417 | 341.0905 | [M-H]^-^ | 3 | [-] |  |  |  |  | x |  |  | x |  |  |  |  |  |
| Phenolic compounds | Catechol | 2.572 | 109.0297 | [M-H]^-^ | 3 | [-] |  |  | x | x | x |  | x |  |  |  |  |  |  |
| Phenolic compounds | Catechol | 3.146 | 109.0293 | [M-H]^-^ | 3 | [-] |  |  | x |  |  |  | x |  |  |  |  |  |  |
| Coumarins | Columbianetin | 5.044 | 247.0957 | [M+H]^+^ | 3 | [+] |  |  |  |  | x |  |  |  |  | x |  |  |  |
| Coumarins | Columbianetin | 6.495 | 247.0961 | [M+H]^+^ | 3 | [+] |  |  |  | x | x |  |  |  |  | x |  |  |  |
| Coumarins | Columbianetin | 6.823 | 247.0962 | [M+H]^+^ | 3 | [+] |  |  |  | x | x |  |  |  |  | x |  |  |  |
| Coumarins | Columbianetin | 4.523 | 245.0828 | [M-H]^-^ | 3 | [-] |  |  |  |  | x |  |  |  | x |  |  |  |  |
| Coumarins | Columbianetin | 6.032 | 245.0837 | [M-H]^-^ | 3 | [-] |  |  | x | x | x |  |  |  |  | x |  |  |  |
| Coumarins | Columbianetin | 6.31 | 245.0826 | [M-H]^-^ | 3 | [-] |  |  | x | x | x |  |  |  |  | x |  |  |  |
| Coumarins | Columbianetin | 6.62 | 245.0816 | [M-H]^-^ | 3 | [-] |  |  | x | x | x |  |  |  |  | x |  |  |  |
| Coumarins | Columbianetin | 7.031 | 245.0817 | [M-H]^-^ | 3 | [-] |  |  | x |  |  |  |  |  |  | x |  |  |  |
| Coumarins | Columbianetin | 11.797 | 245.0817 | [M-H]^-^ | 3 | [-] |  |  |  | x |  |  |  |  |  | x |  |  |  |
| Coumarins | Coumarin-Hexoside | 4.068 | 447.1256 | [M+Na]^+^ | 3 | [+] |  |  |  | x |  |  |  |  | x |  |  |  |  |
| Coumarins | Coumarin-Hexoside | 4.231 | 447.1256 | [M+Na]^+^ | 3 | [+] |  |  |  | x |  |  |  |  | x |  |  |  |  |
| Phenolic compounds | Coumaroyl-Hexoside | 3.27 | 325.0922 | [M-H]^-^ | 3 | [-] |  |  | x |  |  |  | x |  | x |  |  |  |  |
| Coumarins | Dehydro-hydroxymellein | 5.558 | 191.0348 | [M-H]^-^ | 3 | [-] |  |  | x |  |  |  |  | x |  |  |  |  |  |
| Coumarins | Dehydro-hydroxymellein | 6.836 | 191.0363 | [M-H]^-^ | 3 | [-] |  |  | x |  |  |  |  |  |  | x |  |  |  |
| Flavonoids | Diosmetin-O-hexosyl-deoxyhexoside | 6.855 | 609.1801 | [M+H]^+^ | 3 | [+] |  |  | x | x |  |  |  |  | x | x |  |  |  |
| Flavonoids | Diosmetin-O-hexosyl-deoxyhexoside | 12.07 | 609.1801 | [M+H]^+^ | 3 | [+] |  |  | x |  |  |  |  |  |  | x |  |  |  |
| Flavonoids | Diosmetin-O-hexosyl-deoxyhexoside | 13.564 | 609.1801 | [M+H]^+^ | 3 | [+] |  |  | x |  |  |  |  |  |  |  | x |  |  |
| Flavonoids | Diosmetin-O-hexosyl-deoxyhexoside | 8.554 | 607.1699 | [M-H]^-^ | 3 | [-] |  |  |  |  | x |  |  |  |  | x |  |  |  |
| Coumarins | Esculetin | 3.697 | 177.0211 | [M-H]^-^ | 3 | [-] |  |  | x | x | x |  |  |  | x |  |  |  |  |
| Coumarins | Esculin | 3.462 | 339.0754 | [M-H]^-^ | 3 | [-] |  |  | x | x | x |  |  | x | x |  |  |  |  |
| Coumarins | Esculin | 9.464 | 339.0754 | [M-H]^-^ | 3 | [-] |  |  |  | x |  |  |  |  |  | x |  |  |  |
| Phenolic compounds | Ferulic acid derivate | 3.471 | 193.0603 | [M-H]^-^ | 3 | [-] |  |  |  | x |  |  | x |  |  |  |  |  |  |
| Coumarins | Fraxin | 3.571 | 369.0822 | [M-H]^-^ | 3 | [-] |  |  | x |  |  |  |  | x | x |  |  |  |  |
| Flavonoids | Genistein-Hexoside | 5.128 | 431.1001 | [M-H]^-^ | 3 | [-] |  |  |  |  | x |  |  | x |  |  |  |  |  |
| Phenolic compounds | Isoferulic acid | 5.115 | 193.0517 | [M-H]^-^ | 3 | [-] |  |  | x |  |  |  |  | x |  |  |  |  |  |
| Phenolic compounds | Isoferulic acid | 5.928 | 193.0517 | [M-H]^-^ | 3 | [-] |  |  | x |  |  |  |  | x |  |  |  |  |  |
| Phenolic compounds | Isoferulic acid | 7.521 | 193.052 | [M-H]^-^ | 3 | [-] |  |  |  | x |  |  |  |  |  | x |  |  |  |
| Coumarins | Isofraxidin | 3.911 | 221.0466 | [M-H]^-^ | 3 | [-] |  |  | x | x |  |  | x | x |  |  |  |  |  |
| Coumarins | Isoimperatorin | 12.037 | 271.0961 | [M+H]^+^ | 3 | [+] |  |  |  |  | x |  |  |  |  | x |  |  |  |
| Coumarins | Isoimperatorin | 12.102 | 269.0845 | [M-H]^-^ | 3 | [-] |  |  |  |  | x |  |  |  |  | x |  |  |  |
| Coumarins | Isopimpinellin | 11.099 | 247.0602 | [M+H]^+^ | 3 | [+] |  |  | x | x | x |  |  |  |  | x |  |  |  |
| Flavonoids | Isorhamnetin-O-Hexosyl-deoxyhexoside | 6.328 | 623.1632 | [M-H]^-^ | 3 | [-] |  |  | x |  |  |  |  |  | x |  |  |  |  |
| Phenolic compounds | Isorhapontigenin-Hexoside | 10.269 | 419.1339 | [M-H]^-^ | 3 | [-] |  |  | x |  |  |  |  |  | x |  |  |  |  |
| Flavonoids | Kaempferol-O-hexosyl-deoxyhexoside | 11.51 | 595.1647 | [M+H]^+^ | 3 | [+] |  |  | x |  |  |  |  |  |  | x |  |  |  |
| Flavonoids | Kaempferol-O-hexosyl-deoxyhexoside | 12.933 | 595.1647 | [M+H]^+^ | 3 | [+] |  |  | x |  |  |  |  |  |  |  | x |  |  |
| Flavonoids | Kaempferol-O-hexosyl-deoxyhexoside | 9.925 | 595.1647 | [M+H]^+^ | 3 | [+] |  |  | x |  |  |  |  |  |  | x |  |  |  |
| Flavonoids | Kaempferol-O-hexosyl-deoxyhexoside | 12.376 | 595.1647 | [M+H]^+^ | 3 | [+] |  |  | x |  |  |  |  |  |  | x |  |  |  |
| Flavonoids | Kaempferol-O-hexosyl-deoxyhexoside | 6.024 | 593.1536 | [M-H]^-^ | 3 | [-] |  |  | x |  |  |  |  |  | x |  |  |  |  |
| Flavonoids | Luteolin-Hexoside | 6.108 | 447.0923 | [M-H]^-^ | 3 | [-] |  |  | x |  |  |  |  |  | x |  |  |  |  |
| Flavonoids | Luteolin-Hexoside | 7.635 | 447.0943 | [M-H]^-^ | 3 | [-] |  |  | x |  |  |  |  |  | x |  |  |  |  |
| Flavonoids | Luteolin-Hexoside | 5.935 | 447.0923 | [M-H]^-^ | 3 | [-] |  |  | x |  |  |  |  |  | x |  |  |  |  |
| Coumarins | Meranzin | 6.495 | 301.1046 | [M+H]^+^ | 3 | [+] |  |  |  | x |  |  |  |  |  | x |  |  |  |
| Coumarins | Methylbutanoyl-angeloyl vaginidiol | 11.939 | 446.2163 | [M+NH_4_]^+^ | 3 | [+] |  |  | x | x |  |  |  |  |  | x |  |  |  |
| Coumarins | Methylbutanoyl-angeloyl vaginidiol | 12.337 | 446.2163 | [M+NH_4_]^+^ | 3 | [+] |  |  | x | x |  |  |  |  |  | x | x |  |  |
| Coumarins | Methylumbelliferone | 4.605 | 175.0416 | [M-H]^-^ | 3 | [-] |  |  |  | x |  |  |  |  | x |  |  |  |  |
| Coumarins | Oroselol | 4.245 | 227.0703 | [M-H_2_O+H]^+^ | 3 | [+] |  |  |  |  | x |  |  |  | x |  |  |  |  |
| Coumarins | Oroselol | 11.988 | 227.0703 | [M-H_2_O+H]^+^ | 3 | [+] |  |  |  | x |  |  |  |  |  | x |  |  |  |
| Coumarins | Oroselol | 12.342 | 227.0699 | [M-H_2_O+H]^+^ | 3 | [+] |  |  | x | x | x |  |  |  |  | x | x |  |  |
| Coumarins | Oroselol | 12.712 | 227.0705 | [M-H_2_O+H]^+^ | 3 | [+] |  |  | x | x |  |  |  |  |  |  | x |  |  |
| Coumarins | Osthenol | 11.525 | 231.1016 | [M+H]^+^ | 3 | [+] |  |  |  | x |  |  |  |  |  | x |  |  |  |
| Coumarins | Osthenol | 11.878 | 231.1016 | [M+H]^+^ | 3 | [+] |  |  |  | x |  |  |  |  |  | x |  |  |  |
| Coumarins | Osthol | 12.165 | 245.1167 | [M+H]^+^ | 3 | [+] |  |  | x | x |  |  |  |  |  |  | x |  |  |
| Coumarins | Oxypeucedanin hydrate | 7.178 | 305.1017 | [M+H]^+^ | 3 | [+] |  |  |  | x |  |  |  |  |  | x |  |  |  |
| Coumarins | Phellopterin | 11.26 | 323.0889 | [M+Na]^+^ | 3 | [+] |  |  |  |  | x |  |  |  |  | x |  |  |  |
| Coumarins | Phellopterin | 12.295 | 323.0894 | [M+Na]^+^ | 3 | [+] |  |  |  |  | x |  |  |  |  | x |  |  |  |
| Coumarins | Phellopterin | 12.468 | 323.0892 | [M+Na]^+^ | 3 | [+] |  |  |  |  | x |  |  |  |  |  | x |  |  |
| Coumarins | Psoralen / Angelicin | 4.069 | 187.0386 | [M+H]^+^ | 3 | [+] |  |  | x | x |  |  |  |  | x |  |  |  |  |
| Coumarins | Psoralen / Angelicin | 4.229 | 187.0387 | [M+H]^+^ | 3 | [+] |  |  |  | x | x |  |  |  | x |  |  |  |  |
| Coumarins | Psoralen / Angelicin | 5.743 | 187.0389 | [M+H]^+^ | 3 | [+] |  |  | x |  |  |  |  |  |  | x |  |  |  |
| Coumarins | Psoralen / Angelicin | 6.727 | 187.0388 | [M+H]^+^ | 3 | [+] |  |  |  | x | x |  |  |  |  | x |  |  |  |
| Coumarins | Psoralen / Angelicin | 7.414 | 187.0388 | [M+H]^+^ | 3 | [+] |  |  | x | x | x |  |  |  |  | x |  |  |  |
| Coumarins | Psoralen / Angelicin | 11.349 | 187.0387 | [M+H]^+^ | 3 | [+] |  |  | x | x |  |  |  |  |  | x |  |  |  |
| Coumarins | Psoralen / Angelicin | 11.801 | 187.0388 | [M+H]^+^ | 3 | [+] |  |  | x | x | x |  |  |  |  | x |  |  |  |
| Coumarins | Psoralen / Angelicin | 4.898 | 187.0385 | [M+H]^+^ | 3 | [+] |  |  | x | x | x |  |  |  |  | x |  |  |  |
| Phenolic compounds | Rosmarinic acid | 3.54 | 163.0389 | [M+H-C_9_H_10_O_5_]^+^ | 3 | [+] |  |  | x | x | x |  | x |  |  |  |  |  |  |
| Phenolic compounds | Rosmarinic acid | 4.21 | 163.0386 | [M+H-C_9_H_10_O_5_]^+^ | 3 | [+] |  |  |  | x |  |  |  |  | x |  |  |  |  |
| Phenolic compounds | Rosmarinic acid | 4.87 | 163.0386 | [M+H-C_9_H_10_O_5_]^+^ | 3 | [+] |  |  | x |  |  |  |  | x |  |  |  |  |  |
| Flavonoids | Schaftoside | 4.206 | 563.1412 | [M-H]^-^ | 3 | [-] |  |  | x |  |  |  |  |  | x |  |  |  |  |
| Coumarins | Scopoletin | 6.727 | 193.0492 | [M+H]^+^ | 3 | [+] |  |  | x |  |  |  |  |  |  | x |  |  |  |
| Coumarins | Scopoletin | 13.631 | 193.0491 | [M+H]^+^ | 3 | [+] |  |  | x |  |  |  |  |  |  |  |  | x |  |
| Phenolic compounds | Secoisolariciresinol | 4.251 | 361.1655 | [M-H]^-^ | 3 | [-] |  |  |  | x |  |  |  |  | x |  |  |  |  |
| Phenolic compounds | Secoisolariciresinol | 5.67 | 361.1687 | [M-H]^-^ | 3 | [-] |  |  |  | x | x |  |  |  | x |  |  |  |  |
| Coumarins | Tomasin | 12.53 | 387.1433 | [M+H]^+^ | 3 | [+] |  |  |  | x |  |  |  |  |  | x |  |  |  |
| Coumarins | Tomasin | 11.792 | 369.1333 | [M-H_2_O+H]^+^ | 3 | [+] |  |  | x | x |  |  |  |  |  | x |  |  |  |
| Coumarins | Umbelliferone | 8.116 | 161.0259 | [M-H]^-^ | 3 | [-] |  |  |  |  | x |  |  |  | x |  |  |  |  |
| Coumarins | Umbelliferone | 11.547 | 161.0258 | [M-H]^-^ | 3 | [-] |  |  | x |  |  |  |  |  | x |  |  |  |  |
| Coumarins | Umbelliferone sulfate | 3.487 | 240.9829 | [M-H]^-^ | 3 | [-] |  |  |  |  | x |  |  | x |  |  |  |  |  |
| Coumarins | Xanthotoxin | 8.028 | 217.0499 | [M+H]^+^ | 3 | [+] |  |  | x | x | x |  |  |  |  | x |  |  |  |
| Coumarins | Xanthyletin | 4.209 | 227.0714 | [M-H]^-^ | 3 | [-] |  |  |  |  | x |  |  |  | x |  |  |  |  |
| Coumarins | Xanthyletin | 6.063 | 227.0714 | [M-H]^-^ | 3 | [-] |  |  |  |  | x |  |  |  |  | x |  |  |  |
| Coumarins | Xanthyletin | 6.306 | 227.0714 | [M-H]^-^ | 3 | [-] |  |  |  |  | x |  |  |  |  | x |  |  |  |
| Coumarins | Xanthyletin | 6.592 | 227.0733 | [M-H]^-^ | 3 | [-] |  |  |  | x | x |  |  |  |  | x |  |  |  |
| Coumarins | Xanthyletin | 12.275 | 227.0733 | [M-H]^-^ | 3 | [-] |  |  |  | x | x |  |  |  |  | x | x |  |  |

**Tab. S6.** Overview of all compounds found in *A. eupatoria*.

|  |  |  |  |  |  |  | Plant Part | | | | | Fraction | | | | | | | |
| --- | --- | --- | --- | --- | --- | --- | --- | --- | --- | --- | --- | --- | --- | --- | --- | --- | --- | --- | --- |
| Ontology | Compound Name | RT | Precursor mass | Adduct | Level | Polarity | Flowers | Berries | Leaves | Roots | Bark | 1 | 2 | 3 | 4 | 5 | 6 | 7 | 8 |
| Phenolic compounds | 4-Coumaric acid | 4.192 | 163.0404 | [M-H]^-^ | 1 | [±] |  |  | x |  |  |  | x | x |  |  |  |  |  |
| Other nitrogen compounds | Adenosine | 1.12 | 268.1034 | [M+H]^+^ | 1 | [±] |  |  | x |  |  |  | x | x | x |  |  |  |  |
| Flavonoids | Apigenin | 11.863 | 271.0602 | [M+H]^+^ | 1 | [±] |  |  | x |  |  | x |  |  |  | x |  |  |  |
| Phenolic compounds | Caffeic acid | 3.602 | 179.0363 | [M-H]^-^ | 1 | [±] |  |  | x |  |  | x | x |  |  |  |  |  |  |
| Flavonoids | Catechin | 3.345 | 291.0861 | [M+H]^+^ | 1 | [±] |  |  | x |  |  |  | x |  | x |  |  |  |  |
| Phenolic compounds | Chlorogenic acid | 3.469 | 355.1019 | [M+H]^+^ | 1 | [±] |  |  | x |  |  | x | x |  | x |  |  |  |  |
| Flavonoids | Isoquercetin | 5.239 | 465.1022 | [M+H]^+^ | 1 | [±] |  |  | x |  |  |  |  |  | x |  |  |  |  |
| Flavonoids | Kaempferol | 11.818 | 287.0548 | [M+H]^+^ | 1 | [±] |  |  | x |  |  |  |  |  |  | x |  |  |  |
| Other acid compounds | Kynurenic acid | 3.827 | 190.0496 | [M+H]^+^ | 1 | [±] |  |  | x |  |  | x | x |  |  |  |  |  |  |
| Flavonoids | Luteolin | 10.881 | 287.0547 | [M+H]^+^ | 1 | [±] |  |  | x |  |  |  |  |  |  | x |  |  |  |
| Flavonoids | Naringenin | 9.865 | 273.0753 | [M+H]^+^ | 1 | [±] |  |  | x |  |  |  |  |  |  | x |  |  |  |
| Other acid compounds | Phosphoric acid | 0.664 | 96.96104 | [M-H]^-^ | 1 | [±] |  |  | x |  |  | x |  |  |  |  |  |  |  |
| Flavonoids | Rutin | 5.109 | 633.1417 | [M+Na]^+^ | 1 | [±] |  |  | x |  |  | x | x | x | x |  |  |  |  |
| Amino compounds | Tryptophan | 3.321 | 188.0703 | [M-H_2_O+H]^+^ | 1 | [±] |  |  | x |  |  | x | x | x | x |  |  |  |  |
| Lipids | y-Linolenic acid | 13.059 | 277.2203 | [M-H]^-^ | 1 | [±] |  |  | x |  |  |  |  |  |  |  | x |  |  |
| Others | Aloeemodin | 6.062 | 271.0596 | [M+H]^+^ | 2 | [±] |  |  | x |  |  |  | x | x |  |  |  |  |  |
| Flavonoids | Apigenin-Glucuronide | 6.716 | 447.092 | [M+H]^+^ | 2 | [±] |  |  | x |  |  |  | x |  |  |  |  |  |  |
| Flavonoids | Apigenin-Hexoside | 4.885 | 433.1123 | [M+H]^+^ | 2 | [±] |  |  | x |  |  |  |  |  | x |  |  |  |  |
| Flavonoids | Apigenin-Hexoside | 4.696 | 433.1128 | [M+H]^+^ | 2 | [±] |  |  | x |  |  |  |  |  | x |  |  |  |  |
| Flavonoids | Baicalein-Glucuronide | 5.987 | 447.0919 | [M+H]^+^ | 2 | [±] |  |  | x |  |  |  | x |  |  |  |  |  |  |
| Carbohydrates | beta-Gentiobiose | 0.632 | 360.1496 | [M+NH_4_]^+^ | 2 | [±] |  |  | x |  |  | x |  |  |  |  |  |  |  |
| Flavonoids | Catechin derivate | 3.615 | 291.0858 | [M+H]^+^ | 2 | [±] |  |  | x |  |  |  | x |  | x |  |  |  |  |
| Flavonoids | Diosmetin-O-hexosyl-deoxyhexoside | 6.939 | 609.1805 | [M+H]^+^ | 2 | [±] |  |  | x |  |  |  |  |  | x |  |  |  |  |
| Phenolic compounds | Divaric acid | 6.185 | 197.0808 | [M+H]^+^ | 2 | [±] |  |  | x |  |  |  |  | x |  |  |  |  |  |
| Phenolic compounds | Divaric acid | 12.091 | 197.0808 | [M+H]^+^ | 2 | [±] |  |  | x |  |  |  |  |  |  | x |  |  |  |
| Phenolic compounds | Homovanillic Acid | 4.919 | 181.0516 | [M-H]^-^ | 2 | [±] |  |  | x |  |  |  |  |  |  | x |  |  |  |
| Phenolic compounds | Homovanillic Acid | 9.312 | 181.0516 | [M-H]^-^ | 2 | [±] |  |  | x |  |  |  |  |  |  | x |  |  |  |
| Coumarins | Isofraxidin | 6.614 | 223.0597 | [M+H]^+^ | 2 | [±] |  |  | x |  |  |  |  |  |  | x |  |  |  |
| Flavonoids | Kaempferol-Glucuronide | 5.093 | 463.0867 | [M+H]^+^ | 2 | [±] |  |  | x |  |  |  | x |  |  |  |  |  |  |
| Flavonoids | Kaempferol-Glucuronide | 5.57 | 463.0868 | [M+H]^+^ | 2 | [±] |  |  | x |  |  |  | x |  |  |  |  |  |  |
| Flavonoids | Kaempferol-p-coumaroylhexoside | 10.635 | 595.1436 | [M+H]^+^ | 2 | [±] |  |  | x |  |  |  |  |  |  | x |  |  |  |
| Phenolic compounds | Neochlorogenic acid | 3.085 | 355.1019 | [M+H]^+^ | 2 | [±] |  |  | x |  |  | x |  |  |  |  |  |  |  |
| Phenolic compounds | Quercetin-deoxyhexoside | 6.115 | 449.1069 | [M+H]^+^ | 2 | [±] |  |  | x |  |  |  |  |  | x | x |  |  |  |
| Flavonoids | Acacetin-O-hexosyl-deoxyhexoside | 9.711 | 593.1859 | [M+H]^+^ | 3 | [+] |  |  | x |  |  |  |  |  |  | x |  |  |  |
| Amino compounds | Agmatine | 0.593 | 131.1288 | [M+H]^+^ | 3 | [+] |  |  | x |  |  | x | x |  |  |  |  |  |  |
| Flavonoids | Apigenin | 6.531 | 269.0478 | [M-H]^-^ | 3 | [-] |  |  | x |  |  |  | x |  |  |  |  |  |  |
| Flavonoids | Apigenin-Hexoside | 4.434 | 431.1026 | [M-H]^-^ | 3 | [-] |  |  | x |  |  |  |  |  | x |  |  |  |  |
| Amino compounds | Arginine | 0.612 | 175.1185 | [M+H]^+^ | 3 | [+] |  |  | x |  |  | x | x | x |  |  |  |  |  |
| Other acid compounds | Benzoic acid | 3.602 | 121.0309 | [M-H]^-^ | 3 | [-] |  |  | x |  |  |  |  |  | x |  |  |  |  |
| Carbohydrates | beta-Gentiobiose | 3.618 | 360.1496 | [M+NH_4_]^+^ | 3 | [+] |  |  | x |  |  | x |  |  |  |  |  |  |  |
| Amino compounds | Betaine | 0.699 | 118.0862 | [M+H]^+^ | 3 | [+] |  |  | x |  |  | x | x |  |  |  |  |  |  |
| Amino compounds | Betaine | 13.219 | 100.0756 | [M-H_2_O+H]^+^ | 3 | [+] |  |  | x |  |  |  |  |  |  |  |  | x |  |
| Amino compounds | Carnitine | 0.697 | 162.1122 | [M+H]^+^ | 3 | [+] |  |  | x |  |  | x | x |  |  |  |  |  |  |
| Flavonoids | Catechin derivate | 0.751 | 289.0742 | [M-H]^-^ | 3 | [-] |  |  | x |  |  | x |  |  |  |  |  |  |  |
| Phenolic compounds | Catechol | 2.601 | 109.0307 | [M-H]^-^ | 3 | [-] |  |  | x |  |  | x | x |  |  |  |  |  |  |
| Phenolic compounds | Catechol | 4.268 | 109.0307 | [M-H]^-^ | 3 | [-] |  |  | x |  |  |  | x |  |  |  |  |  |  |
| Amino compounds | Choline | 0.669 | 104.1068 | [M]^+^ | 3 | [+] |  |  | x |  |  | x | x | x |  |  |  |  |  |
| Terpenes & Terpenoids | Corosolic acid | 12.693 | 471.3527 | [M-H]^-^ | 3 | [-] |  |  | x |  |  |  |  |  |  |  | x | x |  |
| Phenolic compounds | Coumaric acid | 11.052 | 147.044 | [M-H_2_O+H]^+^ | 3 | [+] |  |  | x |  |  |  |  |  |  | x |  |  |  |
| Phenolic compounds | Coumaric acid | 11.85 | 147.0441 | [M-H_2_O+H]^+^ | 3 | [+] |  |  | x |  |  |  |  |  |  | x |  |  |  |
| Phenolic compounds | Coumaroylquinic acid | 3.354 | 337.0962 | [M-H]^-^ | 3 | [-] |  |  | x |  |  | x | x |  |  |  |  |  |  |
| Phenolic compounds | Coumaroylquinic acid | 3.779 | 337.0962 | [M-H]^-^ | 3 | [-] |  |  | x |  |  | x | x |  |  |  |  |  |  |
| Coumarins | Dehydrohydroxymellein | 6.823 | 191.0367 | [M-H]^-^ | 3 | [-] |  |  | x |  |  |  |  |  |  | x |  |  |  |
| Flavonoids | Dihydrokaempferol | 5.262 | 287.059 | [M-H]^-^ | 3 | [-] |  |  | x |  |  |  |  |  | x |  |  |  |  |
| Flavonoids | Dihydroquercetin-Hexoside | 4.124 | 465.1083 | [M-H]^-^ | 3 | [-] |  |  | x |  |  |  |  |  | x |  |  |  |  |
| Phenolic compounds | Dihydroxybenzoic acid | 4.271 | 153.0199 | [M-H]^-^ | 3 | [-] |  |  | x |  |  |  | x |  |  |  |  |  |  |
| Phenolic compounds | Dihydroxybenzoic acid | 2.597 | 153.021 | [M-H]^-^ | 3 | [-] |  |  | x |  |  |  | x |  |  |  |  |  |  |
| Flavonoids | Dihydroxyflavone | 9.035 | 253.0526 | [M-H]^-^ | 3 | [-] |  |  | x |  |  |  |  |  |  | x |  |  |  |
| Phenolic compounds | Dimethoxycinnamic acid | 11.819 | 209.0807 | [M+H]^+^ | 3 | [+] |  |  | x |  |  |  |  |  |  | x | x |  |  |
| Flavonoids | Diosmetin | 11.815 | 299.0577 | [M-H]^-^ | 3 | [-] |  |  | x |  |  | x |  |  |  | x |  |  |  |
| Amino compounds | Dopamine | 0.916 | 137.0593 | [M+H]^+^ | 3 | [+] |  |  | x |  |  | x | x |  |  |  |  |  |  |
| Phenolic compounds | Elmycin A | 7.348 | 367.1147 | [M+Na]^+^ | 3 | [+] |  |  | x |  |  |  |  |  |  | x |  |  |  |
| Flavonoids | Eriodictyol | 8.195 | 287.0565 | [M-H]^-^ | 3 | [-] |  |  | x |  |  |  |  |  |  | x |  |  |  |
| Carbohydrates | Erythrose | 0.619 | 101.0245 | [M-H_2_O-H]^-^ | 3 | [-] |  |  | x |  |  | x | x |  |  |  |  |  |  |
| Coumarins | Esculetin | 3.69 | 177.0208 | [M-H]^-^ | 3 | [-] |  |  | x |  |  |  |  |  | x |  |  |  |  |
| Phenolic compounds | Ferulic acid | 3.801 | 177.0541 | [M-H_2_O+H]^+^ | 3 | [+] |  |  | x |  |  |  |  |  |  | x | x |  |  |
| Phenolic compounds | Ferulic acid | 6.66 | 177.0541 | [M-H_2_O+H]^+^ | 3 | [+] |  |  | x |  |  |  |  |  |  |  | x |  |  |
| Phenolic compounds | Feruloylquinic acid | 3.541 | 367.104 | [M-H]^-^ | 3 | [-] |  |  | x |  |  | x |  |  |  |  |  |  |  |
| Phenolic compounds | Feruloylquinic acid | 4.003 | 367.104 | [M-H]^-^ | 3 | [-] |  |  | x |  |  | x |  |  |  |  |  |  |  |
| Phenolic compounds | Flavaspidic acid | 15.072 | 417.1602 | [M-H]^-^ | 3 | [-] |  |  | x |  |  |  |  |  |  |  |  |  |  |
| Phenolic compounds | Gentisyl alcohol | 3.303 | 141.0544 | [M+H]^+^ | 3 | [+] |  |  | x |  |  |  |  |  | x |  |  |  |  |
| Other acid compounds | Gluconic acid | 0.619 | 195.0527 | [M-H]^-^ | 3 | [-] |  |  | x |  |  | x |  |  |  |  |  |  |  |
| Terpenes & Terpenoids | glucopyranosyloxy-hydroxytrimethyl-cyclohexylidene-Butenone | 4.156 | 411.1984 | [M+Na]^+^ | 3 | [+] |  |  | x |  |  |  |  |  | x |  |  |  |  |
| Terpenes & Terpenoids | glucopyranosyloxy-hydroxytrimethyl-cyclohexylidene-Butenone | 3.686 | 409.1826 | [M+Na]^+^ | 3 | [+] |  |  | x |  |  |  |  |  | x |  |  |  |  |
| Carbohydrates | Glucose | 0.659 | 179.0573 | [M-H]^-^ | 3 | [-] |  |  | x |  |  | x |  |  |  |  |  |  |  |
| Other acid compounds | Glutaric acid | 1.564 | 131.0353 | [M-H]^-^ | 3 | [-] |  |  | x |  |  | x | x |  |  |  |  |  |  |
| Carbohydrates | Glyceraldehyde | 0.607 | 89.02446 | [M-H]^-^ | 3 | [-] |  |  | x |  |  |  | x |  |  |  |  |  |  |
| Phenolic compounds | Homovanillic Acid | 14.564 | 181.0516 | [M-H]^-^ | 3 | [-] |  |  | x |  |  |  |  |  |  | x |  |  |  |
| Lipids | Hydroxy-octadecadienoic acid | 12.396 | 295.231 | [M-H]^-^ | 3 | [-] |  |  | x |  |  |  |  |  |  | x |  |  |  |
| Lipids | Hydroxyoctadecatrienic acid | 12.253 | 293.2128 | [M-H]^-^ | 3 | [-] |  |  | x |  |  |  |  |  |  | x |  |  |  |
| Phenolic compounds | Hydroxyphenylacetic acid | 5.483 | 151.041 | [M-H]^-^ | 3 | [-] |  |  | x |  |  |  | x |  |  |  |  |  |  |
| Other nitrogen compounds | Indole-3-carboxyaldehyde | 3.81 | 144.0459 | [M-H]^-^ | 3 | [-] |  |  | x |  |  | x | x |  |  |  |  |  |  |
| Flavonoids | Kaempferol derivate | 6.119 | 287.0548 | [M+H]^+^ | 3 | [+] |  |  | x |  |  |  |  |  | x | x |  |  |  |
| Flavonoids | Kaempferol-Hexoside | 5.602 | 449.1077 | [M+H]^+^ | 3 | [+] |  |  | x |  |  |  |  |  | x |  |  |  |  |
| Flavonoids | Kaempferol-O-hexosyl-deoxyhexoside | 3.725 | 593.1542 | [M-H]^-^ | 3 | [-] |  |  | x |  |  |  | x |  | x |  |  |  |  |
| Flavonoids | Kaempferol-O-hexosyl-deoxyhexoside | 4.046 | 593.1542 | [M-H]^-^ | 3 | [-] |  |  | x |  |  |  | x |  | x |  |  |  |  |
| Flavonoids | Kaempferol-p-coumaroylhexoside | 11.261 | 593.1368 | [M-H]^-^ | 3 | [-] |  |  | x |  |  |  |  |  |  | x |  |  |  |
| Others | Loliolide | 4.188 | 197.1171 | [M+H]^+^ | 3 | [+] |  |  | x |  |  |  |  |  | x |  |  |  |  |
| Terpenes & Terpenoids | Loliolide-Hexoside | 4.657 | 397.2187 | [M+Na]^+^ | 3 | [+] |  |  | x |  |  |  |  |  | x | x |  |  |  |
| Alkaloids | Lupanine | 3.894 | 249.1959 | [M+H]^+^ | 3 | [+] |  |  | x |  |  |  |  |  |  |  | x | x | x |
| Flavonoids | Luteolin-Hexoside | 7.618 | 447.095 | [M-H]^-^ | 3 | [-] |  |  | x |  |  |  |  |  |  | x |  |  |  |
| Flavonoids | Luteolin-Hexoside | 4.307 | 447.0971 | [M-H]^-^ | 3 | [-] |  |  | x |  |  |  | x |  |  |  |  |  |  |
| Flavonoids | Luteolin-O-dihexoside | 11.182 | 595.144 | [M+H]^+^ | 3 | [+] |  |  | x |  |  |  |  |  |  | x |  |  |  |
| Flavonoids | Luteolin-O-dihexoside | 4.633 | 595.1358 | [M-H]^-^ | 3 | [-] |  |  | x |  |  |  |  |  | x |  |  |  |  |
| Lipids | Lyso‑PG 16:0 | 13.319 | 483.2727 | [M-H]^-^ | 3 | [-] |  |  | x |  |  |  |  |  |  | x |  |  |  |
| Terpenes & Terpenoids | Methyloxooctyl-furanone | 4.133 | 247.1304 | [M+Na]^+^ | 3 | [+] |  |  | x |  |  |  |  |  | x | x |  |  |  |
| Flavonoids | Naringenin derivate | 9.06 | 271.0639 | [M-H]^-^ | 3 | [-] |  |  | x |  |  |  |  |  |  | x |  |  |  |
| Flavonoids | Naringenin-Hexoside | 4.065 | 433.1144 | [M-H]^-^ | 3 | [-] |  |  | x |  |  |  | x |  | x |  |  |  |  |
| Flavonoids | Naringenin-Hexoside | 4.212 | 433.1144 | [M-H]^-^ | 3 | [-] |  |  | x |  |  |  |  |  | x |  |  |  |  |
| Flavonoids | Naringenin-Hexoside | 4.436 | 433.1144 | [M-H]^-^ | 3 | [-] |  |  | x |  |  |  |  |  | x |  |  |  |  |
| Flavonoids | Naringenin-Hexoside | 6.703 | 433.1144 | [M-H]^-^ | 3 | [-] |  |  | x |  |  |  |  |  | x |  |  |  |  |
| Flavonoids | Naringenin-Hexoside | 4.701 | 433.1144 | [M-H]^-^ | 3 | [-] |  |  | x |  |  |  |  |  | x |  |  |  |  |
| Flavonoids | Naringenin-Hexoside | 4.85 | 433.1144 | [M-H]^-^ | 3 | [-] |  |  | x |  |  |  |  |  | x |  |  |  |  |
| Flavonoids | Naringenin-Hexoside | 5.021 | 433.1144 | [M-H]^-^ | 3 | [-] |  |  | x |  |  |  |  |  | x |  |  |  |  |
| Flavonoids | Naringenin-Hexoside | 5.322 | 433.1144 | [M-H]^-^ | 3 | [-] |  |  | x |  |  |  |  |  | x |  |  |  |  |
| Flavonoids | Naringenin-Hexoside | 6.364 | 433.1144 | [M-H]^-^ | 3 | [-] |  |  | x |  |  |  |  |  | x |  |  |  |  |
| Flavonoids | Naringenin-Hexoside | 8.024 | 433.1144 | [M-H]^-^ | 3 | [-] |  |  | x |  |  |  |  |  | x | x |  |  |  |
| Phenolic compounds | Neochlorogenic acid | 3.977 | 355.1019 | [M+H]^+^ | 3 | [+] |  |  | x |  |  |  |  |  | x |  |  |  |  |
| Terpenes & Terpenoids | Oleanolic acid | 13.244 | 455.3572 | [M-H]^-^ | 3 | [-] |  |  | x |  |  |  |  |  |  |  |  | x |  |
| Other acid compounds | Olivetolcarboxylic acid | 12.923 | 223.0996 | [M-H]^-^ | 3 | [-] |  |  | x |  |  |  |  |  | x | x |  |  |  |
| Other acid compounds | Olivetolcarboxylic acid | 13.302 | 223.0996 | [M-H]^-^ | 3 | [-] |  |  | x |  |  |  |  |  |  | x |  |  |  |
| Phenolic compounds | Orcinolcarboxylic acid | 14.74 | 181.0508 | [M-H]^-^ | 3 | [-] |  |  | x |  |  |  |  |  |  | x |  |  |  |
| Phenolic compounds | Orsellinic acid | 3.45 | 167.0365 | [M-H]^-^ | 3 | [-] |  |  | x |  |  | x |  |  |  |  |  |  |  |
| Phenolic compounds | Orsellinic acid | 4.336 | 167.0365 | [M-H]^-^ | 3 | [-] |  |  | x |  |  |  | x |  |  |  |  |  |  |
| Others | Pantothenate | 1.943 | 218.1038 | [M-H]^-^ | 3 | [-] |  |  | x |  |  | x |  |  |  |  |  |  |  |
| Carbohydrates | Perseitol | 0.608 | 211.0827 | [M-H]^-^ | 3 | [-] |  |  | x |  |  | x | x |  |  |  |  |  |  |
| Others | Phenylacetaldehyde | 1.179 | 121.0646 | [M+H]^+^ | 3 | [+] |  |  | x |  |  | x | x | x | x | x |  |  |  |
| Other nitrogen compounds | Phenylethanolamine | 1.742 | 120.0805 | [M-H_2_O+H]^+^ | 3 | [+] |  |  | x |  |  | x | x | x |  |  |  |  |  |
| Flavonoids | Phloretin | 11.739 | 273.0797 | [M-H]^-^ | 3 | [-] |  |  | x |  |  |  |  |  |  | x |  |  |  |
| Flavonoids | Phloretin-Hexoside | 5.131 | 435.1333 | [M-H]^-^ | 3 | [-] |  |  | x |  |  |  |  |  | x |  |  |  |  |
| Flavonoids | Phloretin-Hexoside | 6.802 | 435.1333 | [M-H]^-^ | 3 | [-] |  |  | x |  |  |  |  |  | x |  |  |  |  |
| Flavonoids | Plantaginin | 7.466 | 449.1074 | [M+H]^+^ | 3 | [+] |  |  | x |  |  |  |  |  |  | x |  |  |  |
| Flavonoids | Procyanidin B | 3.299 | 577.1411 | [M-H]^-^ | 3 | [-] |  |  | x |  |  |  | x |  | x |  |  |  |  |
| Flavonoids | Procyanidin B | 3.544 | 577.1411 | [M-H]^-^ | 3 | [-] |  |  | x |  |  |  | x |  | x |  |  |  |  |
| Flavonoids | Procyanidin B | 3.848 | 577.1411 | [M-H]^-^ | 3 | [-] |  |  | x |  |  |  | x |  | x |  |  |  |  |
| Phenolic compounds | Protocatechuic aldehyde | 3.174 | 137.026 | [M-H]^-^ | 3 | [-] |  |  | x |  |  |  |  | x | x |  |  |  |  |
| Others | Pyridoxine | 0.969 | 170.0808 | [M+H]^+^ | 3 | [+] |  |  | x |  |  |  | x | x |  |  |  |  |  |
| Flavonoids | Quercetin | 9.182 | 303.0498 | [M+H]^+^ | 3 | [+] |  |  | x |  |  |  |  |  |  | x |  |  |  |
| Flavonoids | Quercetin-Acetylhexoside | 6.536 | 505.0988 | [M-H]^-^ | 3 | [-] |  |  | x |  |  |  |  |  | x |  |  |  |  |
| Flavonoids | Quercetin-Hexoside | 4.803 | 465.1021 | [M+H]^+^ | 3 | [+] |  |  | x |  |  |  |  |  | x |  |  |  |  |
| Flavonoids | Quercetin-Malonylhexoside | 5.969 | 549.0887 | [M-H]^-^ | 3 | [-] |  |  | x |  |  |  | x |  | x |  |  |  |  |
| Flavonoids | Quercetin-Malonylhexoside | 5.667 | 551.1026 | [M+H]^+^ | 3 | [+] |  |  | x |  |  |  | x |  | x |  |  |  |  |
| Flavonoids | Quercetin-O-hexosyl-deoxyhexoside | 3.735 | 609.1516 | [M-H]^-^ | 3 | [-] |  |  | x |  |  |  | x |  |  |  |  |  |  |
| Flavonoids | Quercetin-O-hexosyl-deoxyhexoside | 4.611 | 609.1499 | [M-H]^-^ | 3 | [-] |  |  | x |  |  |  |  |  | x |  |  |  |  |
| Flavonoids | Quercetin-O-hexosyl-pentoside | 3.899 | 595.1333 | [M-H]^-^ | 3 | [-] |  |  | x |  |  |  |  |  | x |  |  |  |  |
| Other acid compounds | Quinic acid | 0.677 | 191.058 | [M-H]^-^ | 3 | [-] |  |  | x |  |  | x |  |  |  |  |  |  |  |
| Phenolic compounds | Resveratrol | 9.047 | 227.0732 | [M-H]^-^ | 3 | [-] |  |  | x |  |  |  |  |  |  | x |  |  |  |
| Alkaloids | Salsolinol | 1.188 | 180.1014 | [M+H]^+^ | 3 | [+] |  |  | x |  |  | x | x | x | x | x |  |  |  |
| Carbohydrates | Sorbitol | 0.61 | 181.0739 | [M-H]^-^ | 3 | [-] |  |  | x |  |  | x | x |  |  |  |  |  |  |
| Flavonoids | Taxifolin-Hexoside | 5.069 | 465.1083 | [M-H]^-^ | 3 | [-] |  |  | x |  |  |  |  |  | x |  |  |  |  |
| Flavonoids | Taxifolin-Hexoside | 5.209 | 465.1083 | [M-H]^-^ | 3 | [-] |  |  | x |  |  |  |  |  | x |  |  |  |  |
| Flavonoids | Taxifolin-Hexoside | 3.838 | 465.1083 | [M-H]^-^ | 3 | [-] |  |  | x |  |  |  | x |  | x |  |  |  |  |
| Phenolic compounds | Trihydroxybenzoic acid | 3.399 | 169.0159 | [M-H]^-^ | 3 | [-] |  |  | x |  |  | x |  |  |  |  |  |  |  |
| Lipids | Trihydroxyoctadecenoic acid | 11.707 | 329.2367 | [M-H]^-^ | 3 | [-] |  |  | x |  |  |  |  |  |  | x |  |  |  |

**Tab. S7.** Overview of all compounds found in *S. ebulus*.

|  |  |  |  |  |  |  | Plant Part | | | | | Fraction | | | | | | | |
| --- | --- | --- | --- | --- | --- | --- | --- | --- | --- | --- | --- | --- | --- | --- | --- | --- | --- | --- | --- |
| Ontology | Compound Name | RT | Precursor mass | Adduct | Level | Polarity | Flowers | Berries | Leaves | Roots | Bark | 1 | 2 | 3 | 4 | 5 | 6 | 7 | 8 |
| Phenolic compounds | 2,5-Dihydroxybenzoic acid | 3.247 | 153.0199 | [M-H]^-^ | 1 | [±] |  |  |  | x |  |  | x |  |  |  |  |  |  |
| Other nitrogen compounds | Adenosine | 1.163 | 268.1038 | [M+H]^+^ | 1 | [±] |  |  |  | x |  |  | x | x | x |  |  |  |  |
| Amino compounds | Asparagine | 0.644 | 133.0606 | [M+H]^+^ | 1 | [±] |  |  |  | x |  | x | x |  |  |  |  |  |  |
| Coumarins | Bergapten | 11.566 | 217.0491 | [M+H]^+^ | 1 | [±] |  | x |  |  |  |  |  |  |  | x |  |  |  |
| Phenolic compounds | Caffeic acid | 3.569 | 163.0386 | [M-H_2_O+H]^+^ | 1 | [±] |  | x |  |  |  |  | x |  |  |  |  |  |  |
| Flavonoids | Catechin | 3.34 | 289.0716 | [M-H]^-^ | 1 | [±] |  |  |  | x |  |  | x | x | x |  |  |  |  |
| Phenolic compounds | Chlorogenic acid | 3.533 | 355.1013 | [M+H]^+^ | 1 | [±] |  |  |  | x |  |  | x |  |  |  |  |  |  |
| Other acid compounds | Citric acid | 0.696 | 191.0214 | [M-H]^-^ | 1 | [±] |  |  |  | x |  | x |  |  |  |  |  |  |  |
| Phenolic compounds | Ferulic acid | 4.458 | 193.0519 | [M-H]^-^ | 1 | [±] |  |  |  | x |  |  |  | x |  |  |  |  |  |
| Other acid compounds | Fumaric acid | 1.026 | 115.0036 | [M-H]^-^ | 1 | [±] |  | x |  | x |  | x | x |  |  |  |  |  |  |
| Amino compounds | Leucine | 1.068 | 130.088 | [M-H]^-^ | 1 | [±] |  | x |  | x |  |  | x |  |  |  |  |  |  |
| Flavonoids | Naringenin | 10.053 | 271.0626 | [M-H]^-^ | 1 | [±] |  | x |  | x |  |  |  |  |  | x |  |  |  |
| Other acid compounds | Nicotinic acid | 0.872 | 124.0391 | [M+H]^+^ | 1 | [±] |  | x |  | x |  |  | x |  |  |  |  |  |  |
| Phenolic compounds | p-Coumaric acid | 4.079 | 163.0415 | [M-H]^-^ | 1 | [±] |  |  |  | x |  |  | x |  |  |  |  |  |  |
| Amino compounds | Phenylalanine | 1.741 | 166.0861 | [M+H]^+^ | 1 | [±] |  | x |  | x |  |  | x | x |  |  |  |  |  |
| Other acid compounds | Phosphoric acid | 0.76 | 96.96006 | [M-H]^-^ | 1 | [±] |  | x |  | x |  | x |  |  |  |  |  |  |  |
| Amino compounds | Proline | 0.68 | 116.0701 | [M+H]^+^ | 1 | [±] |  | x |  | x |  | x | x |  |  |  |  |  |  |
| Other acid compounds | Pyruvic acid | 0.856 | 87.00867 | [M-H]^-^ | 1 | [±] |  | x |  | x |  | x | x |  |  |  |  |  |  |
| Flavonoids | Rutin | 5.071 | 611.1594 | [M+H]^+^ | 1 | [±] |  | x |  |  |  |  |  |  | x |  |  |  |  |
| Lipids | Stearic acid | 14.044 | 283.2664 | [M-H]^-^ | 1 | [±] |  |  |  | x |  |  |  |  |  |  |  | x |  |
| Other acid compounds | Succinic acid | 0.938 | 117.0201 | [M-H]^-^ | 1 | [±] |  | x |  |  |  |  | x |  |  |  |  |  |  |
| Amino compounds | Tryptophan | 3.346 | 205.0967 | [M+H]^+^ | 1 | [±] |  | x |  | x |  |  |  | x | x |  |  |  |  |
| Amino compounds | Tyrosine | 0.945 | 182.0808 | [M+H]^+^ | 1 | [±] |  | x |  |  |  |  | x |  |  |  |  |  |  |
| Amino compounds | Adenine | 0.802 | 136.0616 | [M+H]^+^ | 2 | [±] |  | x |  | x |  |  | x | x | x |  |  |  |  |
| Flavonoids | Epicatechin | 2.948 | 291.0856 | [M+H]^+^ | 2 | [±] |  |  |  | x |  |  | x | x |  |  |  |  |  |
| Amino compounds | Hydroxyproline | 0.656 | 132.0653 | [M+H]^+^ | 2 | [±] |  | x |  |  |  | x | x |  |  |  |  |  |  |
| Other nitrogen compounds | Hydroxyquinoline | 3.774 | 146.0598 | [M+H]^+^ | 2 | [±] |  |  |  | x |  |  |  |  | x |  |  |  |  |
| Flavonoids | Isorhamnetin-Hexosyl-Deoxyhexoside | 6.338 | 625.1745 | [M+H]^+^ | 2 | [±] |  | x |  |  |  |  |  |  | x |  |  |  |  |
| Flavonoids | Isorhamnetin-Hexosyl-Deoxyhexoside | 6.53 | 625.1752 | [M+H]^+^ | 2 | [±] |  | x |  |  |  |  |  |  | x |  |  |  |  |
| Flavonoids | Kaempferol-Hexosyl-Deoxyhexoside | 5.664 | 595.1638 | [M+H]^+^ | 2 | [±] |  | x |  |  |  |  |  |  | x |  |  |  |  |
| Flavonoids | Kaempferol-Hexosyl-Deoxyhexoside | 6.016 | 595.1644 | [M+H]^+^ | 2 | [±] |  | x |  |  |  |  |  |  | x |  |  |  |  |
| Flavonoids | Quercetin-Dihexoside | 4.112 | 627.1544 | [M+H]^+^ | 2 | [±] |  | x |  |  |  |  |  |  | x |  |  |  |  |
| Flavonoids | Rhamnetin-Dihexoside | 4.618 | 641.17 | [M+H]^+^ | 2 | [±] |  | x |  |  |  |  |  |  | x |  |  |  |  |
| Carbohydrates | Sucrose | 0.646 | 365.1052 | [M+Na]^+^ | 2 | [±] |  |  |  | x |  |  | x |  |  |  |  |  |  |
| Phenolic compounds | Aminophenol | 1.011 | 110.0599 | [M+H]^+^ | 3 | [+] |  | x |  | x |  |  |  |  | x |  |  |  |  |
| Other acid compounds | Azelaic acid | 4.816 | 187.0996 | [M-H]^-^ | 3 | [-] |  |  |  | x |  |  |  | x |  |  |  |  |  |
| Other acid compounds | Benzoic acid | 3.677 | 121.0294 | [M-H]^-^ | 3 | [-] |  | x |  |  |  |  |  |  | x |  |  |  |  |
| Amino compounds | Betaine | 0.686 | 118.0859 | [M+H]^+^ | 3 | [+] |  | x |  | x |  | x | x |  |  |  |  |  |  |
| Phenolic compounds | Caffeic acid | 2.094 | 179.035 | [M-H]^-^ | 3 | [-] |  |  |  | x |  |  | x |  |  |  |  |  |  |
| Phenolic compounds | Catechol | 2.063 | 109.0295 | [M-H]^-^ | 3 | [-] |  | x |  |  |  |  | x |  |  |  |  |  |  |
| Amino compounds | Choline | 0.777 | 104.1068 | [M]^+^ | 3 | [+] |  | x |  | x |  | x | x |  |  |  |  |  |  |
| Other acid compounds | Citramalic acid | 0.983 | 147.03 | [M-H]^-^ | 3 | [-] |  | x |  |  |  | x | x |  |  |  |  |  |  |
| Phenolic compounds | Coniferyl aldehyde | 3.357 | 177.0572 | [M-H]^-^ | 3 | [-] |  |  |  | x |  |  | x |  | x |  |  |  |  |
| Phenolic compounds | Coniferyl aldehyde | 4.934 | 177.0572 | [M-H]^-^ | 3 | [-] |  |  |  | x |  |  |  |  | x |  |  |  |  |
| Phenolic compounds | Coumaric acid | 3.297 | 163.0401 | [M-H]^-^ | 3 | [-] |  | x |  |  |  |  | x | x |  |  |  |  |  |
| Phenolic compounds | Coumaric acid | 0.968 | 147.0437 | [M-H_2_O+H]^+^ | 3 | [+] |  | x |  |  |  |  | x |  |  |  |  |  |  |
| Phenolic compounds | Coumaric acid | 2.135 | 147.0437 | [M-H_2_O+H]^+^ | 3 | [+] |  | x |  |  |  |  | x |  |  |  |  |  |  |
| Coumarins | Coumaric acid-Hexoside | 3.319 | 325.0927 | [M-H]^-^ | 3 | [-] |  | x |  |  |  |  | x | x |  |  |  |  |  |
| Phenolic compounds | Coumaroylquinic acid | 3.355 | 337.0961 | [M-H]^-^ | 3 | [-] |  | x |  |  |  |  | x |  |  |  |  |  |  |
| Flavonoids | Cyanidin-Hexosyl-Pentoside | 3.653 | 581.1487 | [M]^+^ | 3 | [+] |  | x |  |  |  |  |  | x | x |  |  |  |  |
| Phenolic compounds | Dihydroxybenzoic acid | 1.887 | 153.0209 | [M-H]^-^ | 3 | [-] |  | x |  |  |  |  | x |  |  |  |  |  |  |
| Phenolic compounds | Dihydroxybenzoic acid | 2.575 | 153.0199 | [M-H]^-^ | 3 | [-] |  | x |  | x |  |  | x |  |  |  |  |  |  |
| Phenolic compounds | Dihydroxybenzoic acid | 4.212 | 153.0209 | [M-H]^-^ | 3 | [-] |  |  |  | x |  |  | x |  |  |  |  |  |  |
| Phenolic compounds | Dihydroxybenzoic acid | 4.534 | 153.0209 | [M-H]^-^ | 3 | [-] |  | x |  |  |  |  |  | x |  |  |  |  |  |
| Flavonoids | Epicatechin | 3.606 | 289.0716 | [M-H]^-^ | 3 | [-] |  |  |  | x |  |  |  |  | x |  |  |  |  |
| Phenolic compounds | Flavaspidic acid | 3.6 | 417.1551 | [M-H]^-^ | 3 | [-] |  | x |  |  |  |  |  |  | x |  |  |  |  |
| Phenolic compounds | Flavaspidic acid | 5.81 | 417.1551 | [M-H]^-^ | 3 | [-] |  | x |  |  |  |  |  |  | x |  |  |  |  |
| Other acid compounds | Fumaric acid | 0.671 | 115.0036 | [M-H]^-^ | 3 | [-] |  | x |  | x |  | x | x |  |  |  |  |  |  |
| Carbohydrates | Galactitol | 0.613 | 181.0737 | [M-H]^-^ | 3 | [-] |  | x |  |  |  | x | x |  |  |  |  |  |  |
| Other acid compounds | Gluconic acid | 0.634 | 195.0532 | [M-H]^-^ | 3 | [-] |  | x |  | x |  | x | x |  |  |  |  |  |  |
| Amino compounds | Guanine | 1.158 | 152.0563 | [M+H]^+^ | 3 | [+] |  |  |  | x |  |  | x | x |  |  |  |  |  |
| Lipids | Heptadecanoate | 13.726 | 269.2505 | [M-H]^-^ | 3 | [-] |  |  |  | x |  |  |  |  |  |  |  | x |  |
| Phenolic compounds | Hydroxybenzaldehyde | 1.01 | 123.0438 | [M+H]^+^ | 3 | [+] |  | x |  |  |  |  |  |  |  |  |  |  |  |
| Phenolic compounds | Hydroxybenzaldehyde | 2.336 | 121.0296 | [M-H]^-^ | 3 | [-] |  |  |  | x |  |  | x |  |  |  |  |  |  |
| Phenolic compounds | Hydroxybenzaldehyde | 3.597 | 121.0304 | [M-H]^-^ | 3 | [-] |  |  |  | x |  |  |  |  | x |  |  |  |  |
| Phenolic compounds | Hydroxybenzoic acid | 4.663 | 137.0259 | [M-H]^-^ | 3 | [-] |  | x |  | x |  |  | x | x |  |  |  |  |  |
| Lipids | Hydroxyoctadecadienoic acid | 12.383 | 295.2308 | [M-H]^-^ | 3 | [-] |  |  |  | x |  |  |  |  |  | x |  |  |  |
| Other acid compounds | Hydroxyphenyllactic acid | 2.868 | 181.0516 | [M-H]^-^ | 3 | [-] |  | x |  |  |  |  | x |  |  |  |  |  |  |
| Other acid compounds | Hydroxyphenyllactic acid | 4.916 | 181.0516 | [M-H]^-^ | 3 | [-] |  | x |  |  |  |  |  |  |  | x |  |  |  |
| Alkaloids | Hyoscyamine | 5.216 | 290.1742 | [M+H]^+^ | 3 | [+] |  |  |  | x |  | ? |  |  |  |  |  |  |  |
| Amino compounds | Indole-carboxyaldehyde | 3.344 | 146.0599 | [M+H]^+^ | 3 | [+] |  |  |  | x |  |  |  | x | x |  |  |  |  |
| Amino compounds | Indole-carboxyaldehyde | 4.985 | 144.047 | [M-H]^-^ | 3 | [-] |  |  |  | x |  |  |  |  |  | x |  |  |  |
| Phenolic compounds | Isoferulic acid | 2.075 | 193.0519 | [M-H]^-^ | 3 | [-] |  |  |  | x |  |  | x |  |  |  |  |  |  |
| Other acid compounds | Isopropylmalic acid | 2.801 | 175.0625 | [M-H]^-^ | 3 | [-] |  | x |  | x |  |  | x |  |  |  |  |  |  |
| Flavonoids | Isorhamnetin-Hexoside | 4.612 | 479.1174 | [M+H]^+^ | 3 | [+] |  | x |  |  |  |  |  |  | x |  |  |  |  |
| Flavonoids | Kaempferol-Dihexoside | 4.473 | 609.152 | [M-H]^-^ | 3 | [-] |  | x |  |  |  |  |  |  | x |  |  |  |  |
| Other acid compounds | Lactic acid | 0.785 | 89.02429 | [M-H]^-^ | 3 | [-] |  | x |  |  |  |  | x |  |  |  |  |  |  |
| Lipids | Linoleic acid | 13.317 | 279.2355 | [M-H]^-^ | 3 | [-] |  |  |  | x |  |  |  |  |  |  | x |  |  |
| Other acid compounds | Malic acid | 0.733 | 133.0143 | [M-H]^-^ | 3 | [-] |  | x |  | x |  | x | x |  |  |  |  |  |  |
| Carbohydrates | Maltose | 0.767 | 341.1124 | [M-H]^-^ | 3 | [-] |  |  |  | x |  | x |  |  |  |  |  |  |  |
| Phenolic compounds | Methoxysalicylic acid | 2.938 | 167.0364 | [M-H]^-^ | 3 | [-] |  | x |  |  |  |  | x |  |  |  |  |  |  |
| Phenolic compounds | Methoxysalicylic acid | 3.98 | 167.0364 | [M-H]^-^ | 3 | [-] |  | x |  |  |  |  |  | x | x |  |  |  |  |
| Coumarins | Methyldaphnetin | 6.842 | 191.0368 | [M-H]^-^ | 3 | [-] |  |  |  | x |  | x |  |  |  |  |  |  |  |
| Phenolic compounds | Neochlorogenic acid | 3.083 | 353.0904 | [M-H]^-^ | 3 | [-] |  | x |  | x |  |  | x |  |  |  |  |  |  |
| Amino compounds | Niacinamide | 0.896 | 123.0551 | [M+H]^+^ | 3 | [+] |  | x |  | x |  |  | x | x |  |  |  |  |  |
| Other acid compounds | Octadecanedioic acid | 12.141 | 313.2395 | [M-H]^-^ | 3 | [-] |  |  |  | x |  |  |  |  |  | x |  |  |  |
| Other acid compounds | Octadecanedioic acid | 12.704 | 313.2395 | [M-H]^-^ | 3 | [-] |  |  |  | x |  |  |  |  |  | x |  |  |  |
| Other nitrogen compounds | Octopamine | 0.965 | 136.0754 | [M-H_2_O+H]^+^ | 3 | [+] |  | x |  |  |  |  | x |  |  |  |  |  |  |
| Flavonoids | Peltatoside | 4.33 | 595.1362 | [M-H]^-^ | 3 | [-] |  | x |  |  |  |  |  |  | x |  |  |  |  |
| Other acid compounds | Phenylacetic Acid | 2.11 | 137.0596 | [M+H]^+^ | 3 | [+] |  |  |  | x |  |  | x |  |  |  |  |  |  |
| Other nitrogen compounds | Phenylethanolamine | 1.73 | 120.0807 | [M-H_2_O+H]^+^ | 3 | [+] |  | x |  | x |  |  | x | x |  |  |  |  |  |
| Amino compounds | Pipecolic acid | 0.793 | 130.086 | [M+H]^+^ | 3 | [+] |  |  |  | x |  | x | x |  |  |  |  |  |  |
| Other acid compounds | Piscidic Acid | 0.873 | 255.051 | [M-H]^-^ | 3 | [-] |  |  |  | x |  | x |  |  |  |  |  |  |  |
| Other acid compounds | Piscidic Acid | 2.06 | 255.0542 | [M-H]^-^ | 3 | [-] |  | x |  | x |  |  | x |  |  |  |  |  |  |
| Phenolic compounds | Protocatechuic aldehyde | 3.17 | 137.026 | [M-H]^-^ | 3 | [-] |  |  |  | x |  |  |  |  | x |  |  |  |  |
| Amino compounds | Pyroglutamic acid | 0.733 | 128.0353 | [M-H]^-^ | 3 | [-] |  | x |  | x |  | x | x |  |  |  |  |  |  |
| Other acid compounds | Pyruvate | 0.686 | 87.00936 | [M]- | 3 | [-] |  |  |  | x |  | x |  |  |  |  |  |  |  |
| Phenolic compounds | Rosmarinic acid | 3.088 | 163.0384 | [M+H-C_9_H_10_O_5_]^+^ | 3 | [+] |  | x |  |  |  |  | x |  |  |  |  |  |  |
| Other acid compounds | Sebacic acid | 6.288 | 201.1147 | [M-H]^-^ | 3 | [-] |  |  |  | x |  |  |  | x |  |  |  |  |  |
| Phenolic compounds | Sinapoyl aldehyde | 5.23 | 207.0675 | [M-H]^-^ | 3 | [-] |  |  |  | x |  |  |  |  | x |  |  |  |  |
| Other acid compounds | Suberic acid | 3.873 | 173.0831 | [M-H]^-^ | 3 | [-] |  |  |  | x |  |  | x |  |  |  |  |  |  |
| Other acid compounds | Succinic acid derivate | 0.701 | 117.0201 | [M-H]^-^ | 3 | [-] |  | x |  |  |  | x | x |  |  |  |  |  |  |
| Lipids | Trihydroxyoctadecenoic acid | 11.715 | 329.2369 | [M-H]^-^ | 3 | [-] |  |  |  | x |  |  |  |  |  | x |  |  |  |
| Amino compounds | Tyrosine derivate | 3.672 | 182.0808 | [M+H]^+^ | 3 | [+] |  |  |  | x |  |  |  |  | x |  |  |  |  |
| Amino compounds | Uracil | 0.815 | 113.0341 | [M+H]^+^ | 3 | [+] |  |  |  | x |  |  | x |  |  |  |  |  |  |
| Coumarins | Xanthotoxin | 8.054 | 217.0489 | [M+H]^+^ | 3 | [+] |  | x |  |  |  |  |  |  |  | x |  |  |  |

**Tab. S8.** Overview of all compounds found in *S. nigra*.

|  |  |  |  |  |  |  | Plant Part | | | | | Fraction | | | | | | | |
| --- | --- | --- | --- | --- | --- | --- | --- | --- | --- | --- | --- | --- | --- | --- | --- | --- | --- | --- | --- |
| Ontology | Compound Name | RT | Precursor mass | Adduct | Level | Polarity | Flowers | Berries | Leaves | Roots | Bark | 1 | 2 | 3 | 4 | 5 | 6 | 7 | 8 |
| Phenolic compounds | 4-Hydroxybenzoic acid | 3.154 | 137.02467 | [M-H]^-^ | 1 | [±] |  |  |  |  | x |  |  |  |  | x |  |  |  |
| Other nitrogen compounds | Adenosine | 1.166 | 268.10352 | [M+H]^+^ | 1 | [±] | x | x | x |  | x |  | x | x | x |  |  |  |  |
| Amino compounds | Asparagine | 0.65 | 133.06059 | [M+H]^+^ | 1 | [±] | x |  | x |  | x | x | x |  |  |  |  |  |  |
| Amino compounds | Aspartic acid | 0.617 | 132.03149 | [M-H]^-^ | 1 | [±] |  |  |  |  | x | x | x |  |  |  |  |  |  |
| Phenolic compounds | Caffeic acid | 3.419 | 179.03525 | [M-H]^-^ | 1 | [±] |  |  |  |  | x |  |  |  | x |  |  |  |  |
| Flavonoids | Catechin | 3.35 | 289.07367 | [M-H]^-^ | 1 | [±] |  |  |  |  | x |  | x | x | x |  |  |  |  |
| Phenolic compounds | Chlorogenic acid | 3.554 | 377.08414 | [M+Na]^+^ | 1 | [±] | x |  | x |  | x |  | x | x |  |  |  |  |  |
| Other acid compounds | Citric acid | 0.855 | 191.02008 | [M-H]^-^ | 1 | [±] |  |  | x |  | x | x | x |  |  |  |  |  |  |
| Phenolic compounds | Ferulic acid | 4.464 | 193.05099 | [M-H]^-^ | 1 | [±] |  |  |  |  | x |  |  | x |  |  |  |  |  |
| Other acid compounds | Fumaric Acid | 0.718 | 115.00409 | [M-H]^-^ | 1 | [±] | x | x |  |  |  | x | x |  |  |  |  |  |  |
| Phenolic compounds | Gallic acid | 1.453 | 169.01471 | [M-H]^-^ | 1 | [±] | x | x | x |  |  |  | x |  |  |  |  |  |  |
| Flavonoids | Isoquercetin | 5.246 | 463.09344 | [M-H]^-^ | 1 | [±] | x | x | x |  |  |  |  |  | x | x |  |  |  |
| Flavonoids | Kaempferol | 11.694 | 285.04315 | [M-H]^-^ | 1 | [±] | x |  |  |  |  |  |  |  |  | x |  |  |  |
| Other nitrogen compounds | Kynurenic acid | 3.831 | 190.04962 | [M+H]^+^ | 1 | [±] |  |  | x |  |  |  | x |  |  |  |  |  |  |
| Amino compounds | Leucine | 1.057 | 130.08839 | [M-H]^-^ | 1 | [±] | x | x | x |  | x |  | x | x |  |  |  |  |  |
| Flavonoids | Naringenin | 9.84 | 273.07593 | [M+H]^+^ | 1 | [±] | x |  | x |  |  |  |  |  |  | x |  |  |  |
| Other acid compounds | Nicotinic acid | 0.896 | 124.03914 | [M+H]^+^ | 1 | [±] |  | x |  |  | x |  | x |  |  |  |  |  |  |
| Lipids | Palmitic acid | 13.386 | 255.23581 | [M-H]^-^ | 1 | [±] | x |  | x | x | x |  |  |  |  |  | x | x |  |
| Coumarins | p-Coumaric acid | 4.071 | 163.04182 | [M-H]^-^ | 1 | [±] | x | x | x |  | x |  |  | x | x |  |  |  |  |
| Amino compounds | Phenylalanine | 1.758 | 164.07176 | [M-H]^-^ | 1 | [±] | x | x | x |  | x |  | x | x | x |  |  |  |  |
| Amino compounds | Proline | 0.684 | 116.07032 | [M+H]^+^ | 1 | [±] | x |  | x |  | x | x | x |  |  |  |  |  |  |
| Other acid compounds | Pyruvic acid | 0.847 | 87.00871 | [M-H]^-^ | 1 | [±] | x | x |  |  | x | x | x |  |  |  |  |  |  |
| Flavonoids | Rutin | 5.079 | 609.15308 | [M-H]^-^ | 1 | [±] | x | x | x |  |  |  |  |  | x |  |  |  |  |
| Lipids | Stearic acid | 13.93 | 283.26736 | [M-H]^-^ | 1 | [±] | x | x | x |  |  |  |  |  |  |  |  | x | x |
| Other acid compounds | Succinic acid | 0.932 | 117.01945 | [M-H]^-^ | 1 | [±] | x |  | x |  | x | x | x |  |  |  |  |  |  |
| Amino compounds | Tryptophan | 3.357 | 203.08322 | [M-H]^-^ | 1 | [±] | x | x | x |  | x |  |  | x | x |  |  |  |  |
| Amino compounds | Tyrosine | 1.026 | 180.06677 | [M-H]^-^ | 1 | [±] | x | x | x |  | x |  | x | x |  |  |  |  |  |
| Lipids | Vaccenic acid | 13.703 | 281.24878 | [M-H]^-^ | 1 | [±] | x |  |  |  |  |  |  |  |  |  |  | x |  |
| Amino compounds | Valine | 0.718 | 118.08598 | [M+H]^+^ | 1 | [±] | x | x |  |  | x | x | x |  |  |  |  |  |  |
| Phenolic compounds | Vanillin | 3.967 | 153.05467 | [M+H]^+^ | 1 | [±] |  |  |  |  | x |  |  |  | x |  |  |  |  |
| Lipids | y-Linolenic acid | 13.061 | 277.21982 | [M-H]^-^ | 1 | [±] | x |  | x |  | x |  |  |  |  |  | x |  |  |
| Amino compounds | Adenine | 0.807 | 134.04846 | [M-H]^-^ | 2 | [±] | x | x | x |  | x |  |  | x | x |  |  |  |  |
| Amino compounds | Adenine | 1.186 | 134.04732 | [M-H]^-^ | 2 | [±] | x |  | x |  |  |  | x | x | x |  |  |  |  |
| Flavonoids | Afzelechin | 3.891 | 273.07883 | [M-H]^-^ | 2 | [±] |  |  |  |  | x |  |  |  | x |  |  |  |  |
| Terpenes & Terpenoids | alpha-Boswellic acid / Betulinic Acid | 13.213 | 455.35687 | [M-H]^-^ | 2 | [±] |  |  |  | x | x |  |  |  |  |  |  | x |  |
| Amino compounds | Arginine | 0.617 | 173.10454 | [M-H]^-^ | 2 | [±] |  | x |  |  |  | x | x | x |  |  |  |  |  |
| Other acid compounds | Benzoic acid | 3.61 | 121.03069 | [M-H]^-^ | 2 | [±] | x | x | x | x | x |  | x | x | x |  |  |  |  |
| Other acid compounds | Caffeoylquinic acid | 3.442 | 353.09158 | [M-H]^-^ | 2 | [±] | x |  | x |  | x |  | x | x | x |  |  |  |  |
| Flavonoids | Catechin derivate | 3.623 | 289.0744 | [M-H]^-^ | 2 | [±] | x |  |  |  | x |  |  |  | x |  |  |  |  |
| Other acid compounds | Coumaroyl quinic acid | 3.847 | 337.09576 | [M-H]^-^ | 2 | [±] | x |  | x |  |  |  | x | x |  |  |  |  |  |
| Phenolic compounds | Cynarine | 4.885 | 517.13269 | [M+H]^+^ | 2 | [±] | x |  | x |  |  |  |  |  | x |  |  |  |  |
| Carbohydrates | Dihexoside | 0.616 | 341.10989 | [M-H]^-^ | 2 | [±] |  |  |  |  | x | x | x |  |  |  |  |  |  |
| Others | Dihydrokaempferol | 5.274 | 287.05853 | [M-H]^-^ | 2 | [±] | x |  |  |  |  |  |  |  | x |  |  |  |  |
| Phenolic compounds | Dopamine | 0.951 | 152.07286 | [M-H]^-^ | 2 | [±] | x |  | x |  |  |  | x |  |  |  |  |  |  |
| Flavonoids | Flavovilloside/ Alcesefoliside | 4.206 | 755.21143 | [M-H]^-^ | 2 | [±] | x | x |  |  |  |  |  |  | x |  |  |  |  |
| Lipids | Glochidioboside | 4.784 | 521.20679 | [M-H]^-^ | 2 | [±] |  |  |  |  | x |  |  |  | x |  |  |  |  |
| Other nitrogen compounds | Guanine | 1.194 | 150.04276 | [M-H]^-^ | 2 | [±] | x |  |  |  |  |  | x |  |  |  |  |  |  |
| Other nitrogen compounds | Guanosine | 1.178 | 282.08453 | [M-H]^-^ | 2 | [±] | x |  |  |  |  |  | x | x |  |  |  |  |  |
| Amino compounds | Histidine | 0.594 | 154.06223 | [M-H]^-^ | 2 | [±] | x |  | x |  | x | x | x | x |  |  |  |  |  |
| Carbohydrates | Hexoside alcohol | 0.609 | 181.07266 | [M-H]^-^ | 2 | [±] |  |  |  |  | x | x | x |  |  |  |  |  |  |
| Phenolic compounds | Hydroxybenzoic acid | 1.864 | 137.02466 | [M-H]^-^ | 2 | [±] | x | x | x |  | x |  | x | x | x |  |  |  |  |
| Flavonoids | Isorhamnetin-O-hexosyl-deoxyhexoside | 6.5 | 623.16895 | [M-H]^-^ | 2 | [±] | x | x | x |  |  |  |  |  | x | x |  |  |  |
| Flavonoids | Kaempferol-O-hexosyl-deoxyhexoside | 6.018 | 593.15759 | [M-H]^-^ | 2 | [±] | x | x | x |  |  |  |  |  | x | x |  |  |  |
| Lipids | MGDG O-16:3 | 12.539 | 537.30383 | [M+Na]^+^ | 2 | [±] |  |  | x |  |  |  |  |  |  |  | x |  |  |
| Flavonoids | Naringenin-Hexoside | 5.334 | 433.11823 | [M-H]^-^ | 2 | [±] | x |  |  |  |  |  |  |  | x |  |  |  |  |
| Phenolic compounds | Neochlorogenic acid | 3.075 | 355.10205 | [M+H]^+^ | 2 | [±] | x |  | x |  | x |  | x |  |  |  |  |  |  |
| Amino compounds | N-Fructosyl isoleucine | 1.156 | 292.14087 | [M-H]^-^ | 2 | [±] |  | x |  |  |  |  | x | x |  |  |  |  |  |
| Amino compounds | Oxoproline | 0.896 | 128.03552 | [M-H]^-^ | 2 | [±] | x |  | x |  | x | x | x |  |  |  |  |  |  |
| Other acid compounds | Pipecolic acid | 0.786 | 130.08604 | [M+H]^+^ | 2 | [±] | x | x | x |  |  | x | x |  |  |  |  |  |  |
| Flavonoids | Quercetin-Acetylhexoside | 6.364 | 505.10391 | [M-H]^-^ | 2 | [±] | x |  | x |  |  |  |  |  | x | x |  |  |  |
| Flavonoids | Quercetin-Malonylhexoside | 5.679 | 549.09381 | [M-H]^-^ | 2 | [±] | x |  |  |  |  |  |  |  | x |  |  |  |  |
| Flavonoids | Quercetin-O-dihexoside | 4.089 | 625.14722 | [M-H]^-^ | 2 | [±] | x |  | x |  |  |  |  |  | x |  |  |  |  |
| Phenolic compounds | QuinicAcid | 0.667 | 191.05725 | [M-H]^-^ | 2 | [±] | x | x |  |  |  | x | x |  |  |  |  |  |  |
| Flavonoids | Rhamnetin-O-dihexoside | 4.608 | 639.16187 | [M-H]^-^ | 2 | [±] | x |  |  |  |  |  |  |  |  |  |  |  |  |
| Alkaloids | Salsolinol | 1.162 | 178.08885 | [M-H]^-^ | 2 | [±] | x |  | x |  |  |  | x |  |  |  |  |  |  |
| Phenolic compounds | Syringic acid | 3.255 | 197.04639 | [M-H]^-^ | 2 | [±] |  |  |  | x | x |  | x |  |  |  |  |  |  |
| Amino compounds | Tyramine | 1.174 | 138.09109 | [M+H]^+^ | 2 | [±] |  |  | x |  |  |  | x |  |  |  |  |  |  |
| Phenolic compounds | Vanillic acid | 2.951 | 167.03542 | [M-H]^-^ | 2 | [±] |  |  |  |  | x |  | x |  |  |  |  |  |  |
| Phenolic compounds | Acanthoside B | 5.83 | 579.21301 | [M-H]^-^ | 3 | [-] |  |  | x |  |  |  |  |  | x |  |  |  |  |
| Other acid compounds | Acteoside-Derivative | 4.473 | 623.19818 | [M-H]^-^ | 3 | [-] |  |  |  |  | x |  |  |  | x |  |  |  |  |
| Coumarins | Acteoside-Derivative | 5.13 | 623.19812 | [M-H]^-^ | 3 | [-] |  |  |  |  | x |  |  |  | x |  |  |  |  |
| Amino compounds | Adenine | 3.241 | 136.06143 | [M+H]^+^ | 3 | [+] | x |  |  |  |  |  |  |  | x |  |  |  |  |
| Other nitrogen compounds | Adenine | 3.298 | 134.04732 | [M-H]^-^ | 3 | [-] | x |  |  |  |  |  | x |  | x |  |  |  |  |
| Other nitrogen compounds | Adenosine monophosphate | 0.79 | 346.05878 | [M-H]^-^ | 3 | [-] | x |  |  |  |  |  | x |  |  |  |  |  |  |
| Amino compounds | Agmatine | 0.598 | 131.12898 | [M+H]^+^ | 3 | [+] | x |  | x |  |  | x |  |  |  |  |  |  |  |
| Amino compounds | allo-Threonine | 0.627 | 118.05108 | [M-H]^-^ | 3 | [-] |  | x |  |  |  | x | x |  |  |  |  |  |  |
| Amino compounds | amino-hydroxypropanoic acid | 0.602 | 104.03625 | [M-H]^-^ | 3 | [-] | x |  |  |  |  | x | x |  |  |  |  |  |  |
| Other acid compounds | Amygdalin | 3.238 | 480.14676 | [M+Na]^+^ | 3 | [+] |  |  | x |  |  |  |  |  | x |  |  |  |  |
| Lipids | Arachidic acid | 14.605 | 311.29782 | [M-H]^-^ | 3 | [-] | x |  |  |  | x |  |  |  |  |  |  | x | x |
| Coumarins | Archangelicine | 12.61 | 449.15726 | [M+Na]^+^ | 3 | [+] |  |  | x |  |  |  |  |  |  |  | x |  |  |
| Other acid compounds | Azelaic acid | 4.775 | 187.09926 | [M-H]^-^ | 3 | [-] | x | x | x |  | x |  |  |  | x | x |  |  |  |
| Lipids | Behenic acid | 15.152 | 339.32944 | [M-H]^-^ | 3 | [-] |  |  |  |  | x |  |  |  |  |  |  | x | x |
| Other acid compounds | Benzoic acid | 3.129 | 121.03071 | [M-H]^-^ | 3 | [-] |  |  | x |  |  |  |  |  | x |  |  |  |  |
| Others | Benzoic acid | 4.165 | 121.02975 | [M-H]^-^ | 3 | [-] |  |  |  | x |  |  |  |  | x |  |  |  |  |
| Phenolic compounds | Benzyl-hexosyl-pentoside | 3.453 | 425.14142 | [M+Na]^+^ | 3 | [+] | x |  | x |  |  |  |  |  | x |  |  |  |  |
| Phenolic compounds | Caffeic acid derivate | 3.638 | 179.03627 | [M-H]^-^ | 3 | [-] | x | x | x |  | x |  | x | x | x |  |  |  |  |
| Phenolic compounds | Caffeic acid derivate | 4.2 | 179.03629 | [M-H]^-^ | 3 | [-] |  |  | x |  |  |  |  |  | x |  |  |  |  |
| Phenolic compounds | Caffeoylquinic acid | 2.027 | 353.09189 | [M-H]^-^ | 3 | [-] | x |  | x |  |  | x |  |  |  |  |  |  |  |
| Phenolic compounds | Caffeoylquinic acid | 5.032 | 353.08783 | [M-H]^-^ | 3 | [-] |  |  | x |  |  |  |  |  | x |  |  |  |  |
| Other acid compounds | Caffeoylquinic acid | 5.34 | 353.09015 | [M-H]^-^ | 3 | [-] |  |  | x |  |  |  |  |  |  |  |  |  |  |
| Amino compounds | Carnitine | 0.709 | 162.11258 | [M+H]^+^ | 3 | [+] |  |  |  |  | x | x | x |  |  |  |  |  |  |
| Flavonoids | Catechol | 0.735 | 111.04378 | [M+H]^+^ | 3 | [+] | x |  |  |  |  | x |  |  |  |  |  |  |  |
| Phenolic compounds | Catechol | 2.588 | 109.03062 | [M-H]^-^ | 3 | [-] | x | x | x |  | x |  | x | x |  |  |  |  |  |
| Amino compounds | Choline | 0.698 | 104.10723 | [M]^+^ | 3 | [+] | x | x | x |  | x | x | x |  |  |  |  |  |  |
| Amino compounds | Choline | 13.571 | 104.10662 | [M]^+^ | 3 | [+] | x |  | x |  |  |  |  |  |  |  |  | x | x |
| Phenolic compounds | Cinnamic acid | 1.828 | 147.04524 | [M-H]^-^ | 3 | [-] |  | x | x |  |  |  | x | x | x |  |  |  |  |
| Other acid compounds | Citramalic acid | 0.837 | 147.03012 | [M-H]^-^ | 3 | [-] | x | x |  |  |  | x | x |  |  |  |  |  |  |
| Other acid compounds | Citramalic acid | 4.243 | 149.04486 | [M+H]^+^ | 3 | [+] |  | x |  |  |  |  |  | x |  |  |  |  |  |
| Others | Coniferyl aldehyde | 4.924 | 177.05592 | [M-H]^-^ | 3 | [-] |  |  |  | x |  |  |  |  | x |  |  |  |  |
| Phenolic compounds | Coumaric acid | 4.267 | 163.04059 | [M-H]^-^ | 3 | [-] |  |  |  |  | x |  |  | x |  |  |  |  |  |
| Coumarins | Coumaric acid | 2.975 | 163.04131 | [M-H]^-^ | 3 | [-] |  |  | x |  |  |  | x |  |  |  |  |  |  |
| Phenolic compounds | Coumarin | 1.785 | 147.04533 | [M+H]^+^ | 3 | [+] | x |  |  |  |  |  | x | x |  |  |  |  |  |
| Phenolic compounds | Coumarin | 4.595 | 147.04529 | [M-H]^-^ | 3 | [-] |  |  |  |  | x |  |  |  | x |  |  |  |  |
| Other acid compounds | Coumaroyl quinic acid | 3.364 | 337.09341 | [M-H]^-^ | 3 | [-] | x |  |  |  |  |  | x |  |  |  |  |  |  |
| Terpenes & Terpenoids | Crocetin glucosylester | 3.659 | 409.18311 | [M+Na]^+^ | 3 | [+] |  |  | x |  |  |  |  |  | x |  |  |  |  |
| Flavonoids | Cyanidin-O-hexosyl-pentoside | 3.694 | 581.14948 | [M]^+^ | 3 | [+] |  | x |  |  |  |  |  | x | x |  |  |  |  |
| Flavonoids | Cyanidin-O-trihexoside | 3.248 | 743.20166 | [M]^+^ | 3 | [+] |  | x |  |  |  |  |  | x | x |  |  |  |  |
| Phenolic compounds | Cynarine | 6.249 | 517.13342 | [M+H]^+^ | 3 | [+] | x |  | x |  |  |  |  |  | x |  |  |  |  |
| Other nitrogen compounds | Cytosine | 0.726 | 112.05025 | [M+H]^+^ | 3 | [+] | x |  |  |  |  |  | x |  |  |  |  |  |  |
| Coumarins | Daphnetin | 2.993 | 177.01984 | [M-H]^-^ | 3 | [-] |  |  | x |  |  |  | x |  |  |  |  |  |  |
| Coumarins | Daphnetin | 3.799 | 177.01987 | [M-H]^-^ | 3 | [-] |  |  |  | x |  | x |  |  |  |  |  |  |  |
| Others | Daphnetin-Hexoside | 3.415 | 339.07446 | [M-H]^-^ | 3 | [-] |  |  | x |  |  |  |  |  | x |  |  |  |  |
| Other acid compounds | Decanedioic acid | 6.284 | 201.11345 | [M-H]^-^ | 3 | [-] |  | x |  |  | x |  |  |  | x |  |  |  |  |
| Flavonoids | Delphinidin-O-hexosyl-deoxyhexoside | 6.106 | 609.15131 | [M-H]^-^ | 3 | [-] |  |  | x |  |  |  |  |  | x |  |  |  |  |
| Other nitrogen compounds | Deoxyguanosine monophosphate | 0.899 | 348.06973 | [M+H]^+^ | 3 | [+] | x |  |  |  |  |  | x |  |  |  |  |  |  |
| Others | Diacetyl | 0.799 | 85.02982 | [M-H]^-^ | 3 | [-] |  | x |  |  |  | x | x |  |  |  |  |  |  |
| Other acid compounds | Dicaffeoylquinic acid | 4.819 | 515.1261 | [M-H]^-^ | 3 | [-] | x |  | x |  |  |  |  | x | x |  |  |  |  |
| Other acid compounds | Dicaffeoylquinic acid | 5.256 | 515.12378 | [M-H]^-^ | 3 | [-] | x |  | x |  |  |  |  |  | x |  |  |  |  |
| Other acid compounds | Dicaffeoylquinic acid | 6.628 | 515.11963 | [M-H]^-^ | 3 | [-] |  |  | x |  |  |  |  |  | x |  |  |  |  |
| Flavonoids | Dihydrokaempferol | 5.447 | 287.05646 | [M-H]^-^ | 3 | [-] | x |  |  |  |  |  |  |  | x |  |  |  |  |
| Phenolic compounds | Dihydroxybenzoic acid | 1.93 | 153.01987 | [M-H]^-^ | 3 | [-] |  | x | x |  |  |  | x |  |  |  |  |  |  |
| Phenolic compounds | Dihydroxybenzoic acid | 4.294 | 153.021 | [M-H]^-^ | 3 | [-] | x |  | x |  | x |  |  | x |  |  |  |  |  |
| Coumarins | Dihydroxycoumarin-Hexoside | 3.326 | 339.07227 | [M-H]^-^ | 3 | [-] |  |  | x |  |  |  |  | x | x |  |  |  |  |
| Lipids | Dodecanedioic acid | 10.401 | 229.1447 | [M-H]^-^ | 3 | [-] |  |  | x |  |  |  |  |  |  | x |  |  |  |
| Lipids | Dodecanedioic acid | 11.46 | 229.14632 | [M-H]^-^ | 3 | [-] | x | x | x |  | x |  |  |  | x | x |  |  |  |
| Lipids | Epoxystearic acid | 12.822 | 297.24561 | [M-H]^-^ | 3 | [-] | x |  |  |  |  |  |  |  |  | x | x |  |  |
| Coumarins | Esculetin | 3.275 | 177.02107 | [M-H]^-^ | 3 | [-] |  |  | x |  |  |  |  | x | x |  |  |  |  |
| Coumarins | Esculetin | 3.691 | 177.02101 | [M-H]^-^ | 3 | [-] | x |  | x |  |  |  |  | x | x |  |  |  |  |
| Coumarins | Esculetin | 9.993 | 177.02098 | [M-H]^-^ | 3 | [-] | x |  |  |  |  |  |  |  |  | x |  |  |  |
| Phenolic compounds | Ferulate | 3.526 | 177.0547 | [M-H_2_O+H]^+^ | 3 | [+] | x |  |  |  |  |  | x | x |  |  |  |  |  |
| Phenolic compounds | Ferulate | 8.119 | 177.05472 | [M-H_2_O+H]^+^ | 3 | [+] | x |  |  |  |  |  |  |  |  | x |  |  |  |
| Phenolic compounds | Ferulate | 9.76 | 177.05466 | [M-H_2_O+H]^+^ | 3 | [+] | x |  |  |  |  |  |  |  | x | x |  |  |  |
| Phenolic compounds | Feruloylquinic acid | 3.53 | 367.10678 | [M-H]^-^ | 3 | [-] |  |  |  |  |  |  | x | x | x |  |  |  |  |
| Other acid compounds | Feruloylquinic acid | 4.124 | 367.1037 | [M-H]^-^ | 3 | [-] | x |  | x |  |  |  | x | x | x |  |  |  |  |
| Other acid compounds | Furoic acid | 0.78 | 111.00941 | [M-H]^-^ | 3 | [-] | x | x |  | x | x | x | x |  |  |  |  |  |  |
| Other acid compounds | Furoic acid | 1.436 | 111.00866 | [M-H]^-^ | 3 | [-] |  | x |  |  |  |  | x |  |  |  |  |  |  |
| Other acid compounds | Furoic acid | 2.546 | 111.00913 | [M-H]^-^ | 3 | [-] |  | x |  |  |  |  | x |  |  |  |  |  |  |
| Phenolic compounds | Gaultherin | 3.621 | 469.13071 | [M+Na]^+^ | 3 | [+] | x |  |  |  |  |  |  |  | x |  |  |  |  |
| Phenolic compounds | Gentisic acid-Hexoside | 2.659 | 315.0726 | [M-H]^-^ | 3 | [-] |  |  |  |  | x |  | x |  |  |  |  |  |  |
| Carbohydrates | Gluconic acid | 0.631 | 195.05275 | [M-H]^-^ | 3 | [-] | x | x | x |  | x | x | x | x |  |  |  |  |  |
| Lipids | Glycero-phosphocholine | 0.686 | 258.10977 | [M+H]^+^ | 3 | [+] | x |  | x |  |  | x | x |  |  |  |  |  |  |
| Other acid compounds | Guanidinobutanoate | 0.786 | 146.09212 | [M+H]^+^ | 3 | [+] | x | x | x |  | x | x | x |  |  |  |  |  |  |
| Amino compounds | Guanine | 0.795 | 152.05623 | [M+H]^+^ | 3 | [+] | x |  |  |  |  |  | x | x |  |  |  |  |  |
| Flavonoids | Hesperitin | 11.267 | 301.07425 | [M-H]^-^ | 3 | [-] | x |  |  |  |  |  |  |  |  | x |  |  |  |
| Terpenes & Terpenoids | Hexenyl-hexosyl-pentoside | 3.789 | 417.17258 | [M+Na]^+^ | 3 | [+] | x |  |  |  |  |  |  |  | x |  |  |  |  |
| Phenolic compounds | Homovanillate | 3.497 | 137.05954 | [M+H]^+^ | 3 | [+] |  |  |  |  | x |  |  |  | x |  |  |  |  |
| Phenolic compounds | Hydroxybenzaldehyde | 1.002 | 123.04377 | [M+H]^+^ | 3 | [+] | x | x | x |  |  |  | x |  |  |  |  |  |  |
| Phenolic compounds | Hydroxybenzaldehyde | 2.552 | 123.04524 | [M-H]^-^ | 3 | [-] |  | x |  |  |  |  |  |  | x |  |  |  |  |
| Phenolic compounds | Hydroxybenzoic acid | 4.712 | 137.02599 | [M-H]^-^ | 3 | [-] | x | x | x |  |  |  |  | x |  |  |  |  |  |
| Phenolic compounds | Hydroxybenzyl alcohol | 2.46 | 123.04559 | [M-H]^-^ | 3 | [-] |  |  | x |  |  |  | x | x |  |  |  |  |  |
| Phenolic compounds | Hydroxyphenyllactic acid | 2.895 | 181.05098 | [M-H]^-^ | 3 | [-] |  | x |  |  |  |  | x | x |  |  |  |  |  |
| Phenolic compounds | Hydroxyphenyl-oxopropyl-Hexoside | 3.278 | 327.11075 | [M-H]^-^ | 3 | [-] |  | x |  |  |  |  |  | x | x |  |  |  |  |
| Amino compounds | Hydroxyproline | 0.638 | 130.05107 | [M-H]^-^ | 3 | [-] |  | x |  |  |  | x | x |  |  |  |  |  |  |
| Other nitrogen compounds | Hydroxyquinoline | 5.14 | 144.04536 | [M-H]^-^ | 3 | [-] |  | x | x |  | x |  |  |  |  | x |  |  |  |
| Other acid compounds | Hydroxysuberic acid | 3.636 | 189.07869 | [M-H]^-^ | 3 | [-] | x |  |  |  |  |  | x | x |  |  |  |  |  |
| Amino compounds | Indole | 1.037 | 118.06497 | [M+H]^+^ | 3 | [+] |  | x |  |  |  |  | x | x |  |  |  |  |  |
| Other nitrogen compounds | Indole | 3.437 | 116.05086 | [M-H]^-^ | 3 | [-] | x |  |  |  | x |  |  | x | x |  |  |  |  |
| Other nitrogen compounds | Indole-3-carboxyaldehyde | 3.298 | 146.05969 | [M+H]^+^ | 3 | [+] | x | x | x |  | x |  |  |  | x |  |  |  |  |
| Other nitrogen compounds | Inosine | 1.133 | 267.07346 | [M-H]^-^ | 3 | [-] |  |  | x |  |  |  | x | x |  |  |  |  |  |
| Phenolic compounds | Isochlorogenic acid A | 6.147 | 499.12344 | [M-H_2_O+H]^+^ | 3 | [+] | x |  | x |  |  |  |  |  | x |  |  |  |  |
| Other acid compounds | Isopropylmalic acid | 2.771 | 175.06248 | [M-H]^-^ | 3 | [-] | x | x | x |  |  |  | x |  |  |  |  |  |  |
| Flavonoids | isorhamnetin | 8.687 | 317.06561 | [M+H]^+^ | 3 | [+] | x |  |  |  |  |  |  |  | x | x |  |  |  |
| Flavonoids | Kaempferol-O-dihexoside | 3.743 | 611.15955 | [M+H]^+^ | 3 | [+] | x |  |  |  |  |  |  |  | x |  |  |  |  |
| Flavonoids | Kaempferol-O-dihexoside | 4.469 | 609.15125 | [M-H]^-^ | 3 | [-] | x |  |  |  |  |  |  |  | x |  |  |  |  |
| Flavonoids | Kaempferol-p-coumaroyl-hexosyl-deoxyhexoside | 16.456 | 763.17688 | [M+Na]^+^ | 3 | [+] |  |  | x |  |  |  |  |  |  |  |  | x |  |
| Other acid compounds | Lactic acid | 0.708 | 89.02465 | [M-H]^-^ | 3 | [-] |  | x |  |  |  | x | x |  |  |  |  |  |  |
| Other acid compounds | Lactic acid | 0.803 | 89.02438 | [M-H]^-^ | 3 | [-] |  | x |  |  | x | x |  | x | x |  |  |  |  |
| Amino compounds | Leucine derivate | 0.762 | 130.08855 | [M-H]^-^ | 3 | [-] | x |  |  |  |  | x |  |  |  |  |  |  |  |
| Amino compounds | Leucine derivate | 3.505 | 130.08788 | [M-H]^-^ | 3 | [-] |  | x |  |  | x |  | x | x |  |  |  |  |  |
| Lipids | Lignoceric acid | 15.638 | 367.3606 | [M-H]^-^ | 3 | [-] |  |  |  |  | x |  |  |  |  |  |  |  | x |
| Terpenes & Terpenoids | Linalyl-O-hexosyl-pentoside | 7.085 | 471.2193 | [M+Na]^+^ | 3 | [+] | x |  |  |  |  |  |  |  |  | x |  |  |  |
| Others | Loliolide | 4.497 | 197.11711 | [M+H]^+^ | 3 | [+] |  |  | x |  |  |  |  |  |  | x |  |  |  |
| Lipids | LPA | 14.328 | 435.25558 | [M-H]^-^ | 3 | [-] | x |  |  |  |  |  |  |  |  |  |  |  | x |
| Lipids | LPA 16:0 | 14.048 | 409.23636 | [M-H]^-^ | 3 | [-] | x |  | x |  |  |  |  |  |  |  |  | x | x |
| Lipids | LPG 18:1 | 13.369 | 509.28842 | [M-H]^-^ | 3 | [-] | x |  |  |  |  |  |  |  |  | x |  |  |  |
| Lipids | Lyso-PA 18:2 | 13.804 | 433.2402 | [M-H]^-^ | 3 | [-] | x |  |  |  |  |  |  |  |  |  |  |  | x |
| Lipids | Lyso-PA 18:3 | 13.46 | 431.22043 | [M-H]^-^ | 3 | [-] | x |  |  |  |  |  |  |  |  |  |  |  | x |
| Lipids | Lyso-PC 16:0 | 13.579 | 540.33594 | [M+HCOO]^-^ | 3 | [-] | x |  | x |  |  |  |  |  |  |  |  |  | x |
| Lipids | Lyso-PC 16:0 | 13.603 | 496.34039 | [M+H]^+^ | 3 | [+] | x |  |  |  |  |  |  |  |  |  |  |  | x |
| Lipids | Lyso-PC 18:2 | 13.417 | 564.33051 | [M-H]^-^ | 3 | [-] | x |  |  |  |  |  |  |  |  |  |  | x | x |
| Lipids | Lyso-PE 18:1 | 13.055 | 478.29791 | [M-H]^-^ | 3 | [-] | x |  |  |  |  |  |  |  |  |  |  | x |  |
| Lipids | Lyso-PE 18:2 | 12.817 | 476.27844 | [M-H]^-^ | 3 | [-] | x |  |  |  | x |  |  |  |  | x | x |  |  |
| Lipids | Lyso-PG 16:0 | 13.056 | 483.2774 | [M-H]^-^ | 3 | [-] | x |  | x |  |  |  | x |  | x | x |  |  |  |
| Lipids | Lyso-PI 16:0 | 12.759 | 571.28992 | [M-H]^-^ | 3 | [-] | x |  | x |  | x |  |  |  |  | x | x |  |  |
| Lipids | Lyso-PI 18:1 | 12.864 | 597.30463 | [M-H]^-^ | 3 | [-] | x |  |  |  |  |  |  |  |  | x | x |  |  |
| Lipids | Lyso-PI 18:2 | 12.482 | 595.29523 | [M-H]^-^ | 3 | [-] | x |  |  |  | x |  |  |  |  | x | x |  |  |
| Lipids | Lyso-PI 18:3 | 12.313 | 593.2796 | [M-H]^-^ | 3 | [-] | x |  |  |  |  |  |  |  |  | x | x |  |  |
| Other acid compounds | Malic acid | 1.089 | 133.01448 | [M-H]^-^ | 3 | [-] |  | x |  |  |  | x | x |  |  |  |  |  |  |
| Other acid compounds | Malic acid | 0.728 | 133.01431 | [M-H]^-^ | 3 | [-] | x | x | x |  | x | x | x |  |  |  |  |  |  |
| Other acid compounds | Malic acid | 0.901 | 133.0154 | [M-H]^-^ | 3 | [-] |  |  | x |  |  | x |  |  |  |  |  |  |  |
| Terpenes & Terpenoids | Maslinic acid | 12.436 | 471.34879 | [M-H]^-^ | 3 | [-] | x |  |  |  |  |  |  |  |  | x | x |  |  |
| Terpenes & Terpenoids | Maslinic acid | 12.676 | 471.35187 | [M-H]^-^ | 3 | [-] | x |  |  |  |  |  |  |  |  |  | x |  |  |
| Other acid compounds | Meglutol | 1.062 | 161.04581 | [M-H]^-^ | 3 | [-] | x |  |  |  |  |  | x |  |  |  |  |  |  |
| Terpenes & Terpenoids | Menthenyl-Hexoside | 4.027 | 355.17233 | [M+Na]^+^ | 3 | [+] | x |  |  |  |  |  |  |  | x | x |  |  |  |
| Terpenes & Terpenoids | Menthenyl-Hexoside | 4.317 | 355.17264 | [M+Na]^+^ | 3 | [+] | x |  |  |  |  |  |  |  |  | x |  |  |  |
| Terpenes & Terpenoids | Menthenyl-Hexoside | 4.744 | 355.17273 | [M+Na]^+^ | 3 | [+] | x |  | x |  |  |  |  |  |  | x |  |  |  |
| Phenolic compounds | Methoxyhydroxybenzoic acid-Hexoside | 3.255 | 359.10104 | [M-H]^-^ | 3 | [-] |  |  |  | x | x |  | x |  |  |  |  |  |  |
| Phenolic compounds | Methoxysalicylic acid | 4.021 | 167.03622 | [M-H]^-^ | 3 | [-] |  | x |  |  |  |  |  | x | x |  |  |  |  |
| Amino compounds | Methoxytyrosine | 3.531 | 210.0789 | [M-H]^-^ | 3 | [-] |  | x |  |  |  |  | x | x | x |  |  |  |  |
| Other nitrogen compounds | Methylthioadenosine | 3.246 | 298.09637 | [M+H]^+^ | 3 | [+] | x |  |  |  |  |  |  |  | x |  |  |  |  |
| Amino compounds | N-Acetylleucine | 3.488 | 172.0984 | [M-H]^-^ | 3 | [-] |  | x |  |  |  |  | x | x |  |  |  |  |  |
| Others | N-Acetylleucine | 2.737 | 172.0986 | [M-H]^-^ | 3 | [-] | x |  |  |  |  | x |  |  |  |  |  |  |  |
| Flavonoids | Naringenin derivate | 5.498 | 271.06485 | [M-H]^-^ | 3 | [-] | x |  |  |  |  |  |  |  | x |  |  |  |  |
| Flavonoids | Naringenin-Hexoside | 4.238 | 433.11752 | [M-H]^-^ | 3 | [-] | x | x |  |  |  |  |  |  | x |  |  |  |  |
| Flavonoids | Naringenin-Hexoside | 4.466 | 433.11752 | [M-H]^-^ | 3 | [-] | x |  |  |  |  |  |  |  | x |  |  |  |  |
| Flavonoids | Naringenin-Hexoside | 7.527 | 433.11795 | [M-H]^-^ | 3 | [-] | x |  |  |  |  |  |  |  | x |  |  |  |  |
| Amino compounds | Niacinamide | 0.915 | 123.05494 | [M+H]^+^ | 3 | [+] | x |  | x |  | x |  | x | x | x |  |  |  |  |
| Other acid compounds | Nicotinic acid derivate | 0.703 | 124.03934 | [M+H]^+^ | 3 | [+] |  | x |  |  |  | x | x |  |  |  |  |  |  |
| Phenolic compounds | Noradrenaline | 2.246 | 152.07039 | [M-H_2_O+H]^+^ | 3 | [+] |  |  | x |  |  |  |  |  | x |  |  |  |  |
| Amino compounds | N-Trimethyllysine | 0.611 | 189.15976 | [M]^+^ | 3 | [+] |  |  | x |  |  | x | x |  |  |  |  |  |  |
| Phenolic compounds | Octadecanedioic acid | 12.702 | 313.24066 | [M-H]^-^ | 3 | [-] |  |  |  |  | x |  |  |  |  | x |  |  |  |
| Phenolic compounds | Orsellinic acid | 3.666 | 167.0354 | [M-H]^-^ | 3 | [-] |  | x |  |  |  |  |  |  | x |  |  |  |  |
| Coumarins | Osthole | 12.159 | 245.11687 | [M+H]^+^ | 3 | [+] |  |  | x |  |  |  |  |  |  |  | x |  |  |
| Amino compounds | Oxindole | 1.769 | 134.05986 | [M+H]^+^ | 3 | [+] |  |  | x |  |  |  | x | x | x |  |  |  |  |
| Other acid compounds | Oxononanoic acid | 5.915 | 171.10312 | [M-H]^-^ | 3 | [-] |  |  |  |  | x |  |  |  | x |  |  |  |  |
| Lipids | PC 18:0 | 14.569 | 524.36987 | [M+H]^+^ | 3 | [+] |  |  | x |  |  |  |  |  |  |  |  |  | x |
| Phenolic compounds | p-Coumaraldehyde | 3.278 | 147.0464 | [M-H]^-^ | 3 | [-] |  | x |  |  |  |  |  |  | x |  |  |  |  |
| Coumarins | Phellopterin | 6.316 | 515.12457 | [M-H]^-^ | 3 | [-] | x |  | x |  |  |  |  |  | x |  |  |  |  |
| Coumarins | Phellopterin | 7.235 | 515.11908 | [M-H]^-^ | 3 | [-] |  |  | x |  |  |  |  |  | x |  |  |  |  |
| Others | Phenylacetaldehyde | 1.183 | 121.06452 | [M+H]^+^ | 3 | [+] |  | x | x |  |  |  | x | x | x |  |  |  |  |
| Other acid compounds | Phenylacetic acid | 1.283 | 135.04631 | [M-H]^-^ | 3 | [-] |  | x |  |  |  |  | x | x |  |  |  |  |  |
| Other acid compounds | Phenylacetic acid | 2.992 | 135.04518 | [M-H]^-^ | 3 | [-] | x | x |  |  | x |  | x | x |  |  |  |  |  |
| Other acid compounds | Phenylacetic acid | 3.577 | 135.04538 | [M-H]^-^ | 3 | [-] |  |  |  |  | x |  | x | x |  |  |  |  |  |
| Other acid compounds | Phenylacetic acid | 4.310 | 135.04527 | [M-H]^-^ | 3 | [-] |  |  | x |  |  |  |  |  |  | x |  |  |  |
| Other acid compounds | Phenylacetic acid | 5.124 | 135.04535 | [M-H]^-^ | 3 | [-] | x |  | x |  |  |  |  |  | x |  |  |  |  |
| Amino compounds | Phenylalanine derivate | 0.877 | 166.08629 | [M+H]^+^ | 3 | [+] |  |  | x |  |  |  | x |  |  |  |  |  |  |
| Amino compounds | Phenylalanine derivate | 1.074 | 166.086 | [M+H]^+^ | 3 | [+] | x |  |  |  |  | x | x |  |  |  |  |  |  |
| Amino compounds | Phenylalanine derivate | 1.218 | 164.07355 | [M-H]^-^ | 3 | [-] | x |  | x |  |  |  | x | x |  |  |  |  |  |
| Amino compounds | Phenylalanine derivate | 1.591 | 164.07208 | [M-H]^-^ | 3 | [-] |  |  |  |  | x | x |  |  |  |  |  |  |  |
| Amino compounds | Phenylalanine derivate | 3.503 | 166.08595 | [M+H]^+^ | 3 | [+] |  | x |  |  |  |  |  |  | x |  |  |  |  |
| Others | Pheophorbide A | 15.324 | 593.27551 | [M+H]^+^ | 3 | [+] | x |  | x |  |  |  |  |  |  |  |  | x |  |
| Flavonoids | Phloretin-Hexoside | 6.842 | 435.13211 | [M-H]^-^ | 3 | [-] |  | x |  |  | x |  |  |  | x |  |  |  |  |
| Lipids | Phosphocholine | 0.694 | 184.07295 | [M]^+^ | 3 | [+] | x |  |  |  |  | x |  |  |  |  |  |  |  |
| Lipids | Phosphocholine | 0.838 | 184.0731 | [M]^+^ | 3 | [+] | x |  |  |  |  | x |  |  |  |  |  |  |  |
| Lipids | Phytosphingosine | 14.552 | 318.30038 | [M+H]^+^ | 3 | [+] |  |  |  |  | x |  |  |  |  |  |  | x |  |
| Lipids | PI 16:0/18:3 | 15.697 | 831.50336 | [M-H]^-^ | 3 | [-] | x |  |  |  |  |  |  |  |  | x | x | x |  |
| Lipids | PI 34:2 | 15.996 | 833.51855 | [M-H]^-^ | 3 | [-] | x |  |  |  |  |  |  |  |  |  | x | x | x |
| Terpenes & Terpenoids | Picrocrocin-Hexoside | 4.149 | 411.19925 | [M+Na]^+^ | 3 | [+] |  |  | x |  |  |  |  |  | x |  |  |  |  |
| Phenolic compounds | Piscidic acid | 1.177 | 255.0517 | [M-H]^-^ | 3 | [-] |  |  |  |  | x | x |  |  |  |  |  |  |  |
| Phenolic compounds | Piscidic acid | 2.171 | 255.05208 | [M-H]^-^ | 3 | [-] |  |  |  | x | x |  | x |  |  |  |  |  |  |
| Amino compounds | p-Octopamine | 1.017 | 136.07541 | [M-H_2_O+H]^+^ | 3 | [+] | x | x | x |  |  |  | x | x |  |  |  |  |  |
| Amino compounds | Proline betaine | 0.758 | 144.10211 | [M+H]^+^ | 3 | [+] |  |  |  |  | x | x | x |  |  |  |  |  |  |
| Phenolic compounds | Protocatechuic acid | 2.588 | 153.01952 | [M-H]^-^ | 3 | [-] | x | x |  |  |  |  | x | x |  |  |  |  |  |
| Phenolic compounds | Pyrocatechol | 1.904 | 109.0297 | [M-H]^-^ | 3 | [-] |  |  | x |  |  |  |  |  |  |  |  |  |  |
| Phenolic compounds | Pyrogallol | 1.516 | 125.02475 | [M-H]^-^ | 3 | [-] | x | x |  |  |  |  | x | x |  |  |  |  |  |
| Other acid compounds | Pyruvate | 3.8 | 87.00946 | [M-H]^-^ | 3 | [-] | x |  |  |  |  |  | x |  |  |  |  |  |  |
| Flavonoids | Quercetin | 9.389 | 301.03708 | [M-H]^-^ | 3 | [-] | x | x | x |  |  |  |  |  |  | x |  |  |  |
| Flavonoids | Quercetin-Acetylhexoside | 7.566 | 505.09888 | [M-H]^-^ | 3 | [-] | x |  |  |  |  |  |  |  |  | x |  |  |  |
| Flavonoids | Quercetin-O-dihexoside | 3.562 | 627.15448 | [M+H]^+^ | 3 | [+] | x |  |  |  |  |  | x |  |  |  |  |  |  |
| Flavonoids | Quercetin-O-hexosyl-deoxyhexoside | 6.688 | 609.14996 | [M-H]^-^ | 3 | [-] | x |  |  |  |  |  |  |  | x |  |  |  |  |
| Flavonoids | Quercetin-O-hexosyl-deoxyhexoside | 11.6 | 609.14929 | [M-H]^-^ | 3 | [-] |  |  | x |  |  |  |  |  |  | x |  |  |  |
| Flavonoids | Quercetin-O-hexosyl-pentoside | 4.4 | 595.13501 | [M-H]^-^ | 3 | [-] | x |  | x |  |  |  |  |  | x |  |  |  |  |
| Flavonoids | Quercetin-O-hexosyl-pentoside | 4.64 | 595.13519 | [M-H]^-^ | 3 | [-] |  |  | x |  |  |  |  |  | x |  |  |  |  |
| Other acid compounds | Quinic Acid | 3.802 | 191.05775 | [M-H]^-^ | 3 | [-] |  |  | x |  |  |  |  | x |  |  |  |  |  |
| Flavonoids | Rhamnetin-dihexoside | 3.837 | 641.17151 | [M+H]^+^ | 3 | [+] | x |  |  |  |  |  |  |  | x |  |  |  |  |
| Phenolic compounds | Salicin-Derivat | 4.968 | 539.18134 | [M-H]^-^ | 3 | [-] |  |  |  |  | x |  |  |  | x | x |  |  |  |
| Alkaloids | Salsolinol | 1.713 | 180.10173 | [M+H]^+^ | 3 | [+] | x | x | x |  |  |  | x | x | x |  |  |  |  |
| Other acid compounds | Sambunigrin | 3.483 | 294.10086 | [M-H]^-^ | 3 | [-] |  |  | x |  |  |  |  |  |  |  |  |  |  |
| Other acid compounds | Shikimic acid | 3.291 | 173.04709 | [M-H]^-^ | 3 | [-] | x |  |  |  |  | x |  |  |  |  |  |  |  |
| Other acid compounds | Shikimic acid | 3.751 | 173.04599 | [M-H]^-^ | 3 | [-] | x |  |  |  |  |  | x | x |  |  |  |  |  |
| Other acid compounds | Stachyose | 0.621 | 665.21564 | [M-H]^-^ | 3 | [-] |  |  |  |  | x | x | x |  |  |  |  |  |  |
| Lipids | Stigmastadienone | 15.85 | 411.36188 | [M+H]^+^ | 3 | [+] |  |  |  |  | x |  |  |  |  |  |  |  | x |
| Other acid compounds | Suberic acid | 3.936 | 173.08199 | [M-H]^-^ | 3 | [-] |  |  |  |  | x |  |  | x |  |  |  |  |  |
| Phenolic compounds | Syringyl-Hexoside | 3.511 | 395.13077 | [M+Na]^+^ | 3 | [+] |  |  |  |  | x |  | x | x | x |  |  |  |  |
| Carbohydrates | Threonic acid | 3.229 | 135.03123 | [M-H]^-^ | 3 | [-] |  |  | x |  |  |  | x |  |  |  |  |  |  |
| Carbohydrates | Threonic acid | 3.647 | 135.0313 | [M-H]^-^ | 3 | [-] |  |  | x |  |  |  |  | x |  |  |  |  |  |
| Carbohydrates | Threonic acid | 4.005 | 135.03008 | [M-H]^-^ | 3 | [-] |  |  | x |  |  |  |  |  | x |  |  |  |  |
| Carbohydrates | Threonic acid | 3.344 | 135.03102 | [M-H]^-^ | 3 | [-] |  |  | x |  |  |  |  | x |  |  |  |  |  |
| Alkaloids | Trigonelline | 0.735 | 138.05469 | [M+H]^+^ | 3 | [+] |  | x | x |  |  | x | x |  |  |  |  |  |  |
| Alkaloids | Trigonelline | 3.523 | 138.05486 | [M+H]^+^ | 3 | [+] |  | x |  |  |  |  |  |  | x |  |  |  |  |
| Lipids | trihydroxy-octadecadienoic acid | 11.003 | 327.21054 | [M-H]^-^ | 3 | [-] |  |  | x |  |  |  |  |  |  | x |  |  |  |
| Lipids | trihydroxy-octadecadienoic acid | 12.383 | 295.23077 | [M-H]^-^ | 3 | [-] | x |  |  |  | x |  |  |  | x | x |  |  |  |
| Lipids | Trihydroxy-octadecenoic acid | 11.699 | 329.23718 | [M-H]^-^ | 3 | [-] | x | x | x | x | x |  |  |  |  | x |  |  |  |
| Amino compounds | Tyramine | 2.92 | 121.06446 | [M+H-NH_3_]^+^ | 3 | [+] | x | x |  |  |  |  |  |  | x |  |  |  |  |
| Amino compounds | Tyramine | 3.61 | 121.06485 | [M+H-NH_3_]^+^ | 3 | [+] | x |  |  |  |  |  |  | x | x |  |  |  |  |
| Amino compounds | Tyramine | 4.995 | 121.06478 | [M+H-NH_3_]^+^ | 3 | [+] | x |  |  |  | x |  |  |  |  | x |  |  |  |
| Amino compounds | Tyramine | 6.828 | 121.06478 | [M+H-NH_3_]^+^ | 3 | [+] |  |  |  |  | x |  |  |  |  | x |  |  |  |
| Carbohydrates | Tyrosol-Hexoside | 3.762 | 439.15704 | [M+Na]^+^ | 3 | [+] |  |  | x |  |  |  |  |  | x |  |  |  |  |
| Coumarins | Umbelliferone | 3.261 | 161.02483 | [M-H]^-^ | 3 | [-] |  |  | x |  |  |  | x |  |  |  |  |  |  |
| Coumarins | Umbelliferone | 3.534 | 161.02606 | [M-H]^-^ | 3 | [-] | x |  | x |  |  |  | x | x |  |  |  |  |  |
| Coumarins | Umbelliferone | 4.859 | 161.0258 | [M-H]^-^ | 3 | [-] | x |  |  |  |  |  |  | x | x |  |  |  |  |
| Coumarins | Umbelliferone | 6.369 | 161.02599 | [M-H]^-^ | 3 | [-] | x |  | x |  |  |  |  |  | x |  |  |  |  |
| Other acid compounds | Undecanedioic acid | 8.433 | 215.13084 | [M-H]^-^ | 3 | [-] | x |  | x |  | x |  |  |  | x |  |  |  |  |
| Other acid compounds | Undecanedioic acid | 8.511 | 215.12923 | [M-H]^-^ | 3 | [-] |  | x |  |  |  |  |  |  | x |  |  |  |  |
| Amino compounds | Uracil | 0.603 | 113.0358 | [M-H]^-^ | 3 | [-] |  |  |  |  | x |  |  |  |  |  |  |  |  |
| Other nitrogen compounds | Uridine | 0.794 | 243.06242 | [M-H]^-^ | 3 | [-] |  |  |  |  | x |  | x |  |  |  |  |  |  |
| Terpenes & Terpenoids | Ursolic acid epoxide | 12.398 | 437.34146 | [M-H_2_O+H]^+^ | 3 | [+] | x |  |  |  |  |  |  |  |  |  | x |  |  |
| Phenolic compounds | Vanillic acid | 1.633 | 167.03651 | [M-H]^-^ | 3 | [-] |  |  |  |  | x | x |  |  |  |  |  |  |  |
| Terpenes & Terpenoids | Verbenalin Derivate | 3.965 | 487.21417 | [M+Na]^+^ | 3 | [+] |  |  | x |  |  |  |  |  | x |  |  |  |  |
| Phenolic compounds | Vinylphenol | 3.325 | 119.05042 | [M-H]^-^ | 3 | [-] |  | x |  |  |  |  |  | x |  |  |  |  |  |

**Figures**





**Fig. S1.** Preparative LC chromatograms of plant extracts with absorbances up to 4000 mAU measured at 330 nm, color coded according to plant part for comparison between species with green for leaves, orange for flowers, purple for buds and herb, blue for stems, black for seeds, brown for roots and barks.


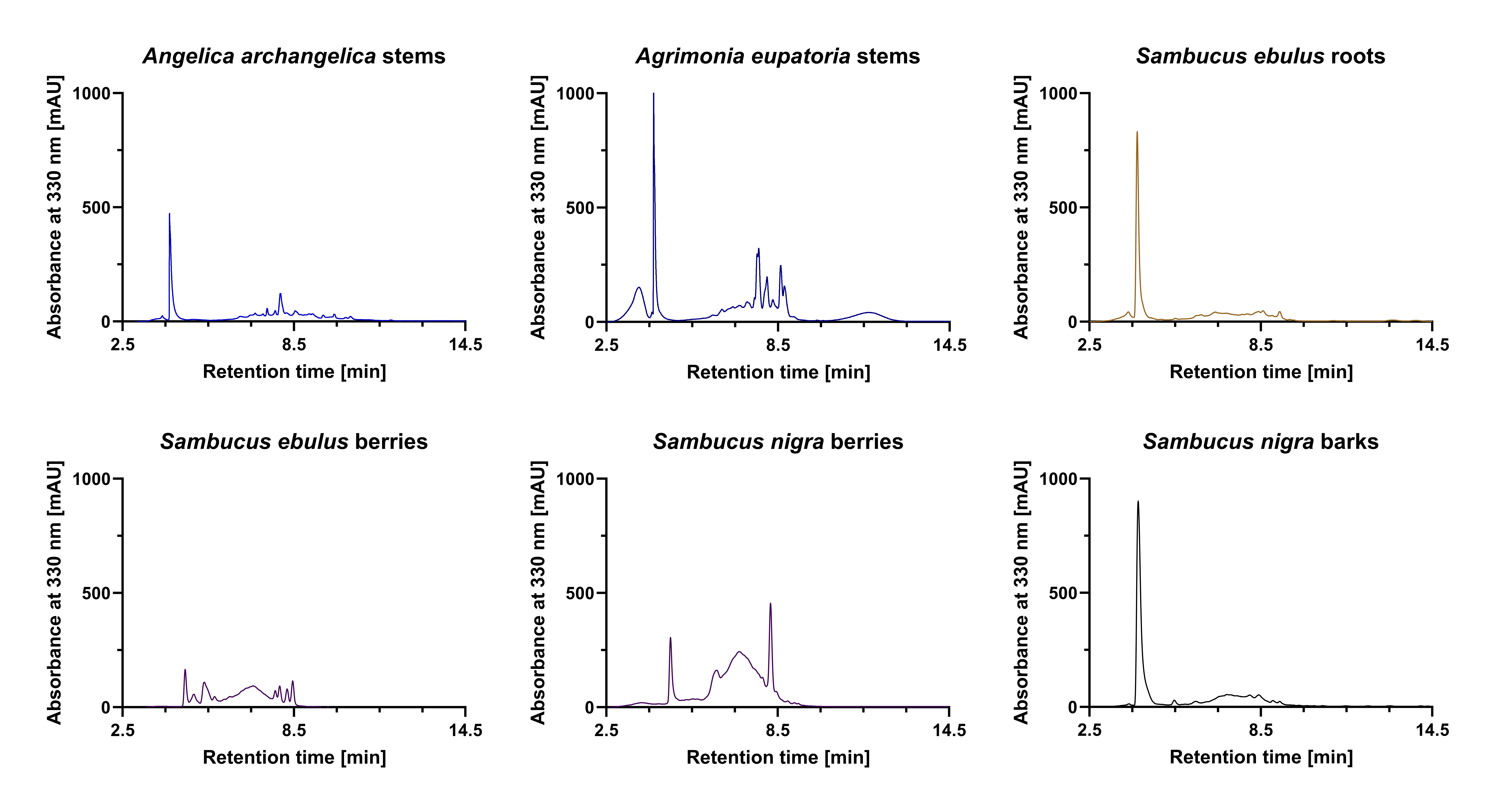


**Fig. S2.** Preparative LC chromatograms of plant extracts with absorbances up to 1000 mAU measured at 330 nm, color coded according to plant part for comparison between species with blue for stems, purple for berries, and brown for roots and barks.





**Fig. S3.** TICs (black) and BPCs (blue) of the total HPLC run of the ten fractions analyzed by HPLC-HRMS.


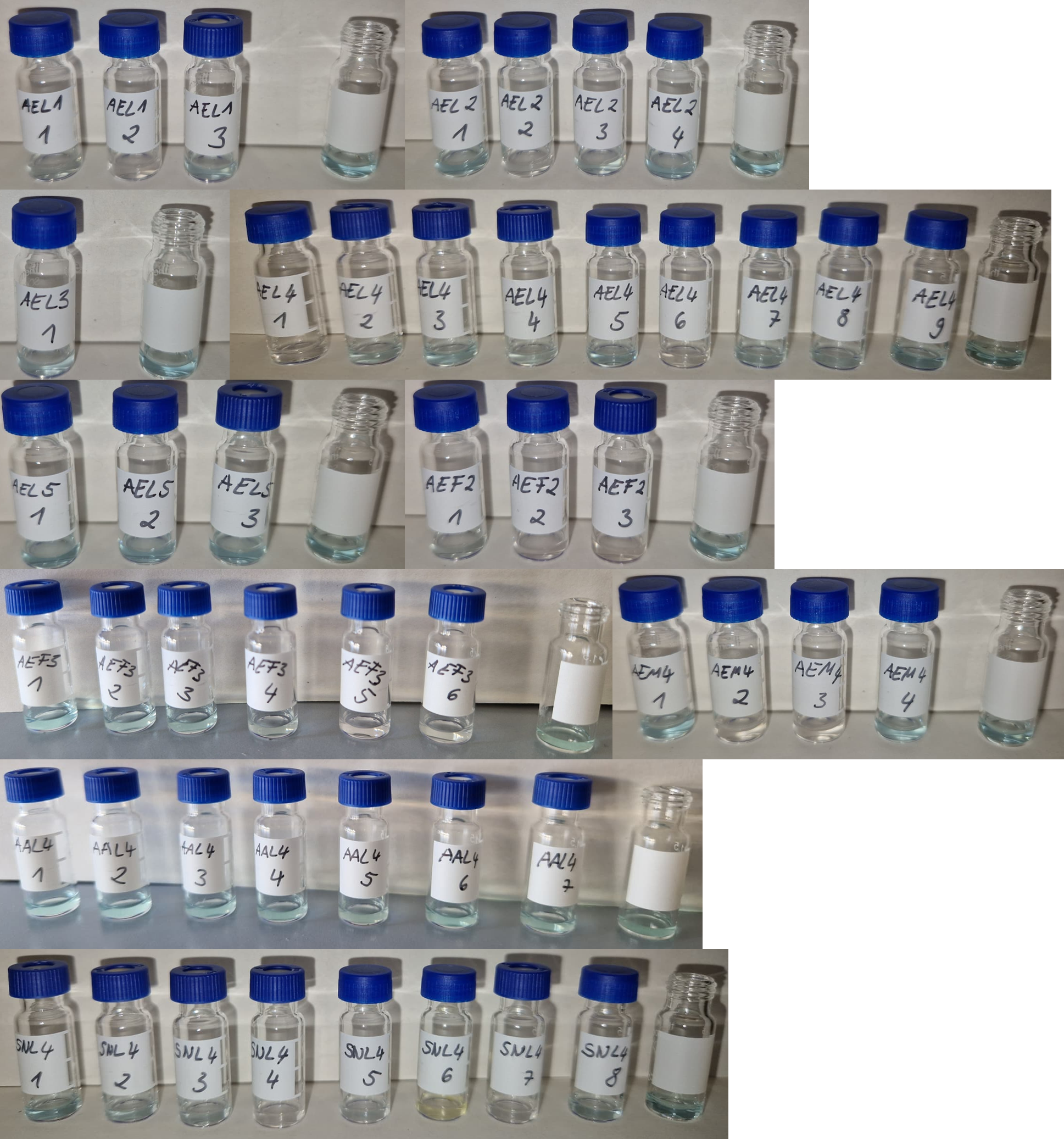


**Fig. S4.** Overview of all qualitative color reactions with ABTS of selective fractions separated by HPLC. A blue color indicates no reaction with ABTS, a fading of the blue color resulting in invisible or yellow color indicates antioxidant activity. The vial on the right of each picture is the corresponding control where only the reagent was added with the same concentration and volume as for every other vial. The order of experiments is as follows, from left to right, top to bottom: *A. eupatoria* leaves prep LC fraction 1 (AEL1), *A. eupatoria* leaves prep LC fraction 2 (AEL2), *A. eupatoria* leaves prep LC fraction 3 (AEL3), *A. eupatoria* leaves prep LC fraction 4 (AEL4), *A. eupatoria* leaves prep LC fraction 5 (AEL5), *A. eupatoria* flowers prep LC fraction 2 (AEF2), *A. eupatoria* flowers prep LC fraction 3 (AEF3), *A. eupatoria* stems prep LC fraction 4 (AEM4), *A. angelica* leaves prep LC fraction 4 (AAL4), and *S. nigra* leaves prep LC fraction 4 (SNL4). The numbering below the labeling refers to the number of fractions collected by HPLC as indicated with boxes in Fig. 4.


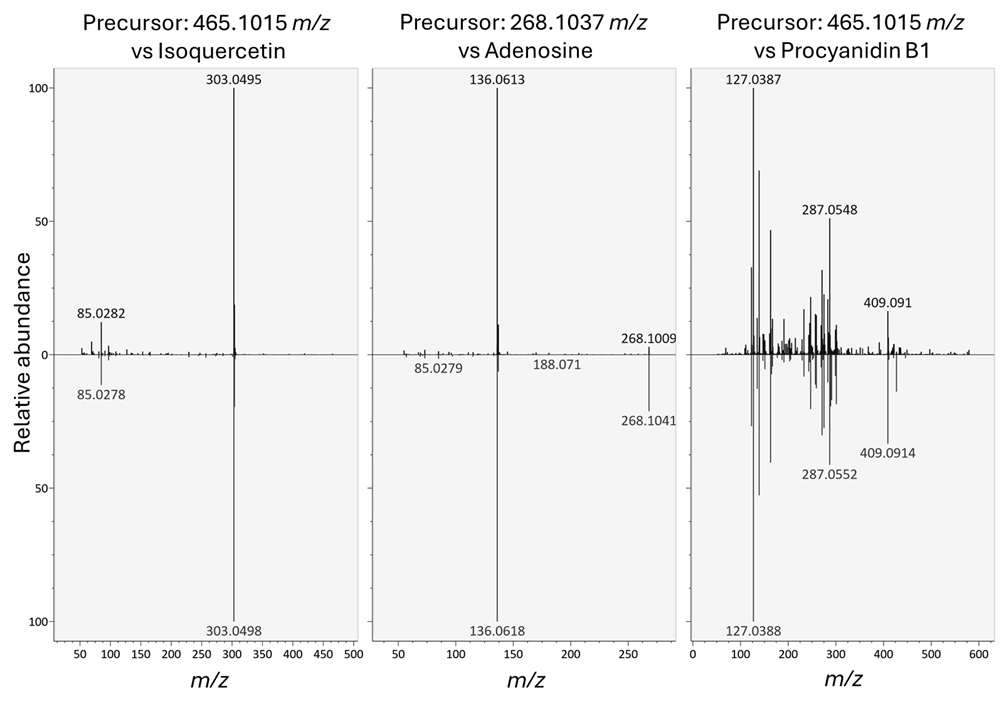


**Fig. S5.** Direct comparison of MS/MS spectra for isoquercetin, adenosine, and procyanidin B1. For each compound, the upper panel shows the experimental precursor-ion fragmentation spectrum, while the lower panel displays the corresponding library-match spectrum used for identification. Additional MS/MS spectra and MS spectra are provided in Supplementary Figure S6 and S7.


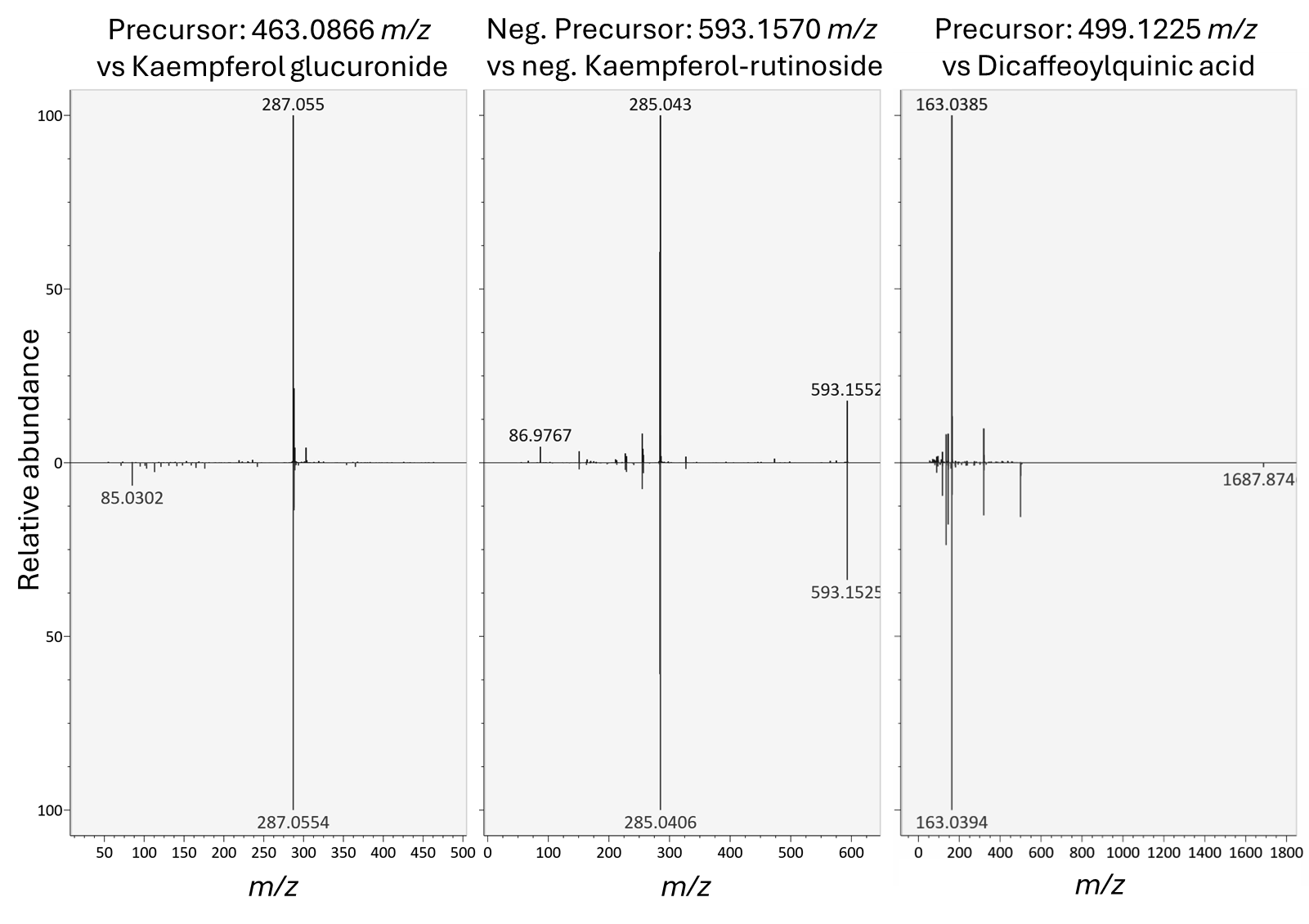


**Fig. S6.** Direct comparison of MS/MS spectra for the three annotated components. For each compound, the upper panel shows the experimental precursor-ion fragmentation spectrum, while the lower panel displays the corresponding library-match spectrum used for identification. Spectral comparison is shown for kaempferol glucuronide, kaempferol-rutinoside (negative mode), and dicaffeoylquinic acid.


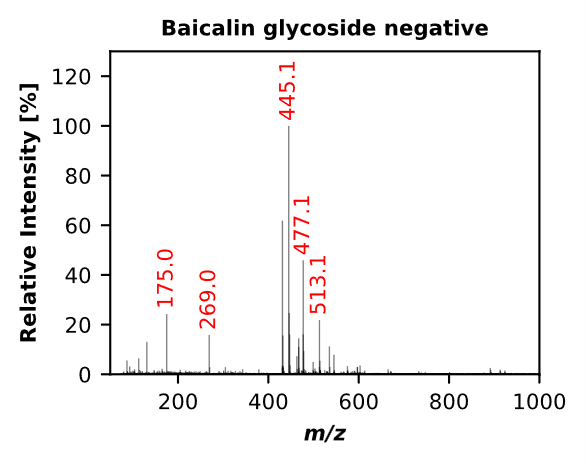

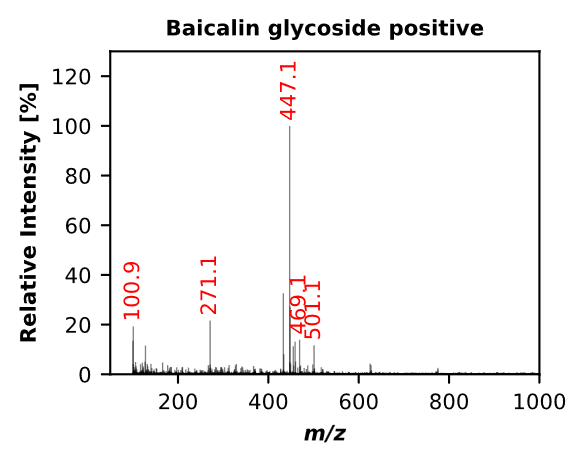

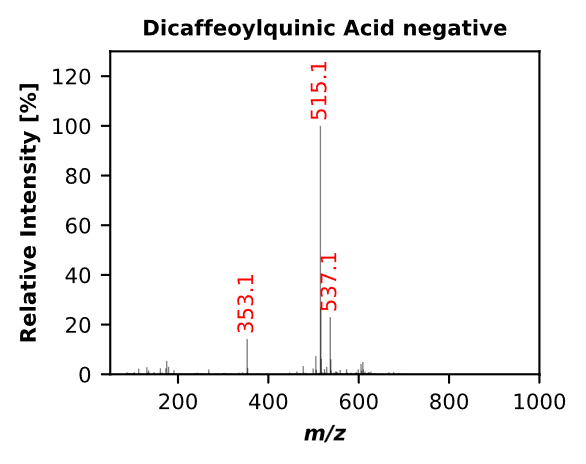

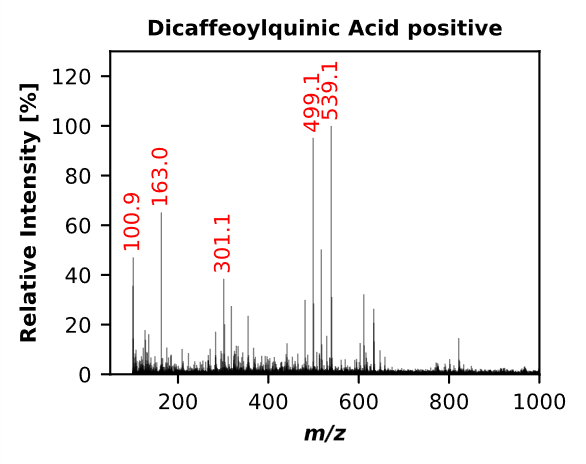

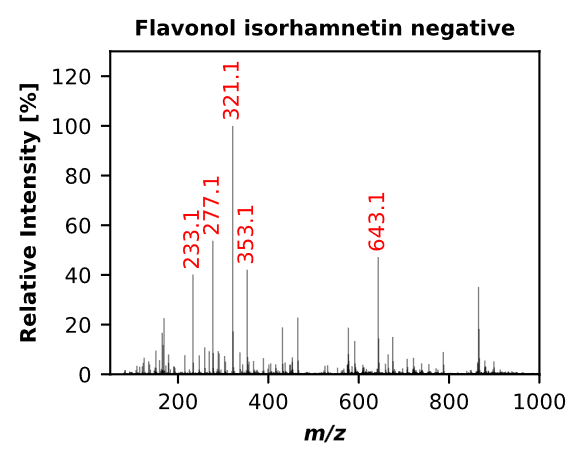

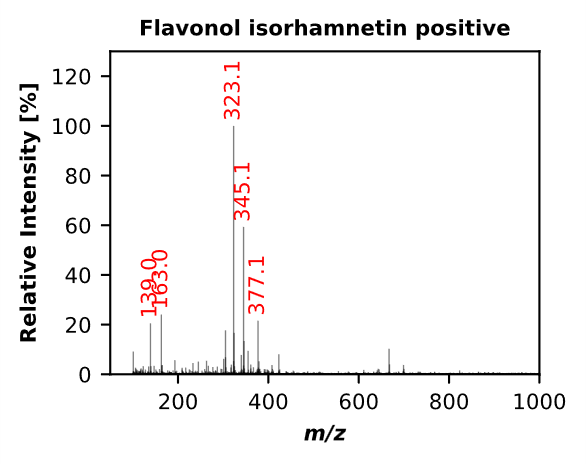

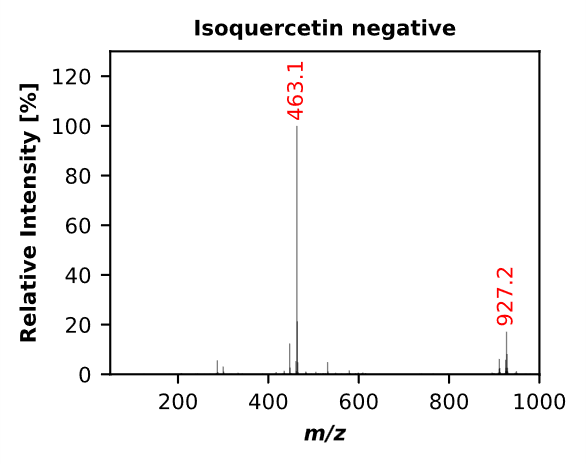

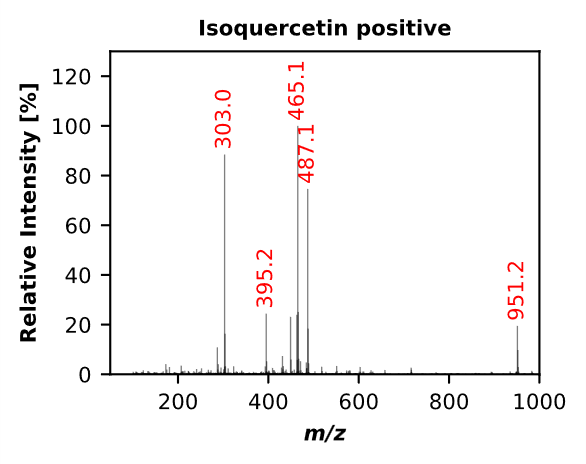

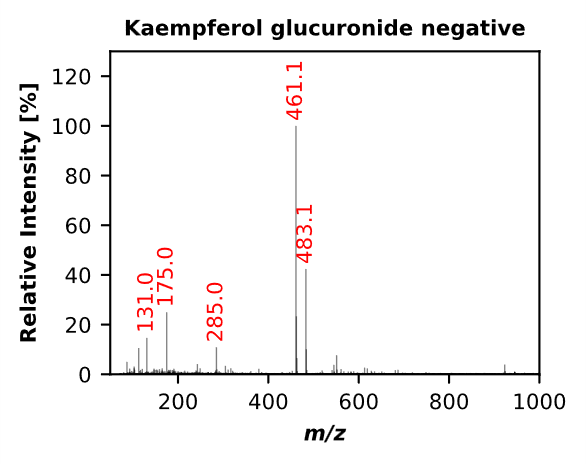

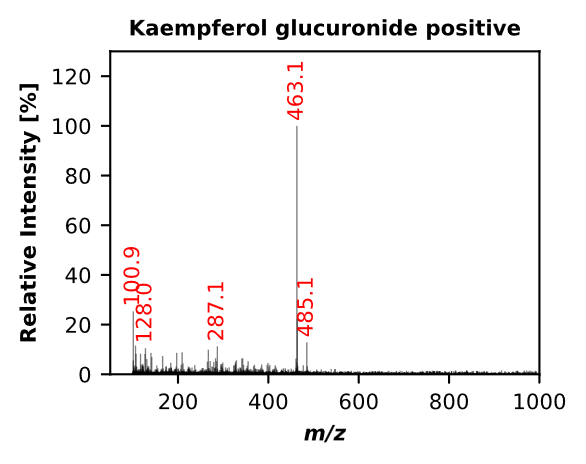

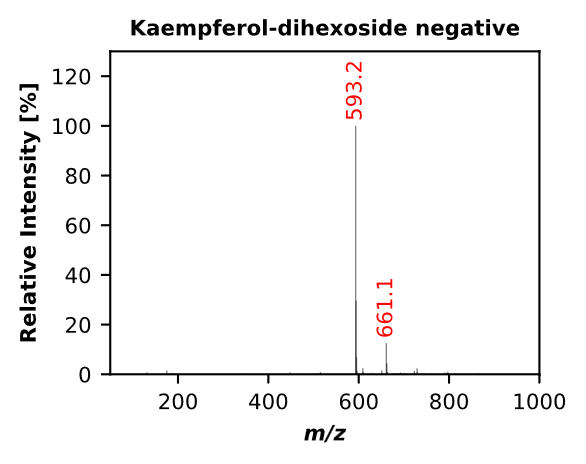

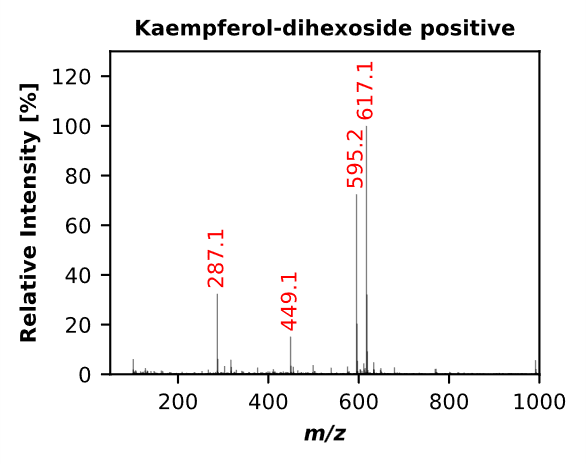

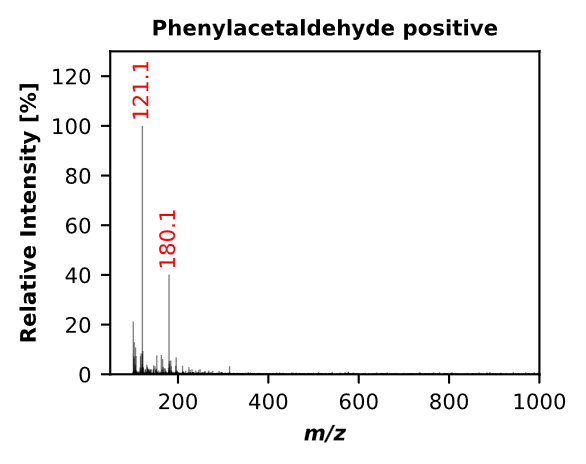

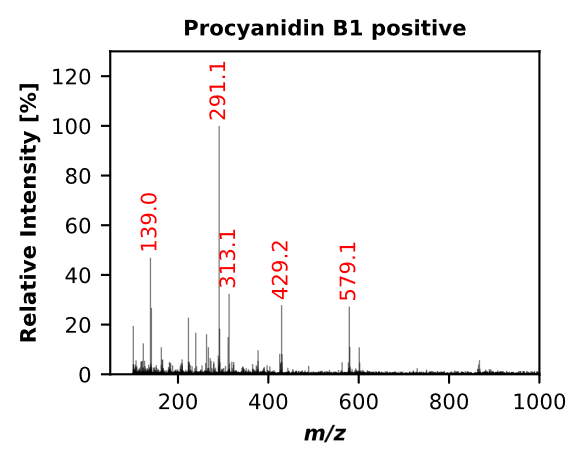

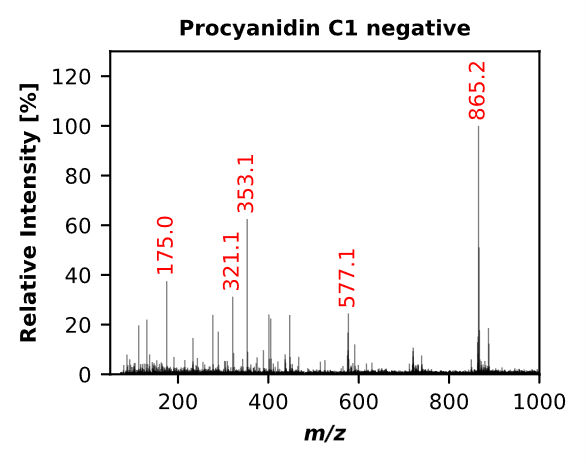

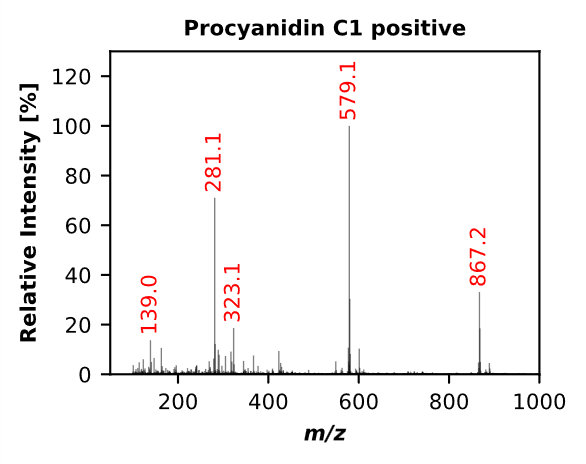

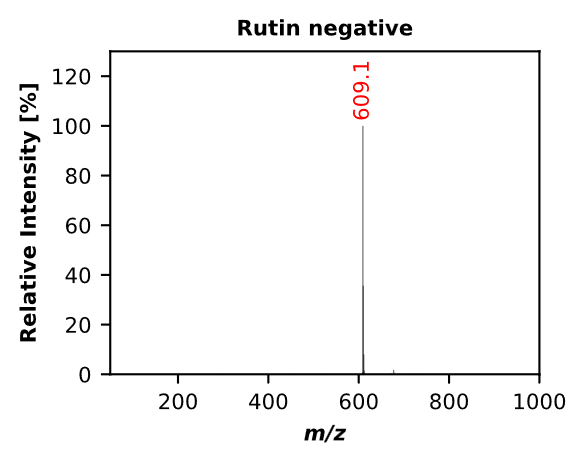

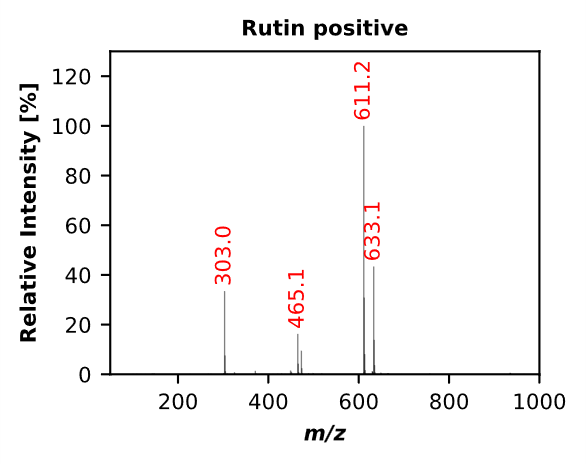

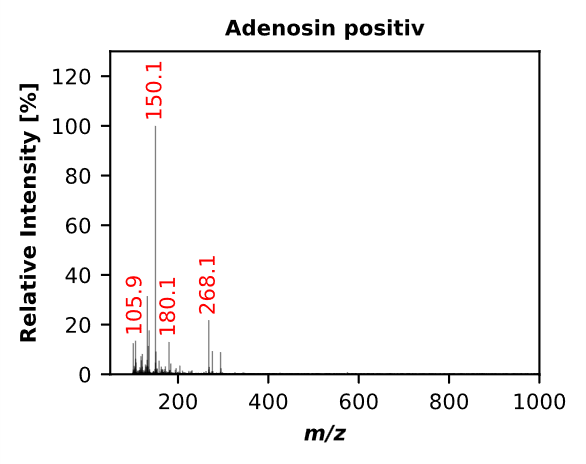

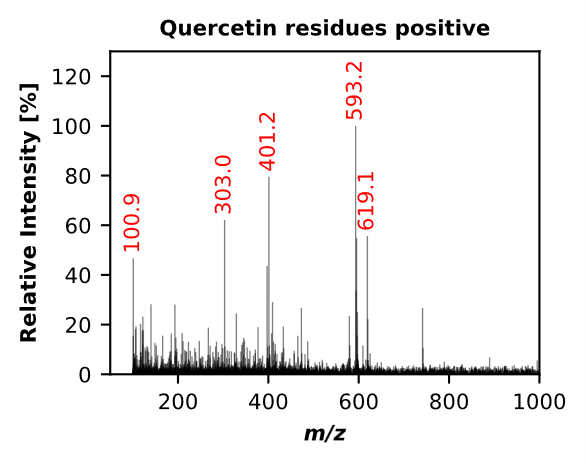


**Fig. S7.** Direct comparison of MS spectra in positive and negative mode, if acquired, of compounds tentatively identified in the fractions from Table 1.


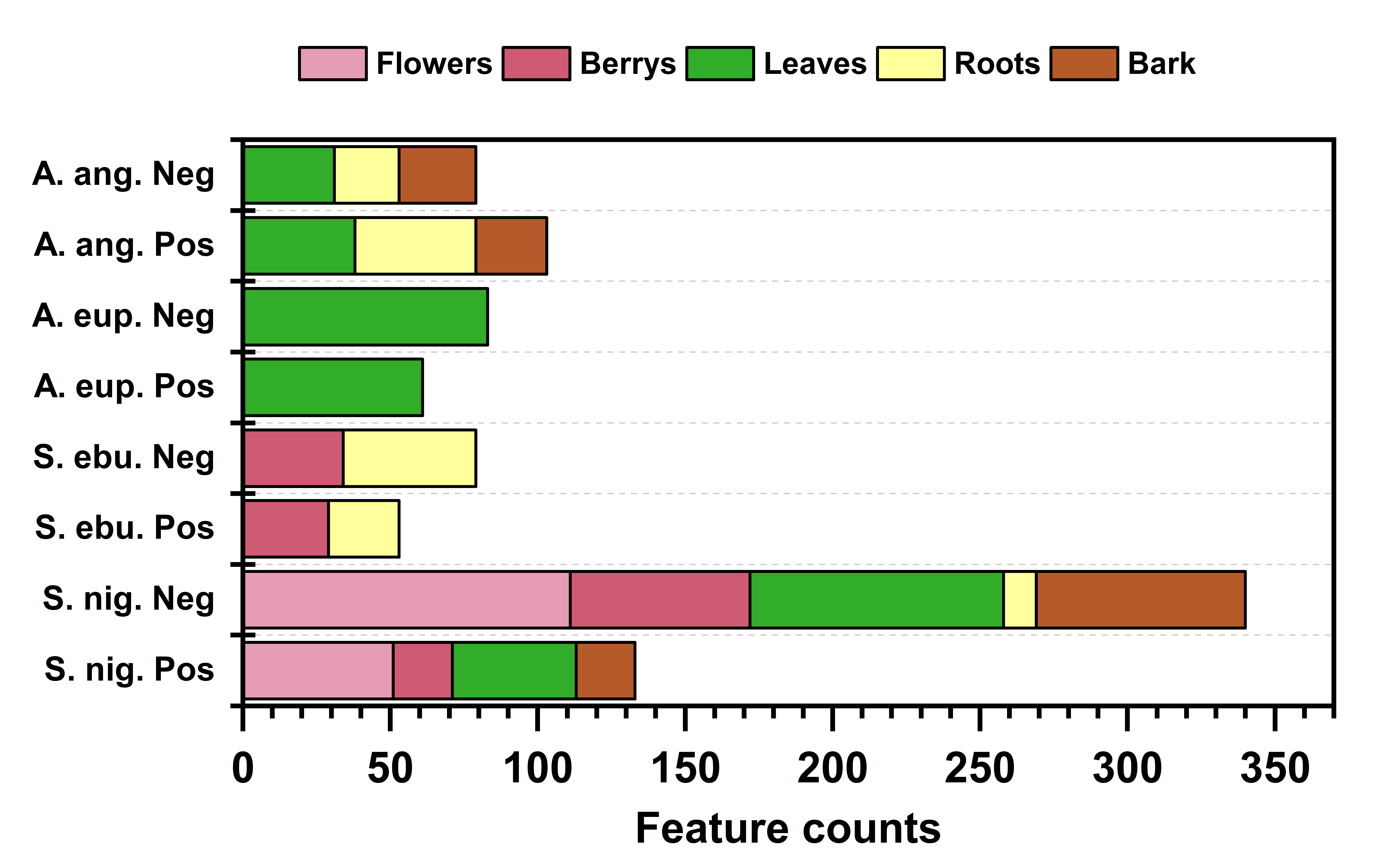


**Fig. S8.** Distribution of tentatively identified features among the analyzed plant parts. For *A. archangelica*, 31 negative and 37 positive features were found in leaves, 22 negative and 41 positive in roots, and 26 negative and 24 positive in bark. In *A. eupatoria*, 61 negative and 83 positive features were detected in the leaves. In *S. ebulus*, 34 negative and 29 positive features were found in berries, and 44 negative and 23 positive in roots. For *S. nigra*, the following were observed, for flowers 111 negative, 51 positive, berries 61 negative, 20 positive, leaves 86 negative, 42 positive, roots 11 negative, 0 positive, and bark 71 negative, 20 positive.


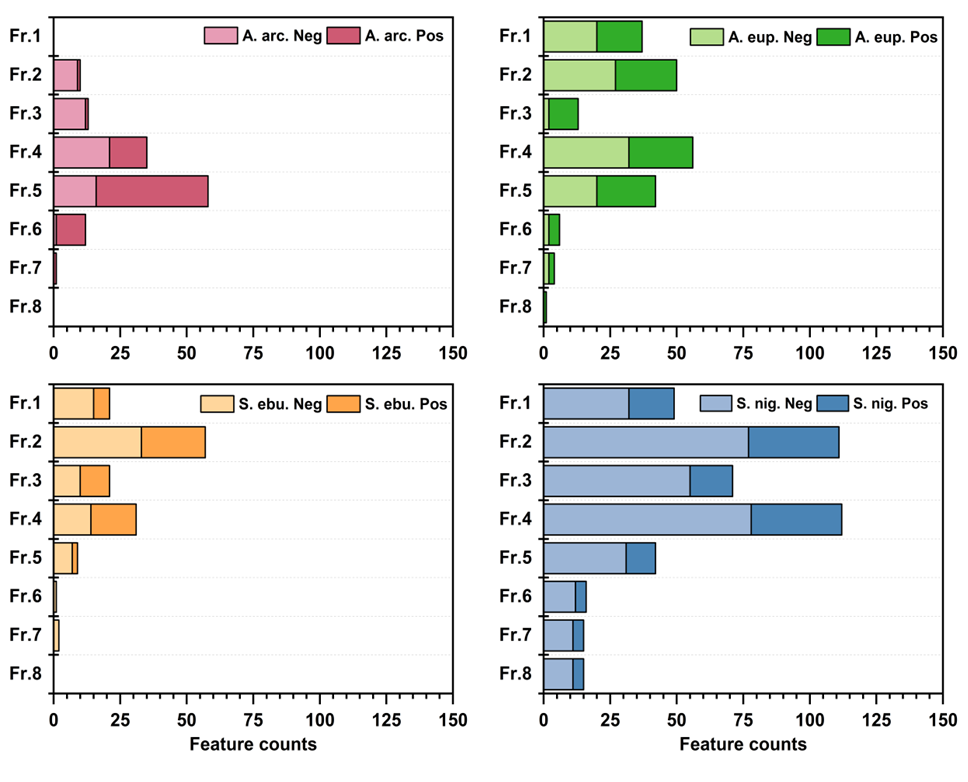


**Fig. 9.** Feature distribution across the semi-preparative LC fractions with both ionization modes. Fractions 2 and 4 show the highest number of detected features. Small amounts of features are seen for fractions 6, 7 and 8 correlating to the trend already seen in Table 1. The distribution of tentatively identified features among the analyzed plant parts is shown in the Supplementary Fig. S7.
